# Global, regional, and national burden of upper respiratory infections and otitis media, 1990–2021: a systematic analysis from the Global Burden of Disease Study 2021

**DOI:** 10.1016/S1473-3099(24)00430-4

**Published:** 2025-01

**Authors:** GBD 2021 Upper Respiratory Infections Otitis Media Collaborators, Sarah Brooke Sirota, Matthew C Doxey, Regina-Mae Villanueva Dominguez, Rose Grace Bender, Avina Vongpradith, Samuel B Albertson, Amanda Novotney, Katrin Burkart, Austin Carter, Parsa Abdi, Meriem Abdoun, Ayele Mamo Abebe, Kedir Hussein Abegaz, Richard Gyan Aboagye, Hassan Abolhassani, Lucas Guimarães Abreu, Hasan Abualruz, Eman Abu-Gharbieh, Salahdein Aburuz, Mesafint Molla Adane, Isaac Yeboah Addo, Victor Adekanmbi, Qorinah Estiningtyas Sakilah Adnani, Leticia Akua Adzigbli, Muhammad Sohail Afzal, Saira Afzal, Bright Opoku Ahinkorah, Sajjad Ahmad, Ayman Ahmed, Haroon Ahmed, Syed Anees Ahmed, Karolina Akinosoglou, Mohammed Ahmed Akkaif, Salah Al Awaidy, Samer O Alalalmeh, Mohammed Albashtawy, Mohammad T AlBataineh, Adel Ali Saeed Al-Gheethi, Fadwa Naji Alhalaiqa, Robert Kaba Alhassan, Abid Ali, Liaqat Ali, Mohammed Usman Ali, Syed Shujait Ali, Waad Ali, Joseph Uy Almazan, Jaber S Alqahtani, Ahmad Alrawashdeh, Rami H Al-Rifai, Najim Z Alshahrani, Khaled Altartoor, Jaffar A Al-Tawfiq, Nelson Alvis-Guzman, Yaser Mohammed Al-Worafi, Hany Aly, Safwat Aly, Karem H Alzoubi, Walid Adnan Al-Zyoud, Abebe Feyissa Amhare, Hubert Amu, Ganiyu Adeniyi Amusa, Abhishek Anil, Saeid Anvari, Ekenedilichukwu Emmanuel Anyabolo, Jalal Arabloo, Mosab Arafat, Demelash Areda, Brhane Berhe Aregawi, Abdulfatai Aremu, Seyyed Shamsadin Athari, Avinash Aujayeb, Zewdu Bishaw Aynalem, Sina Azadnajafabad, Ahmed Y Azzam, Muhammad Badar, Pegah Bahrami Taghanaki, Saeed Bahramian, Atif Amin Baig, Milica Bajcetic, Senthilkumar Balakrishnan, Maciej Banach, Mainak Bardhan, Hiba Jawdat Barqawi, Mohammad-Mahdi Bastan, Kavita Batra, Ravi Batra, Amir Hossein Behnoush, Maryam Beiranvand, Alemu Gedefie Belete, Melaku Ashagrie Belete, Apostolos Beloukas, Azizullah Beran, Pankaj Bhardwaj, Ashish Bhargava, Ajay Nagesh Bhat, Mohiuddin Ahmed Bhuiyan, Veera R Bitra, Aadam Olalekan Bodunrin, Eyob Ketema Bogale, Sri Harsha Boppana, Hamed Borhany, Souad Bouaoud, Colin Stewart Brown, Danilo Buonsenso, Yasser Bustanji, Luis Alberto Cámera, Carlos A Castañeda-Orjuela, Luca Cegolon, Muthia Cenderadewi, Sandip Chakraborty, Vijay Kumar Chattu, Esther T W Cheng, Fatemeh Chichagi, Patrick R Ching, Hitesh Chopra, Sonali Gajanan Choudhari, Devasahayam J Christopher, Dinh-Toi Chu, Isaac Sunday Chukwu, Erin Chung, Alexandru Corlateanu, Natalia Cruz-Martins, Sriharsha Dadana, Omid Dadras, Tukur Dahiru, Xiaochen Dai, Jai K Das, Nihar Ranjan Dash, Mohsen Dashti, Mohadese Dashtkoohi, Fernando Pio De la Hoz, Shayom Debopadhaya, Berecha Hundessa Demessa, Asmamaw Bizuneh Demis, Vinoth Gnana Chellaiyan Devanbu, Devananda Devegowda, Kuldeep Dhama, Vishal R Dhulipala, Daniel Diaz, Michael J Diaz, Thanh Chi Do, Thao Huynh Phuong Do, Masoud Dodangeh, Fariba Dorostkar, Ashel Chelsea Dsouza, Haneil Larson Dsouza, Senbagam Duraisamy, Oyewole Christopher Durojaiye, Arkadiusz Marian Dziedzic, Abdelaziz Ed-Dra, Michael Ekholuenetale, Temitope Cyrus Ekundayo, Iman El Sayed, Faris El-Dahiyat, Muhammed Elhadi, Mohammed Elshaer, Majid Eslami, Ugochukwu Anthony Eze, Adeniyi Francis Fagbamigbe, Ali Faramarzi, Folorunso Oludayo Fasina, Nuno Ferreira, Florian Fischer, Ida Fitriana, Luisa S Flor, Santosh Gaihre, Márió Gajdács, Nasrin Galehdar, Mohammad Arfat Ganiyani, Miglas Welay Gebregergis, Mesfin Gebrehiwot, Teferi Gebru Gebremeskel, Genanew K Getahun, Molla Getie, Keyghobad Ghadiri, Afsaneh Ghasemzadeh, Mahsa Ghorbani, Mohamad Goldust, Mahaveer Golechha, Pouya Goleij, Giuseppe Gorini, Anmol Goyal, Shi-Yang Guan, Giovanni Guarducci, Mesay Dechasa Gudeta, Renu Gupta, Sapna Gupta, Veer Bala Gupta, Vivek Kumar Gupta, Mostafa Hadei, Najah R Hadi, Arvin Haj-Mirzaian, Rabih Halwani, Samer Hamidi, Ahmad Hammoud, Nasrin Hanifi, Fahad Hanna, Zaim Anan Haq, Md Rabiul Haque, S M Mahmudul Hasan, Hamidreza Hasani, Md Saquib Hasnain, Hadi Hassankhani, Johannes Haubold, Khezar Hayat, Omar E Hegazi, Kamal Hezam, Ramesh Holla, Praveen Hoogar, Nobuyuki Horita, Mihaela Hostiuc, Hong-Han Huynh, Segun Emmanuel Ibitoye, Olayinka Stephen Ilesanmi, Irena M Ilic, Milena D Ilic, Mohammad Tarique Imam, Mustafa Alhaji Isa, Md Rabiul Islam, Sheikh Mohammed Shariful Islam, Nahlah Elkudssiah Ismail, Masao Iwagami, Vinothini J, Abdollah Jafarzadeh, Khushleen Jaggi, Ammar Abdulrahman Jairoun, Mihajlo Jakovljevic, Elham Jamshidi, Shubha Jayaram, Bijay Mukesh Jeswani, Ravi Prakash Jha, Jobinse Jose, Nitin Joseph, Charity Ehimwenma Joshua, Jacek Jerzy Jozwiak, Vaishali K, Zubair Kabir, Himal Kandel, Kehinde Kazeem Kanmodi, Surya Kant, Rami S Kantar, Ibraheem M Karaye, Arman Karimi Behnagh, Navjot Kaur, Himanshu Khajuria, Amirmohammad Khalaji, Faham Khamesipour, Gulfaraz Khan, M Nuruzzaman Khan, Maseer Khan, Mohammad Jobair Khan, Min Seo Kim, Ruth W Kimokoti, Sonali Kochhar, Vladimir Andreevich Korshunov, Soewarta Kosen, Kewal Krishan, Hare Krishna, Vijay Krishnamoorthy, Barthelemy Kuate Defo, Md Abdul Kuddus, Mohammed Kuddus, Ilari Kuitunen, Mukhtar Kulimbet, Dewesh Kumar, Om P Kurmi, L V Simhachalam Kutikuppala, Chandrakant Lahariya, Dharmesh Kumar Lal, Savita Lasrado, Kaveh Latifinaibin, Huu-Hoai Le, Nhi Huu Hanh Le, Thao Thi Thu Le, Trang Diep Thanh Le, Seung Won Lee, Wei-Chen Lee, Ming-Chieh Li, Peng Li, Stephen S Lim, Gang Liu, Runben Liu, Wei Liu, Xiaofeng Liu, Xuefeng Liu, László Lorenzovici, Lisha Luo, Azeem Majeed, Elaheh Malakan Rad, Kashish Malhotra, Iram Malik, Aseer Manilal, Bharati Mehta, Tesfahun Mekene Meto, Mathewos M Mekonnen, Hadush Negash Meles, Ziad Ahmed Memish, Max Alberto Mendez-Lopez, Sultan Ayoub Meo, Mohsen Merati, Tomislav Mestrovic, Sachith Mettananda, Le Huu Nhat Minh, Erkin M Mirrakhimov, Arup Kumar Misra, Ahmed Ismail Mohamed, Nouh Saad Mohamed, Mesud Mohammed, Mustapha Mohammed, Ali H Mokdad, Lorenzo Monasta, Mohammad Ali Moni, AmirAli Moodi Ghalibaf, Catrin E Moore, Lidia Morawska, Rohith Motappa, Vincent Mougin, Parsa Mousavi, Ghulam Mustafa, Pirouz Naghavi, Ganesh R Naik, Firzan Nainu, Mohammad Sadeq Najafi, Soroush Najdaghi, Hastyar Hama Rashid Najmuldeen, Shumaila Nargus, Delaram Narimani Davani, Mohammad Naser, Zuhair S Natto, Biswa Prakash Nayak, Seyed Aria Nejadghaderi, Dang H Nguyen, Hau Thi Hien Nguyen, Van Thanh Nguyen, Taxiarchis Konstantinos Nikolouzakis, Efaq Ali Noman, Chisom Adaobi Nri-Ezedi, Virginia Nuñez-Samudio, Vincent Ebuka Nwatah, Ismail A Odetokun, Akinkunmi Paul Okekunle, Osaretin Christabel Okonji, Patrick Godwin Okwute, Titilope O Olanipekun, Isaac Iyinoluwa Olufadewa, Bolajoko Olubukunola Olusanya, Goran Latif Omer, Kenneth Ikenna Onyedibe, Michal Ordak, Verner N Orish, Esteban Ortiz-Prado, Nikita Otstavnov, Amel Ouyahia, Mahesh Padukudru P A, Jagadish Rao Padubidri, Ashok Pandey, Ioannis Pantazopoulos, Shahina Pardhan, Pragyan Paramita Parija, Romil R Parikh, Seoyeon Park, Ashwaghosha Parthasarathi, Maja Pasovic, Aslam Ramjan Pathan, Shankargouda Patil, Shrikant Pawar, Prince Peprah, Arokiasamy Perianayagam, Dhayaneethie Perumal, Ionela-Roxana Petcu, Hoang Nhat Pham, Hoang Tran Pham, Anil K Philip, David M Pigott, Zahra Zahid Piracha, Dimitri Poddighe, Roman V Polibin, Maarten J Postma, Reza Pourbabaki, Elton Junio Sady Prates, Jagadeesh Puvvula, Asma Saleem Qazi, Gangzhen Qian, Quinn Rafferty, Fakher Rahim, Mehran Rahimi, Vafa Rahimi-Movaghar, Md Obaidur Rahman, Mosiur Rahman, Muhammad Aziz Rahman, Mohammad Rahmanian, Nazanin Rahmanian, Vahid Rahmanian, Masoud Rahmati, Prashant Rajput, Mahmoud Mohammed Ramadan, Shakthi Kumaran Ramasamy, Pushkal Sinduvadi Ramesh, Indu Ramachandra Rao, Mithun Rao, Sowmya J Rao, Sina Rashedi, Mohammad-Mahdi Rashidi, Devarajan Rathish, Nakul Ravikumar, Salman Rawaf, Elrashdy Moustafa Mohamed Redwan, Luis Felipe Felipe Reyes, Nazila Rezaei, Nima Rezaei, Omid Rezahosseini, Syed Mohd Danish Rizvi, Jefferson Antonio Buendia Rodriguez, Luca Ronfani, Shekoufeh Roudashti, Priyanka Roy, Guilherme de Andrade Ruela, Basema Ahmad Saddik, Mohammad Reza Saeb, Umar Saeed, Pooya Saeedi, Mehdi Safari, Fatemeh Saheb Sharif-Askari, Narjes Saheb Sharif-Askari, Amirhossein Sahebkar, Monalisha Sahu, Joseph W Sakshaug, Nasir Salam, Afeez Abolarinwa Salami, Mohamed A Saleh, Malik Sallam, Yoseph Leonardo Samodra, Rama Krishna Sanjeev, Milena M Santric-Milicevic, Aswini Saravanan, Benn Sartorius, Anudeep Sathyanarayan, Jennifer Saulam, Sonia Saxena, Ganesh Kumar Saya, Benedikt Michael Schaarschmidt, Austin E Schumacher, Mansour Sedighi, Ashenafi Kibret Sendekie, Subramanian Senthilkumaran, Yashendra Sethi, SeyedAhmad SeyedAlinaghi, Mahan Shafie, Samiah Shahid, Masood Ali Shaikh, Sunder Sham, Mohammad Ali Shamshirgaran, Mohd Shanawaz, Mohammed Shannawaz, Amin Sharifan, Javad Sharifi-Rad, Rajesh P Shastry, Aziz Sheikh, Mika Shigematsu, Rahman Shiri, Aminu Shittu, Ivy Shiue, Seyed Afshin Shorofi, Emmanuel Edwar Siddig, Colin R Simpson, Jasvinder A Singh, Paramdeep Singh, Surjit Singh, Robert Sinto, Ranjan Solanki, Sameh S M Soliman, Muhammad Suleman, Rizwan Suliankatchi Abdulkader, Chandan Kumar Swain, Lukasz Szarpak, Seyyed Mohammad Tabatabaei, Mohammad Tabish, Zanan Mohammed-Ameen Taha, Jabeen Taiba, Iman M Talaat, Jacques Lukenze Tamuzi, Birhan Tsegaw Taye, Yibekal Manaye Tefera, Mohamad-Hani Temsah, Dufera Rikitu Terefa, Ramna Thakur, Rekha Thapar, Sathish Thirunavukkarasu, Ales Tichopad, Jansje Henny Vera Ticoalu, Marcos Roberto Tovani-Palone, Nghia Minh Tran, Ngoc Ha Tran, Nguyen Tran Minh Duc, Guesh Mebrahtom Tsegay, Munkhtuya Tumurkhuu, Aniefiok John Udoakang, Era Upadhyay, Seyed Mohammad Vahabi, Asokan Govindaraj Vaithinathan, Rohollah Valizadeh, Tommi Juhani Vasankari, Manish Vinayak, Muhammad Waqas, Haftom Legese Weldetinsaa, Nuwan Darshana Wickramasinghe, Ali Yadollahpour, Sajad Yaghoubi, Saber Yezli, Dehui Yin, Dong Keon Yon, Naohiro Yonemoto, Yong Yu, Fathiah Zakham, Ghazal G Z Zandieh, Iman Zare, Fatemeh Zarimeidani, Michael Zastrozhin, Chunxia Zhai, Haijun Zhang, Zhi-Jiang Zhang, Yang Zhao, Juexiao Zhou, Hafsa Zia, Magdalena Zielińska, Mohammad Zoladl, Samer H Zyoud, Aleksandr Y Aravkin, Nicholas J Kassebaum, Mohsen Naghavi, Theo Vos, Simon I Hay, Christopher J L Murray, Hmwe Hmwe Kyu

## Abstract

**Background:**

Upper respiratory infections (URIs) are the leading cause of acute disease incidence worldwide and contribute to a substantial health-care burden. Although acute otitis media is a common complication of URIs, the combined global burden of URIs and otitis media has not been studied comprehensively. We used results from the Global Burden of Diseases, Injuries, and Risk Factors Study 2021 to explore the fatal and non-fatal burden of the two diseases across all age groups, including a granular analysis of children younger than 5 years, in 204 countries and territories from 1990 to 2021.

**Methods:**

Mortality due to URIs and otitis media was estimated with use of vital registration and sample-based vital registration data, which are used as inputs to the Cause of Death Ensemble model to separately model URIs and otitis media mortality by age and sex. Morbidity was modelled with a Bayesian meta-regression tool using data from published studies identified via systematic reviews, population-based survey data, and cause-specific URI and otitis media mortality estimates. Additionally, we assessed and compared the burden of otitis media as it relates to URIs and examined the collective burden and contributing risk factors of both diseases.

**Findings:**

The global number of new episodes of URIs was 12·8 billion (95% uncertainty interval 11·4 to 14·5) for all ages across males and females in 2021. The global all-age incidence rate of URIs decreased by 10·1% (–12·0 to –8·1) from 1990 to 2019. From 2019 to 2021, the global all-age incidence rate fell by 0·5% (–0·8 to –0·1). Globally, the incidence rate of URIs was 162 484·8 per 100 000 population (144 834·0 to 183 289·4) in 2021, a decrease of 10·5% (–12·4 to –8·4) from 1990, when the incidence rate was 181 552·5 per 100 000 population (160 827·4 to 206 214·7). The highest incidence rates of URIs were seen in children younger than 2 years in 2021, and the largest number of episodes was in children aged 5–9 years. The number of new episodes of otitis media globally for all ages was 391 million (292 to 525) in 2021. The global incidence rate of otitis media was 4958·9 per 100 000 (3705·4 to 6658·6) in 2021, a decrease of 16·3% (–18·1 to –14·0) from 1990, when the incidence rate was 5925·5 per 100 000 (4371·8 to 8097·9). The incidence rate of otitis media in 2021 was highest in children younger than 2 years, and the largest number of episodes was in children aged 2–4 years. The mortality rate of URIs in 2021 was 0·2 per 100 000 (0·1 to 0·5), a decrease of 64·2% (–84·6 to –43·4) from 1990, when the mortality rate was 0·7 per 100 000 (0·2 to 1·1). In both 1990 and 2021, the mortality rate of otitis media was less than 0·1 per 100 000. Together, the combined burden accounted for by URIs and otitis media in 2021 was 6·86 million (4·24 to 10·4) years lived with disability and 8·16 million (4·99 to 12·0) disability-adjusted life-years (DALYs) for all ages across males and females. Globally, the all-age DALY rate of URIs and otitis media combined in 2021 was 103 per 100 000 (63 to 152). Infants aged 1–5 months had the highest combined DALY rate in 2021 (647 per 100 000 [189 to 1412]), followed by early neonates (aged 0–6 days; 582 per 100 000 [176 to 1297]) and late neonates (aged 7–24 days; 482 per 100 000 [161 to 1052]).

**Interpretation:**

The findings of this study highlight the widespread burden posed by URIs and otitis media across all age groups and both sexes. There is a continued need for surveillance, prevention, and management to better understand and reduce the burden associated with URIs and otitis media, and research is needed to assess their impacts on individuals, communities, economies, and health-care systems worldwide.

**Funding:**

Bill & Melinda Gates Foundation.

## Introduction

Upper respiratory infections (URIs) are the leading cause of acute disease incidence worldwide.^1^ Despite their relatively low risk of severe illness and mortality, URIs pose a substantial economic and health-care burden due to medical expenses, lost productivity, and increased health-system strain.^2^, ^3^, ^4^ This burden is particularly relevant for primary care providers when considering the impact of concurrent or subsequent otitis media, a common cause of care seeking for children younger than 5 years.^5^ Additionally, the mismanagement of URIs and otitis media contributes considerably to increased antimicrobial resistance globally, with URIs being a major contributor to antibiotic prescriptions.^6^, ^7^, ^8^, ^9^

**Research in context**

**Evidence before this study**

Previous research using data from the Global Burden of Diseases, Injuries, and Risk Factors Study (GBD) 2019 outlined the burden of upper respiratory infections (URIs) on an aggregate of children younger than 5 years, the age group in which URI burden is the highest. Studies have previously established a strong link between URIs and otitis media in adults and children, indicating that URIs can adversely affect the eustachian tube by facilitating pathogen colonisation of the middle ear. We searched PubMed for the terms (“upper respiratory infection*” OR “URI” OR “respiratory tract infections”) AND (“otitis media”) AND (“burden” OR “estimates” OR “prevalence” OR “incidence”) AND (“risk factor*”), with no language restrictions, for publications from Jan 1, 1980, to Oct 8, 2023. This search yielded 41 articles. Of these studies, 20 reported on URI incidence, ten reported on otitis media incidence, and 11 dealt with both URIs and otitis media. Of the 11 studies reporting on URIs and otitis media, all measured incidence in children younger than 8 years, with no granular age breakdowns. None of the 11 studies reported years lived with disability (YLDs), disability-adjusted life-years (DALYs), years of life lost, or mortality. We also did not find studies that reported the combined burden of URIs and otitis media. Of the 11 studies, nine reported on subnational trends and two were reviews that did not report global estimates.

**Added value of this study**

For the first time, this study assessed the incidence, mortality, YLDs, and DALYs from URIs in more granular age groups of children aged 1–5 months, 6–11 months, 12–23 months, and 2–4 years. We also integrated many new data sources on the morbidity and mortality of URIs since GBD 2019. Until now, the comprehensive burden of otitis media, a disease closely related to URIs, has not been assessed. This study shows the similarities in trends between these two conditions and provides new insights into the burden of otitis media, as well as the combined burden of both URIs and otitis media, by providing the incidence, mortality, YLDs, and DALYs of otitis media for 204 countries across all age groups, by sex.

**Implications of all the available evidence**

URIs result in substantial morbidity and economic burden worldwide. Although they rarely cause death or severe disease, URIs can lead to more serious infections such as otitis media and lower respiratory infections, as seen for SARS-CoV-2, and contribute to antimicrobial resistance through the inappropriate prescription of antibiotic medicines as a treatment for otitis media, URIs, or both. Understanding the interconnected nature of URIs and otitis media is crucial to addressing both the individual and combined burden of these diseases effectively through the design of strategies and public health interventions to address their impact. Strategies could include promoting better hygiene practices, implementing antimicrobial stewardship programmes, emphasising the importance of as well as ensuring equal access to vaccinations, and conducting further research to elucidate the underlying causes and contributing factors. Implementing targeted preventive interventions could help to reduce the burden of URIs and otitis media in children younger than 2 years, who account for the highest rate of episodes across all age groups. By comprehensively addressing the impact of URIs and otitis media, we can advance global public health efforts and enhance the quality of life of affected individuals and communities.

URIs can be acquired through various respiratory routes, ranging from the transmission of pathogens onto mucous membranes via contaminated hands to the inhalation of aerosols from an infected individual.^10^, ^11^ These transmission methods are influenced by factors such as ambient temperature, humidity, and crowding.^11^ URIs are caused by numerous pathogens, including rhinoviruses, coronaviruses, influenza, respiratory syncytial virus (RSV), *Streptococcus pyogenes, Streptococcus pneumoniae, Haemophilus influenzae*, and *Mycoplasma pneumoniae.* The potential risk of URIs progressing into more severe disease is substantial given the sheer magnitude of URI cases each year. This potential risk is particularly important in the wake of the COVID-19 pandemic, which often saw infection beginning in the upper respiratory tract before progressing to more life-threatening illness.^12^, ^13^

Acute otitis media, an infection of the middle ear that can be caused by bacteria, viruses, or a combination of both, is the second most common paediatric illness following URIs.^14^, ^15^ Acute otitis media is characterised by the presence of fluid within the middle ear alongside the signs of acute infection and ear pain.^16^ Acute otitis media is often a complication following a URI when the eustachian tube is colonised by URI-associated pathogens through congestion of the nasal and nasopharyngeal mucosa, allowing pathogens to enter the middle ear and trigger inflammation.^17^ In previous research, more than 60% of URI episodes in children younger than 35 months were complicated by a simultaneous case of otitis media.^18^ Infants who frequently have URIs are more likely to contract a concurrent or subsequent instance of otitis media.^19^

Although the link between URIs and acute otitis media is well established, a comprehensive assessment of their combined burden across age groups and diverse geographical contexts has been lacking, particularly in children younger than 5 years, thereby limiting informed decision making on interventions and policies to address the burden of these two associated diseases. Using the estimates produced by the Global Burden of Diseases, Injuries, and Risk Factors Study (GBD) 2021, we aimed to assess the incidence, mortality, years lived with disability (YLDs), and disability-adjusted life-years (DALYs) of URIs and otitis media across age groups and 204 countries and territories. Compared with the previous iteration, GBD 2019, we assessed the burden in more granular age groups of children younger than 5 years (1–5 months, 6–11 months, 12–23 months, and 2–4 years) to better understand the burden in children, who have the highest incidence rates of URIs and otitis media. We also aimed to analyse the combined burden of URIs and otitis media through YLDs, reflecting their interconnected nature.

## Methods

### GBD modelling overview

GBD 2021 produced estimates of deaths, incidence, years of life lost (YLLs), YLDs, and DALYs for URIs and otitis media by age and sex for 204 countries and territories from 1990 to 2021. Modelling was done using 1000 draws, and 95% uncertainty intervals (UIs) were calculated as the 25th and 975th ranked values of the 1000 draws. Percentage changes were calculated as the difference between the final value (for the year 2021) and the initial value (for the year 1990), then divided by the initial value and multiplied by 100. Age-standardised rates were computed using the GBD 2021 global population age standard. Socio-demographic Index (SDI) for each country was computed as the geometric mean of each country's lag-distributed income per capita, average years of schooling, and the total fertility rate in females younger than 25 years.^20^ Full descriptions of the GBD studies and methodology have been previously published.^21^

The GBD Sources tool, found on the Global Health Data Exchange (GHDx), provides all metadata for input sources described below. It allows readers to identify which sources were used for estimating an outcome in any given location. Statistical code used for GBD estimation is also publicly available on the GHDx. This research complies with the GATHER statement (appendix 1 p 77).

### Estimating incidence, prevalence, and mortality for URIs and otitis media

Incidence and prevalence of non-COVID-19 URIs were modelled using a Bayesian meta-regression tool, DisMod-MR 2.1.^21^ The inputs into the URI model consisted of data from published studies identified through systematic reviews and nationally representative surveys, including the US National Health Interview Surveys and Demographic and Health Surveys (DHS). Excluded from DisMod-MR 2.1 were data that were not population-based and studies that did not provide primary data, had a sample size of less than 150, or were reviews or case series. A more comprehensive description of the input data and the associated search string from the systematic review is provided in appendix 1 (pp 4–11).

In the GBD framework, acute and chronic otitis media are modelled as separate non-fatal health outcomes using DisMod-MR 2.1. The inputs into the otitis media models consisted of data from published studies identified via systematic reviews, population-based surveys, and health insurance claims data (more details can be found in appendix 1 p 11). For the purposes of this Article, we present the combination of acute and chronic non-fatal otitis media as one disease category, non-fatal otitis media, for consistency with fatal otitis media, which is modelled as a single entity.

The Cause of Death Ensemble model (CODEm) framework was used to estimate mortality due to non-COVID-19 URIs and otitis media separately in the GBD 2021 study, using data from vital registration, sample-based vital registration, and minimally invasive tissue sample diagnoses as the inputs.^21^ CODEm creates a diverse array of submodels with different functional forms (linear mixed-effects models with random intercepts at the super-region, region, and country levels, assuming these random effects are normally distributed, and spatiotemporal Gaussian process regression models) for the outcome variable, either the mortality rate or cause fraction, using various combinations of predictive covariates. Appendix 1 provides the complete list of covariates for URIs (p 4) and otitis media (p 11). The selection of the ensemble of models was based on the best performance in out-of-sample predictive validity tests.

### Estimating YLLs, YLDs, and DALYs

GBD calculates YLLs as the sum of each death multiplied by the standard life expectancy at each age. URIs have two severity levels: mild and moderate–severe URIs. YLDs from URIs were calculated by multiplying a disability weight for each of the URI severity levels and the percentage of episodes that fall into each level. Otitis media has ten severity levels: acute otitis media, severe infectious complications due to chronic otitis media, mild hearing loss due to chronic otitis media, moderate hearing loss due to chronic otitis media, mild hearing loss with ringing due to chronic otitis media, moderate hearing loss with ringing due to chronic otitis media, vertigo with mild hearing loss due to chronic otitis media, vertigo with mild hearing loss and ringing due to chronic otitis media, vertigo with moderate hearing loss due to chronic otitis media, and vertigo with moderate hearing loss and ringing due to chronic otitis media. YLDs from otitis media were calculated in the same manner as URIs by multiplying each severity level's corresponding disability weight with the percentage of episodes that are attributed to each severity level. The disability weights for severity levels of URIs and otitis media were derived from the GBD disability weights study^22^ and can be found in appendix 1 (pp 15–16). DALYs were calculated as the sum of YLLs and YLDs for all locations, years, and age groups, by sex. More detailed information on the calculation of YLLs, YLDs, and DALYs has been published elsewhere.^21^

### Risk attribution estimation for DALYs and YLDs

Detailed methods for GBD risk factor estimation have been published elsewhere.^23^ To summarise, we selected risk–outcome pairs that held a convincing or probable causal relationship between the risk factor and the outcome (URIs and household air pollution, as an example). Relative risks for the associations between the risk factors and URIs and otitis media were estimated on the basis of published systematic reviews. The level of exposure to risk factors was estimated using spatiotemporal Gaussian process regression, a Bayesian meta-regression method (DisMod-MR 2.1), or alternative methodology (appendix 1 pp 16–79). Using available data sources, exposure levels equating to minimum risk (theoretical minimum risk) were determined (appendix 1 pp 16–79). YLDs and DALYs attributed to each risk factor were computed by multiplying population attributable fractions by the relevant outcome quantity for year, age group, and sex.

More detailed URI and otitis media burden results by age and sex across locations and years are available in the GBD Results Tool.

### Role of the funding source

The funders of the study had no role in study design, data collection, data analysis, data interpretation, or writing of the report.

## Results

### Non-fatal and fatal burden of URIs

**Figure 1:**
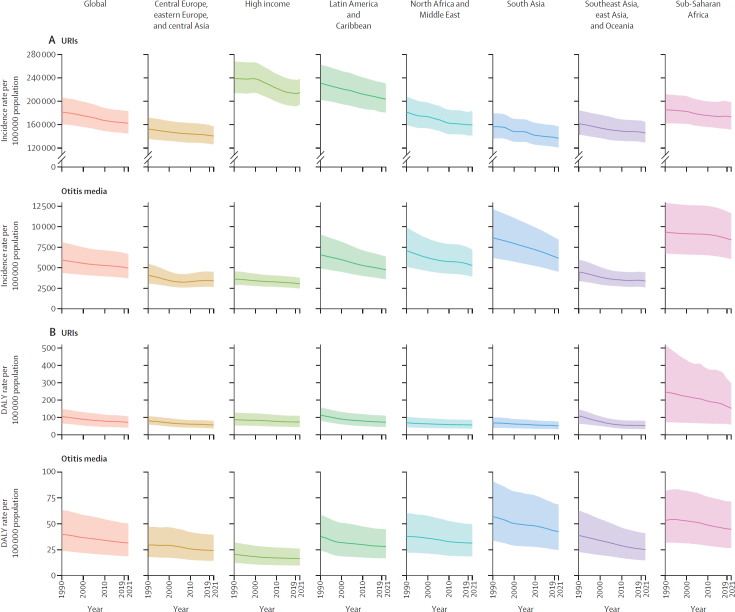
URI and otitis media incidence rates (A) and DALY rates (B) per 100 000 population, by super-region, in 1990 and 2021 Shaded areas are 95% uncertainty intervals. DALY=disability-adjusted life-year. URI=upper respiratory infection.

**Figure 2:**
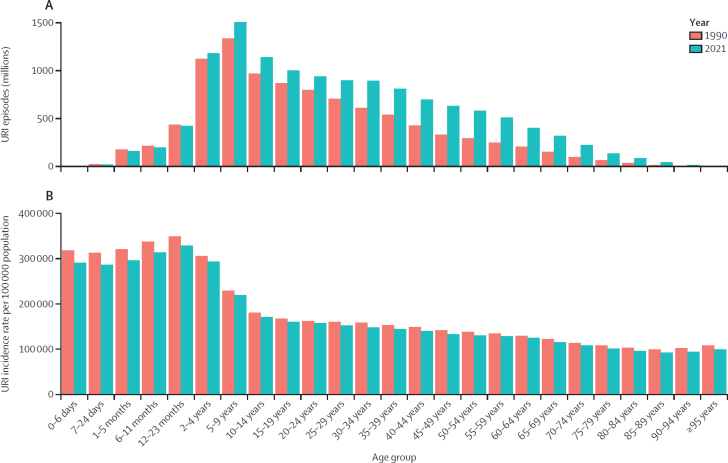
Global URI episodes in millions (A) and incidence rates per 100 000 population (B), by age, in 1990 and 2021 URI=upper respiratory infection.

In 2021, there were 12·8 billion (95% UI 11·4–14·5) episodes of URI globally for all ages across males and females (appendix 2 p 5). In 1990, this value was 9·68 billion (8·58–11·0). The all-age incidence rate of URI in 2021 was 162 484·8 per 100 000 population (144 834·0–183 289·4; appendix 2 p 5; figure 1). The highest incidence rate of URIs was seen in children aged 12–23 months (328 644·6 per 100 000 [249 094·0–420 845·1]), followed by those aged 6–11 months (313 772·9 per 100 000 [241 920·5–400 104·3]) and 1–5 months (295 690·7 [237 157·2–359 466·6]; appendix 2 p 5; figure 2). The largest count of URI episodes was seen in children aged 5–9 years (1·50 billion [1·02–2·06]), followed by those aged 2–4 years (1·18 billion [0·857–1·54]) and 10–14 years (1·14 billion [0·793–1·57]; appendix 2 p 5; figure 2). Among people aged 15 years and older, the age group with the largest incidence rate per 100 000 in 2021 was age 15–19 years (160 574·2 [111 176·9–216 450·9]), followed by 20–24 years (157 525·4 [112 631·9–214 581·1]), and 25–29 years (152 689·1 [110 765·7–207 683·7]). The same pattern was observed for URI episodes (appendix 2 p 5; figure 2).

Between 1990 and 2019, the global all-age incidence rate of URIs decreased by 10·1% (95% UI –12·0 to –8·1), from 181 552·5 per 100 000 (160 827·4 to 206 214·7) to 163 255·1 per 100 000 (145 630·6 to 183 924·0; figure 1; appendix 2 p 5). From 2019 to 2021, the global all-age incidence rate decreased by 0·5% (–0·8 to –0·1), to 162 484·8 per 100 000 (144 834·0 to 183 289·4), with substantial variation across regions, showing increasing, decreasing, or no changes in URI incidence rates (appendix 2 p 5).

The highest all-age incidence rates per 100 000 population in 2021 were seen in high-income North America (269 253·0 [95% UI 241 387·5–297 109·8]), Oceania (236 110·1 [208 105·9–267 109·9]), and Tropical Latin America (227 863·0 [201 295·0–259 060·5]; appendix 2 p 5; figure 1). When classified by SDI, the largest number of episodes in 2021 was seen in middle SDI regions at 3·85 billion (3·42–4·37) episodes, although the highest rate of URIs was held by high SDI regions (203 538·2 per 100 000 [183 388·8–225 459·6]; appendix 2 p 5).

Globally, URIs accounted for 19 600 deaths (95% UI 7040–41 600) in 2021 (appendix 2 p 6), with a mortality rate of 0·2 per 100 000 (0·1–0·5), a 64·2% (43·4–84·6) decrease in mortality rate from 1990 (appendix 2 p 6). The age distribution of URI deaths and mortality rates is illustrated in appendix 2 (p 2). The regions with the highest mortality rate per 100 000 in 2021 were eastern sub-Saharan Africa (1·4 per 100 000 [0·1–3·7]), western sub-Saharan Africa (1·3 per 100 000 [0·2–3·7]), and central sub-Saharan Africa (1·0 per 100 000 [0·1–3·3]; appendix 2 p 6). The highest number of deaths came from the low SDI regions in 2021 (12 100 [1100–31 500]), and the highest mortality rate was also from low SDI regions (1·1 per 100 000 [0·1–2·8]; appendix 2 p 6).

The number of YLDs from URIs in 2021 was 4·41 million (95% UI 2·68 to 6·73) globally (appendix 2 p 7). In 1990, the number of YLDs from URIs was 3·34 million (2·01 to 5·10), representing a 31·8% (29·1 to 34·9) increase from 1990 to 2021. In 2021, global YLLs from URIs reached 1·27 million (0·299 to 2·98), representing a decrease of 43·7% (–77·1 to –10·0) from 1990 (2·26 million [0·727 to 3·95]; appendix 2 p 7). In 2021, the number of global DALYs from URIs was 5·68 million (3·26 to 8·38), an increase of 1·3% (–15·4 to 19·3) from 1990, when DALYs totalled 5·60 million (3·45 to 7·91; appendix 2 p 7). The rate of DALYs per 100 000 has been decreasing across all super-regions (figure 1; appendix 2 p 9), with a global decline from 105·1 (64·6 to 148·3) in 1990, to 78·8 (45·2 to 116·0) in 2010, to 73·8 (42·3 to 109·3) in 2019, and to 72·0 (41·3 to 106·2) in 2021.

### Non-fatal and fatal burden of otitis media

**Figure 3:**
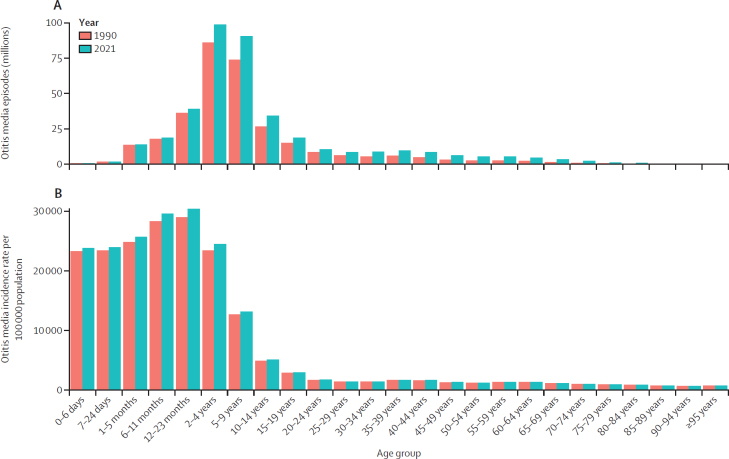
Global otitis media episodes in millions (A) and incidence rates per 100 000 population (B), by age, in 1990 and 2021

The number of episodes of otitis media globally for all ages across males and females reached 391 million (95% UI 292 to 525) in 2021, an increase from 316 million (233 to 432) in 1990 (appendix 2 p 10). The incidence rate of otitis media in 2021 was 4958·9 per 100 000 (3705·4 to 6658·6), a decrease of 16·3% (–18·1 to –14·0) since 1990 (5925·5 per 100 000 [4371·8 to 8097·9]; figure 1). The highest incidence rate was seen in children aged 12–23 months (30 404·7 per 100 000 [16 470·1 to 50 221·0]), followed by those aged 6–11 months (29 600·1 per 100 000 [17 571·3 to 45 019·3]) and 1–5 months (25 668·4 per 100 000 [15 768·7 to 38 629·4]; appendix 2 p 10; figure 3). The largest count of otitis media episodes was seen in children aged 2–4 years (98·6 million [55·2 to 155·0]), followed by those aged 5–9 years (90·6 million [45·5 to 159·0]) and 12–23 months (39·0 million [21·1 to 64·5]; figure 3). Among people aged 15 years and older, the largest number of episodes in 2021 was seen in those aged 15–19 years (18·6 million [11·4 to 29·1]), followed by those aged 20–24 years (10·5 million [4·84 to 18·5]), and 35–39 years (9·68 million [5·58 to 16·1]; appendix 2 p 10). The same pattern was observed for the incidence rate (appendix 2 p 10; figure 3).

The highest incidence rate of otitis media in 2021 was seen in western sub-Saharan Africa (8665·1 per 100 000 [95% UI 6267·5–12 058·9]; appendix 2 p 10; figure 1). The largest number of episodes came from low-middle SDI regions in 2021 (119 million [86·9–162]; appendix 2 p 10). Low SDI regions also had the highest rate of otitis media in 2021 (8244·0 per 100 000 [5944·4–11 412·5]; appendix 2 p 10).

Globally, the number of deaths caused by otitis media was 536 (95% UI 217 to 1240) in 2021 (appendix 2 p 11). In both 1990 and 2021, the mortality rate of otitis media was less than 0·1 per 100 000, with a decrease of 75·4% (–83·8 to –57·1) in this period (appendix 2 p 11). The age distribution of otitis media deaths and mortality rates is illustrated in appendix 2 (p 3).

The number of YLDs from otitis media in 2021 was 2·45 million (95% UI 1·43 to 3·94) globally (appendix 2 p 7). In 1990, otitis media YLDs reached 2·03 million (1·18 to 3·28), representing an increase of 20·7% (16·8 to 24·5) from 1990 to 2021 (appendix 2 p 7). In 2021, global all-age YLLs from otitis media reached 28 200 (9000 to 76 400), representing a decrease of 69·9% (–84·2 to –39·3) since 1990 (93 600 [52 000 to 168 000]; appendix 2 p 7). In 2021, the global number of otitis media DALYs was 2·48 million (1·46 to 3·97; appendix 2 p 7). The rate of DALYs per 100 000 has been decreasing across all super-regions, with a global decline from 39·8 (23·9 to 63·2) in 1990, to 34·0 (20·1 to 54·3) in 2010, to 31·7 (18·7 to 50·6) in 2019, and to 31·4 (18·5 to 50·4) in 2021 (figure 1; appendix 2 p 9).

### Combined burden of URIs and otitis media

The number of YLDs accounted for by URIs and otitis media together in 2021 was 6·86 million (95% UI 4·24–10·4) for all ages across males and females (appendix 2 p 13). The age group with the largest combined count of YLDs was age 5–9 years (925 000 [553 000–1 470 000]), followed by 10–14 years (697 000 [396 000–1 110 000]) and 2–4 years (661 000 [380 000–1 040 000]; appendix 2 p 13). Among people aged 15 years and older, the largest number of combined YLDs was seen in those aged 15–19 years (603 000 [351 000–973 000]), followed by those aged 20–24 years (521 000 [304 000–837 000]), and 25–29 years (459 000 [263 000–733 000]; appendix 2 p 13).

**Figure 4:**
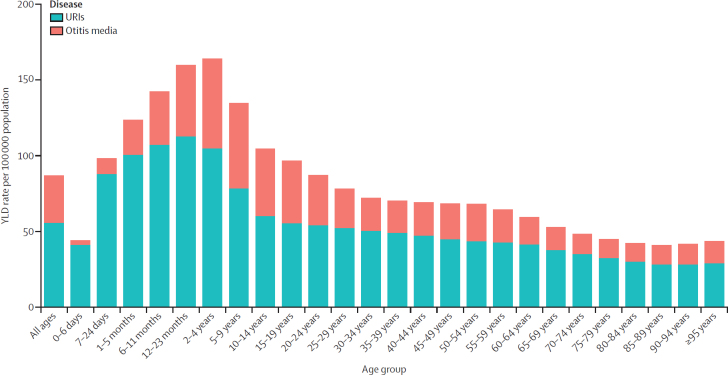
Combined YLD rates of URIs and otitis media globally, by age, in 2021 URIs=upper respiratory infections. YLD=years lived with disability.

The total combined rate of YLDs for all ages across males and females was 87 per 100 000 (54–132; figure 4; appendix 2 p 13). The age group with the largest combined rate of YLDs was age 2–4 years (164 per 100 000 [94–259]), followed by 12–23 months (160 per 100 000 [92–250]) and 6–11 months (142 per 100 000 [82–221]; figure 4; appendix 2 p 13). Among people aged 15 years or older, the largest combined rate was seen in those aged 15–19 years (97 per 100 000 [56–156]), followed by those aged 20–24 years (87 per 100 000 [51–140]), and 25–29 years (78 per 100 000 [45–125]; appendix 2 p 13).

The number of DALYs in 2021 from the combined burden of URIs and otitis media was 8·16 million (95% UI 4·99–12·0) for all ages across males and females (appendix 2 p 13). The age group with the largest count of DALYs was age 5–9 years (998 000 [596 000–1 570 000]), followed by 2–4 years (827 000 [474 000–1 240 000]) and 10–14 years (728 000 [421 000–1 130 000]; appendix 2 p 13). Among people aged 15 years and older, the largest count of DALYs was seen in those aged 15–19 years (622 000 [359 000–990 000]), followed by those aged 20–24 years (538 000 [318 000–853 000]), and 25–29 years (473 000 [278 000–750 000]; appendix 2 p 13).

The all-age combined DALY rate in 2021 was 103 per 100 000 (63–152; appendix 2 p 13). Infants in the 1–5 months age group had the highest DALY rate per 100 000 in 2021 (647 [189–1412]), followed by early neonates (aged 0–6 days; 582 per 100 000 [176–1297]) and late neonates (aged 7–24 days; 482 per 100 000 [161–1052]; appendix 2 p 13). Among people aged 15 years and older, the largest rate of DALYs was seen in those aged 15–19 years (100 per 100 000 [58–159]), followed by those aged 20–24 years (90 per 100 000 [53–143]), and 95 years and older (82 per 100 000 [57–127]; appendix 2 p 13).

**Figure 5:**
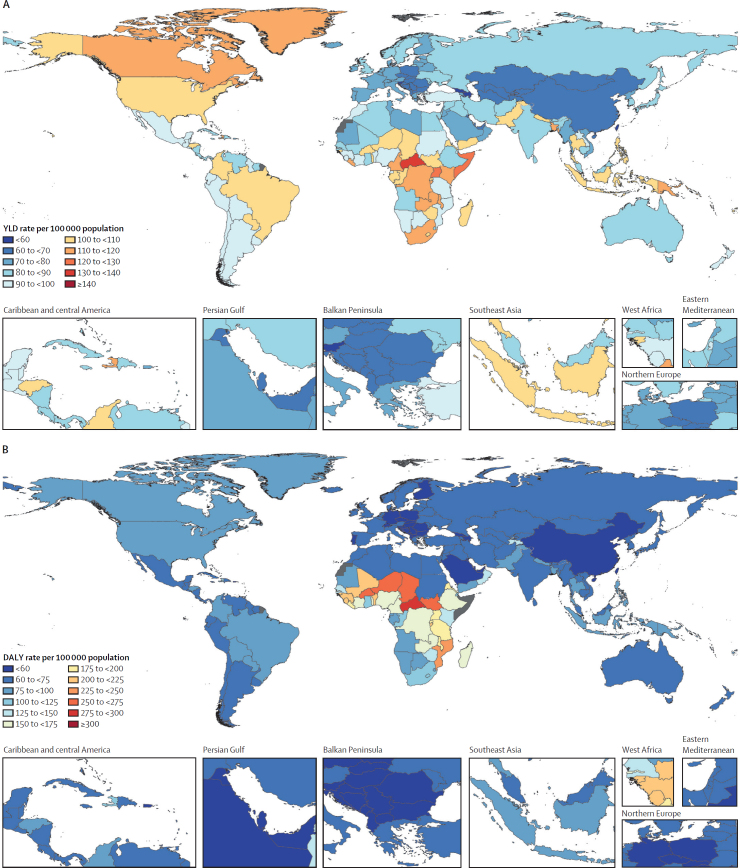
Maps of combined YLD rates (A) and DALY rates (B) for upper respiratory infections and otitis media in 2021 DALY=disability-adjusted life-year. YLD=years lived with disability.

The region with the largest combined DALY rate in 2021 was eastern sub-Saharan Africa (214·8 per 100 000 [95% UI 86·5–401·6]), followed by western sub-Saharan Africa (198·5 per 100 000 [86·8–395·7]) and central sub-Saharan Africa (178·4 per 100 000 [89·0–348·5]; appendix 2 p 14). The country with the highest combined DALY rate in 2021 was Somalia (364·3 per 100 000 [103·0–1108·1]), followed by Central African Republic (309·1 per 100 000 [119·3–684·1]) and Burkina Faso (286·4 per 100 000 [96·0–816·8]; figure 5B; appendix 2 p 14).

### Risk factor attribution of DALYs for URIs and otitis media

The total number of DALYs attributable to the evaluated risk factors of URIs in 2021 was 32 600 (95% UI 7690 to 79 100), a decrease of 54·6% (–79·7 to –29·0) since 1990, when the total was 71 800 (21 100 to 141 000; appendix 2 p 15). For otitis media, the total number of DALYs attributable to evaluated risk factors in 2021 was 80 200 (35 100 to 144 000), a decrease of 23·7% (–31·8 to –19·0) since 1990, when DALYs attributable to the evaluated risk factors reached 105 000 (48 600 to 183 000; appendix 2 p 15).

In the GBD framework, URI burden is attributable to four risk factors. The largest risk factor contributing to URI DALYs in 1990 was low birthweight (57 600 DALYs [95% UI 16 000 to 115 000]; appendix 2 p 15). Low birthweight continued to be the largest risk factor for URI DALYs in 2021 at 26 500 (5200 to 65 800), which was a decrease of 54·0% (–80·0 to –26·8) since 1990 (appendix 2 p 15). The second largest risk factor for URI DALYs in 2021 was short gestation (11 600 [1840 to 29 600]), followed by household air pollution (9200 [1620 to 22 800]) and ambient particulate matter (2510 [983 to 5800]; appendix 2 p 15).

Otitis media is attributable to five risk factors in the GBD framework. The greatest risk factor contributing to the DALYs of otitis media in 1990 was second-hand smoke (101 000 DALYs [95% UI 46 000 to 178 000]; appendix 2 p 15). In 2021, second-hand smoke remained the greatest risk contributing to otitis media DALYs at 79 600 DALYs (34 600 to 143 000), a decrease of 21·6% (–26·5 to –17·9) from 1990 (appendix 2 p 15). The second largest risk factor of otitis media DALYs in 2021 was low birthweight (444 [164 to 1070]), followed by household air pollution (250 [110 to 521]), short gestation (202 [69 to 486]), and ambient particulate matter (78 [36 to 144]; appendix 2 p 15).

## Discussion

To our knowledge, this study represents the first comprehensive assessment of the global burden of two interconnected infectious diseases, URIs and otitis media, highlighting the magnitude of these conditions and their impact on public health. The study also is the first to explore the burden of these diseases at more granular age and geographical levels. In 2021, there were an estimated 12·8 billion episodes of URIs and 19 600 deaths due to URIs, while otitis media accounted for 391 million episodes and 536 deaths. In both URIs and otitis media, the highest burden was seen in neonatal and paediatric age groups.

Our results show that URIs are widespread and associated with substantial morbidity. The incidence rate alone makes URIs the highest-ranking communicable disease across the infections studied in the GBD framework. Diarrhoea, the second highest-ranking communicable disease in 2021, accounted for approximately 59 000 episodes per 100 000 population, while the third highest-ranking communicable disease for all ages was COVID-19, estimated at 29 000 episodes per 100 000 population; both occurred at substantially lower rates than URIs (162 000 episodes per 100 000). In children younger than 5 years, the incidence rate of URIs was over three times greater than that of diarrhoea, and over 15 times greater than that of COVID-19.^24^

Although URIs rarely result in death, with 19 600 deaths globally in 2021, they contribute to nearly half of incident episodes of all diseases globally and impose a substantial burden on individuals, health-care systems, and economies.^2^, ^3^, ^4^, ^5^, ^21^ Of deaths from URIs, the highest mortality rates occurred among adults aged 95 years and older and newborn babies, showing the disproportionate impact of URIs among the more vulnerable members of the population. Mortality rates due to URIs were highest in sub-Saharan Africa, and the combined DALY rate followed the same trend, with highest rates in Somalia, Central African Republic, and Burkina Faso. This trend might be explained in part by lower access to quality health care in those regions. In 2019, universal health-care coverage in Somalia and Central African Republic largely lagged behind that in other countries.^25^ By improving universal health-care coverage, the mortality rates of URIs and other communicable diseases might be alleviated.

Despite the relatively low mortality rate, it is important to note that URIs can lead to more severe lower respiratory infections (LRIs), which are associated with much higher mortality rates.^26^ For example, SARS-CoV-2 often infected the upper respiratory tract before progressing to more severe and fatal disease in the lower respiratory tract.^27^ URIs can be caused by a variety of different viruses, including rhinoviruses, coronaviruses, adenoviruses, enteroviruses, influenza viruses, parainfluenza viruses, and RSV. Of these, rhinoviruses are among the predominant viral pathogens responsible for URIs.^28^ During the COVID-19 pandemic, rhinoviruses continued to propagate despite the use of face masks, physical distancing, and lockdown measures.^29^

In terms of URI-related morbidity, YLDs due to URIs are higher than those for many more prominent infectious diseases, such as tuberculosis, HIV, and LRIs.^21^ When considered in combination with otitis media, the impact of URIs in terms of healthy life lost due to disability grows, emphasising the long-term consequences and potential impact on quality of life caused by these infections. Similar to URIs, the incidence of otitis media has been increasing since 1990. Previous studies have consistently reported the highest incidence rates of otitis media in children aged 1–4 years, with a peak incidence in children aged 6–18 months.^18^, ^21^, ^30^ In this study, the highest incidence rates of otitis media were estimated for children aged 12–23 months, followed by those aged 6–11 months, showing considerable overlap with age groups most affected by URIs. Together, URIs and otitis media accounted for 6·86 million YLDs in 2021, with children aged 5–9 years contributing the largest proportion of this burden, followed by those aged 10–14 years. These infections spread quickly among school-aged children,^11^, ^16^, ^18^, ^19^ underscoring the need for targeted prevention and intervention strategies, which could include promoting appropriate hygiene practices, implementing antimicrobial stewardship programmes, and emphasising the importance of vaccinations.^7^, ^16^, ^31^, ^32^, ^33^, ^34^, ^35^

The rise in antimicrobial resistance (AMR) is particularly notable when considering the widespread occurrence of URIs and otitis media, as URIs account for a large proportion of antibiotic prescriptions in primary care settings.^36^ The inappropriate prescription of antibiotics in some cases of URI facilitates an environment conducive to the emergence of AMR, especially as most URIs are self-limiting and require only symptomatic relief.^36^ Further understanding of the causes of URIs and their associated pathogens is needed to enable better preparedness for outbreaks and the development of effective prevention and control strategies that do not include the use of antibiotic treatments.^37^

Recent advancements in our understanding of AMR underscore the evolving challenges in treating otitis media globally.^38^ These findings are particularly relevant given the high dependency of otitis media treatment on effective antibiotics. Pneumococcal conjugate vaccines in particular have been shown to have a positive impact on decreasing otitis media,^39^ but recent studies have reported a shift in the bacteria causing otitis media towards pneumococcal types not included in vaccines and other bacteria.^40^ This shift, coupled with regional variations in AMR, suggest the need for ongoing surveillance, tailored treatment and prevention strategies, and stewardship interventions to reduce unnecessary antibiotic use.

In addition to the significant potential for antibiotic overuse, URIs and otitis media are associated with increased health expenses and substantial health-related productivity losses, particularly due to their high prevalence among children, which contributes to work absenteeism among parents and guardians. Parents or guardians are often faced with the difficult decision of sending sick children to school or missing work,^41^ a choice that is disproportionately difficult for low-income families and that potentially has a broader impact on children and communities than could be examined in the scope of this study.

The heavy burden of URIs and otitis media presents opportunities for the development of vaccines and medications that can reduce the burden of these diseases and their impact on society. Such development is particularly important when considering the ineffectiveness of common over-the-counter cold medicines.^42^ Some preventive options for otitis media include published guidelines for prevention and intervention such as those from the Centers for Disease Control and Prevention,^43^ continued medical education to both medical practitioners and the general public on the pathophysiology of otitis media as well as its linkage to URIs, and the introduction of relevant vaccines, such as the higher-valency pneumococcal conjugate vaccines that are currently becoming available, into national immunisation programmes.^44^, ^45^ Additionally, developments in mucosal immunology and genetics are facilitating more targeted treatment strategies, potentially transforming the management of URIs and otitis media in the near future.^46^, ^47^, ^48^

Ultimately, a multifaceted approach might be needed to help prevent and manage the combined impact of URIs and otitis media, given the varied effectiveness of public health interventions.^49^, ^50^ Unlike diseases such as measles, for which vaccination is a straightforward solution, strategies for URIs and otitis media must be more diverse and specific. Tailoring public health initiatives to local needs and resources is essential, and it is important to understand local URI and otitis media burdens and corresponding risk factors. For instance, low birthweight, a leading risk factor for URIs and otitis media in our study, can be reduced through improved maternal health programmes and comprehensive care during childhood, especially in low-income areas.^51^, ^52^ While the prevalence of low birthweight has slightly declined globally since the 2000s,^53^ it remains a considerable risk factor for both diseases. Similarly, exposure to household air pollution from solid fuels—another leading risk factor for both URIs and otitis media in our study—has decreased in some regions since the 1990s but continues to be a major problem, particularly in low-income and middle-income countries, with 2·3 billion people globally still having no access to clean cooking as of 2021.^54^ Addressing the leading risk factors for URIs and otitis media, including low birthweight and exposure to household air pollution, as well as exposure to second-hand smoke,^55^ the leading risk factor for otitis media, could help reduce the burden of these diseases and other respiratory conditions.

This study has several limitations, including the availability of URI and otitis media data. In data-scarce locations, estimates were generated on the basis of regional patterns, covariates, and out-of-sample predictive validity assessment. Locations with few or no data produce wide uncertainty intervals during the estimation process. Even in situations where data were abundant, measurements might not have been based on the same case definitions across studies. To combat these limitations, methods of standardising case definitions have provided more robust analyses.^21^ With the delays that occur in data reporting, the recency of included data varies across the two diseases. For example, URIs had non-fatal data up to 2021, but the most recent non-fatal data for otitis media included in the modelling were from 2018. Data can also be problematic in situations where one disease can progress into another more severe disease; the survey and literature data used in this study do not specify when a case of URI has progressed into a case of LRI. Additionally, population attributable fraction assumptions imply that there is a causal link between exposure and outcome without confounding, that removing the exposure would not affect the distribution of unrelated risk factors, and that a practical intervention to eliminate the exposure is possible.^56^ However, these assumptions might not always hold true in real-world scenarios. Furthermore, we attempted to account for potential biases when quantifying the relationship between risk factors and outcomes by incorporating bias covariates; however, these might not fully identify and correct bias if the input studies have inherent biases. Finally, the risk factors evaluated for both URIs and otitis media in our study might not encompass all possible risk factors, as our inclusion criteria were limited to risk–outcome pairs with convincing or probable evidence of a causal relationship, in line with GBD standards.

While SARS-CoV-2 and other pathogens probably contribute to the global burden of URIs, the exact proportion remains unclear due to the infrequent and variable use of comprehensive diagnostic testing and lack of robust surveillance for URIs at the pathogen level.^57^, ^58^ This limits our understanding of the distribution and frequency of URI pathogens and hinders the potential for more targeted, early interventions. The evolving landscape of respiratory pathogens necessitates further research and more widespread pathogen surveillance to clarify the relative contributions of various pathogens to the global burden of URIs and inform targeted public health interventions.^59^, ^60^ Currently, GBD does not quantify pathogens for URIs and otitis media. Pathogen burden is crucial in understanding the connection between URIs and more severe disease progression, as well as in the creation of targeted disease-mitigating protocols, including vaccines. To address this limitation, we aim to include detailed results on pathogen burden in future rounds of GBD. Additionally, we will be able to quantify the indirect impact of the COVID-19 pandemic on the burden of URIs and otitis media in subsequent rounds of the GBD as more data become available. At present, assessing the available data, which are limited, the impact of COVID-19 on URI episodes is unclear. DHS data from five countries with at least three timepoints including 2020 or 2021 (available as of June, 2024) do not show a clear pattern of effect from the COVID-19 pandemic on URIs. Some countries have shown an increase in URI prevalence from prepandemic years to postpandemic years, while others have shown a decrease. In Madagascar, for example, there was an increase in period prevalence from 2009 (5·74% [5·14–6·33]) to 2021 (12·5% [11·7–13·4]), whereas Côte d'Ivoire showed a decrease in period prevalence from 2012 (10·7% [9·61871–11·82528]) to 2021 (9·48% [8·52–10·4]; appendix 2 p 4). In those countries that showed a decrease, the trend of declining URI prevalence was already evident before the pandemic. Therefore, it remains unclear whether the decrease can be attributed to non-pharmaceutical interventions or is merely a continuation of previous trends. For DHS countries that showed an increase between 2019 and 2021, it is possible that changes in DHS data are due to misclassified COVID-19 cases, as the DHS collect data on URI symptoms but not on COVID-19 testing. This is an important limitation, indicating the need for caution when interpreting these results and future research that could help to clarify these uncertainties. Existing literature suggests that the non-pharmaceutical interventions implemented during the COVID-19 pandemic have not substantially affected pathogens that commonly cause URIs, such as rhinoviruses.^61^, ^62^ Another limitation is the potential underdiagnosis of COVID-19, which could affect URI incidence and mortality. Recent studies indicate that COVID-19 deaths might have been misclassified as non-COVID-19 respiratory conditions,^63^, ^64^, ^65^ highlighting the need for comprehensive investigations to understand the extent of such misclassification involving URIs.

The results of this study show the substantial burden imposed by URIs and otitis media. Although URIs and otitis media do not often result in severe illness or death, their high incidence rates and substantial morbidity should not be ignored. Furthermore, given the close association between URIs and otitis media, there is need for comprehensive strategies that address the prevention, early diagnosis, and effective management of these conditions together, particularly in young children, who are most affected. The potential for URIs to progress to more severe diseases, their contribution to AMR, and the pandemic potential of some URI pathogens necessitate comprehensive research and evidence-based strategies. Future studies are needed to better understand where strategies such as strengthening vaccination programmes, enhancing antibiotic stewardship, and promoting public health campaigns focused on hygiene and prevention are most needed.

### GBD 2021 Upper Respiratory Infections and Otitis Media Collaborators

### Affiliations

### Contributors

### Data sharing

To download the data used in these analyses, please visit the GHDx GBD 2021 website.

**This online publication has been corrected. The corrected version first appeared at thelancet.com/infection on December 27, 2024**

## Declaration of interests

S Afzal reports payment for educational events and webinars from King Edward Medical University and collaborative partners including Johns Hopkins University, University of California, University of Massachusetts, University of Nebraska, Imperial College London, KEMCA-UK, KEMCAANA, and APPNA; participation on a data safety monitoring board or advisory board with National Bioethics Committee Pakistan, the King Edward Medical University Institutional Ethical Review Board, and the Fatima Jinnah Medical University and Sir Ganga Ram Hospital Ethical Review Board; leadership or fiduciary roles in other board, society, committee or advocacy groups (paid or unpaid) with the Pakistan Association of Medical Editors, Faculty of Public Health Royal Colleges UK (fellow), Society of Prevention, Advocacy and Research at King Edward Medical University, and Pakistan Society of Infectious Diseases (member); and other financial or non-financial interests with the Corona Experts Advisory Group (member), Dengue Advisory Group (member), Technical Working Group or guidelines development for COVID-19 (member), National Command and Operation Committee of the Government of Pakistan (expert opinion), Pakistan Medical & Dental Council Research and Journals Committee (member), HEC Research and Publications Committee (member), Quality Assurance Agency HEC (member), Public Health and Preventive Medicine at King Edward Medical University (Dean), Quality Enhancement Cell at King Edward Medical University (director), *Annals of King Edward Medical University* (chief editor), and History Book of King Edward Medical University (chief editor), all outside the submitted work. S A Meo reports grants or contracts from the Deputyship for Research and Innovation, Ministry of Education in Saudi Arabia (IFKSUOR3-4-9), outside the submitted work. A Beloukas reports grants or contracts from Gilead (research grant and sponsorship to the University of West Attica) and GSK (research sponsorship to the University of West Attica); participation on a data safety monitoring board or advisory board with Gilead and GSK, paid to the University of West Attica; supports for attending meetings or travel from Gilead and GSK, paid to the University of West Attica; and receipt of equipment, materials, drugs, medical writing, gifts, or other services from Cepheid in the form of free-of-charge reagents for a research project; all outside the submitted work. C S Brown reports other financial support from market research companies via ad-hoc, one-off market research advisories on a variety of infection topics, all anonymous, conducted with no direct communication nor any knowledge of any pharmaceutical companies or products, outside the submitted work. I M Ilic and M D Ilic report support for the present manuscript from the Ministry of Science, Technological Development and Innovation of the Republic of Serbia (project numbers 175042, 2011-2023, 451-03-47/2023-01/200111). N E Ismail reports leadership or fiduciary roles in other board, society, committee, or advocacy groups (unpaid) as the Bursar and Council Member of the Malaysian Academy of Pharmacy and is a member of the Committee of the Malaysian Pharmacists Society Education Chapter, outside the submitted work. J J Jozwiak reports payment or honoraria for lectures, presentations, speakers bureaus, manuscript writing, or educational events from Novartis, Adamed, and Amgen, outside the submitted work. K Krishan reports non-financial support from the UGC Centre of Advanced Study, CAS II, awarded to the Department of Anthropology, Panjab University (Chandigarh, India), outside the submitted work. M-C Li reports grants or contracts from the National Science and Technology Council, Taiwan (NSTC 112-2410-H-003-031); and leadership or fiduciary roles in board, society, committee, or advocacy groups (paid or unpaid) as Technical Editor with the *Journal of the American Heart Association*, all outside the submitted work. L Monasta reports support from the Italian Ministry of Health (Ricerca Corrente 34/2017) and payments made to the Institute for Maternal and Child Health IRCCS Burlo Garofolo, outside the submitted work. C E Moore reports participation on an advisory board for an MRC grant with Gwen Knight (unpaid), WHO Advisory Group for the WHO Medically Important Antimicrobial List, and REVIVE Advisory Group as member of the steering group; and leadership or fiduciary roles in board, society, committee, or advocacy groups (unpaid) with the Microbiology Society as Co-chair of Impact and Influence Group, all outside the submitted work. A P Okekunle reports support for the present manuscript from the National Research Foundation of Korea funded by the Ministry of Science and ICT (2020H1D3A1A04081265) and support for attending meetings or travel from the National Research Foundation of Korea (funded by the Ministry of Science and ICT; 2020H1D3A1A04081265), outside the submitted work. E Ortiz-Prado reports grants or contracts from Universidad de las Americas, outside the submitted work. L F Reyes reports grants or contracts from MSD; consulting fees from GSK, MSD, and Pfizer; payment or honoraria for lectures, presentations, speakers bureaus, manuscript writing, or educational events from GSK, MSD, and Pfizer; payment for expert testimony from GSK and MSD; and support for attending meetings or travel from GSK and Pfizer, outside the submitted work. O Rezahosseini reports support for attending meetings or travel from the Research Department of Nordsjælands Hospital and European Society of Clinical Microbiology and Infectious Diseases 2024, outside the submitted work. L Ronfani reports support for the present manuscript from the Italian Ministry of Health (Ricerca Corrente 34/2017; payments made to the Institute for Maternal and Child Health IRCCS Burlo Garofolo). Y L Samodra reports a leadership or fiduciary role in a board, society, committee, or advocacy group (paid or unpaid) as co-founder of Benang Merah Research Center (bmrc.id), outside the submitted work. S Saxena reports grants or contracts and support for attending meetings or travel from the National Institute for Health and Care Research (NIHR) Senior Investigator Award, NIHR School for Public Health Research (grant number NIHR 204000), NIHR Northwest London Applied Research Collaboration; participation on a data safety monitoring board or advisory board with the BMJ International Editorial Board (advisory, unpaid) and the NIHR (Academy Chair, £7500 per annum honorarium paid to their institution); and leadership or fiduciary roles in board, society, committee, or advocacy groups (paid or unpaid) with the European Public Health Association as President of the Child and Adolescent Health Section, all outside the submitted work. B M Schaarschmidt reports research grants from Else Kröner-Fresenius Foundation, Deutsche Forschungsgemeinschaft, and PharmaCept GmbH; payment or honoraria for lectures, presentations, speakers bureaus, manuscript writing, or educational events from AstraZeneca; and support for travel from Bayer, all outside the submitted work. A Sharifan reports leadership or fiduciary roles in other board, society, committee, or advocacy groups (unpaid) as a steering committee member of Cochrane; and receipt of equipment, materials, drugs, medical writing, gifts, or other services from Elsevier, outside the submitted work. C R Simpson reports grants from HRC (New Zealand), Ministry of Health (New Zealand), MBIE (New Zealand), Chief Scientist Office (UK), and MRC (UK); and leadership or fiduciary roles in other board, society, committee, or advocacy groups (paid or unpaid) as a Chair of the New Zealand Government Data Ethics Advisory Group, outside the submitted work. J A Singh reports consulting fees from ROMTech, Atheneum, ClearView Healthcare Partners, American College of Rheumatology, Yale, Hulio, Horizon Pharmaceuticals, DINORA, Frictionless Solutions, Schipher, Crealta/Horizon, Medisys, Fidia, PK Med, Two Labs, Adept Field Solutions, Clinical Care Options, Putnam Associates, Focus Forward, Navigant Consulting, Spherix, MedIQ, Jupiter Life Science, UBM, Trio Health, Medscape, WebMD, Practice Point Communications, and the National Institutes of Health; payment or honoraria for lectures, presentations, speakers bureaus, manuscript writing, or educational events on the speakers bureau of Simply Speaking; support for attending meetings or travel from OMERACT as a steering committee member; participation on a data safety monitoring board or advisory board with the FDA Arthritis Advisory Committee; leadership or fiduciary roles in other board, society, committee, or advocacy groups as a past steering committee member of the OMERACT (an international organisation that develops measures for clinical trials and receives arm's-length funding from 12 pharmaceutical companies; paid), as Chair of the Veterans Affairs Rheumatology Field Advisory Committee (unpaid), and as the Editor and Director of the UAB Cochrane Musculoskeletal Group Satellite Center on Network Meta-analysis (unpaid); stock or stock options in Atai Life Sciences, Kintara Therapeutics, Intelligent Biosolutions, Acumen Pharmaceutical, TPT Global Tech, Vaxart Pharmaceuticals, Atyu Biopharma, Adaptimmune Therapeutics, GeoVax Labs, Pieris Pharmaceuticals, Enzolytics, Seres Therapeutics, Tonix Pharmaceuticals, Aebona Pharmaceuticals, and Charlotte's Web, and previously owned stock options in Amarin, Viking, and Moderna Pharmaceuticals, outside the submitted work. J H V Ticoalu reports leadership or fiduciary roles in board, society, committee, or advocacy groups (paid or unpaid) with Benang Merah Research Center (bmrc.id) as a co-founder, outside the submitted work. E Upadhyay reports the following published patents: a system and method of reusable filters for anti-pollution mask; a system and method for electricity generation through crop stubble by using microbial fuel cells; a system for disposed personal protection equipment into biofuel through pyrolysis and method; and a novel herbal pharmaceutical aid for formulation of gel and method thereof. E Upadhyay also reports patents filed: herbal drug formulation for treating lung tissue degenerated by particulate matter exposure; and a method to transform cow dung into the wall paint by using natural materials and composition thereof. Additionally, E Upadhyay reports leadership or fiduciary roles in other board, society, committee, or advocacy groups (paid or unpaid) as Joint Secretary of Indian Meteorological Society, Jaipur Chapter (India), and as Member-Secretary of DSTPURSE Program, outside the submitted work. M Zielińska reports other financial interests as an employee of AstraZeneca, outside the submitted work.
