# Correction to *Lancet Infect Dis* 2025; 25: 36–51

**DOI:** 10.1016/S1473-3099(24)00830-2

**Published:** 2025-01

*GBD 2021 Upper Respiratory Infections and Otitis Media Collaborators. Global, regional, and national burden of upper respiratory infections and otitis media, 1990–2021: a systematic analysis from the Global Burden of Disease Study 2021.* Lancet Infect Dis *2024; **25:** 36–51*—In this Article, the second-to-last sentence of the Research in Context panel should have read “Implementing targeted preventive interventions could help to reduce the burden of URIs and otitis media in children younger than 2 years, who account for the highest rate of episodes across all age groups.” This correction has been made to the online version as of Dec 27, 2024 and the printed version is correct.

