# Supplementary material for: Global, regional, and national burden of upper respiratory infections and otitis media, 1990–2021: a systematic analysis from the Global Burden of Disease Study 2021
Source: Lancet Infect Dis. 2025 Jan;25(1):36–51. doi: 10.1016/S1473-3099(24)00430-4 (PMC11680489; doi:10.1016/S1473-3099(24)00430-4)
Supplement: Supplementary appendix 1 [file mmc1.pdf]

THE LANCET
Infectious Diseases

Supplementary appendix 1

This appendix formed part of the original submission and has been peer reviewed. We post it as supplied by the authors.

Supplement to: GBD 2021 Upper Respiratory Infections and Otitis Media Collaborators. Global, regional, and national burden of upper respiratory infections and otitis media, 1990–2021: a systematic analysis from the Global Burden of Disease Study 2021. *Lancet Infect Dis* 2024; published online Sept 9. https://doi.org/10.1016/S1473-3099(24)00430-4.

Appendix 1

"Global, regional, and national burden of upper respiratory infections and otitis media, 1990-2021: results from the Global Burden of Disease Study 2021"

This appendix provides further methodological detail and results for "Global, regional, and national burden of upper respiratory infections and otitis media, 1990-2021: results from the Global Burden of Disease Study 2021"

All the material in the paper itself is novel although it builds off previous GBD works. However, parts of the supplemental methods appendix include sections adapted from the GBD Capstones previously published in The Lancet.[1–3]

APPENDIX 1 .... **1**

"GLOBAL, REGIONAL, AND NATIONAL BURDEN OF UPPER RESPIRATORY INFECTIONS AND OTITIS MEDIA, 1990-2021: RESULTS FROM THE GLOBAL BURDEN OF DISEASE STUDY 2021" **1**

GBD REGIONS **3**

FATAL UPPER RESPIRATORY INFECTIONS **3**

INPUT DATA AND METHODOLOGICAL SUMMARY FOR UPPER RESPIRATORY INFECTIONS 4
INPUT DATA 4
MODELLING STRATEGY 4

NON-FATAL UPPER RESPIRATORY INFECTIONS **5**

CASE DEFINITION 5
INPUT DATA 5
Model inputs *5*
Severity splits *7*
MODELLING STRATEGY 8

FATAL OTITIS MEDIA **11**

INPUT DATA AND METHODOLOGICAL SUMMARY FOR OTITIS MEDIA 11
Input data *11*
Modelling strategy *11*

NON-FATAL OTITIS MEDIA **12**

CASE DEFINITION 12
INPUT DATA .... 13~~13~~
MODELLING STRATEGY .... 15~~15~~

RISK FACTOR ESTIMATION .... **16~~16~~**

CRITERIA FOR INCLUSION OF RISK-OUTCOME PAIRS .... 16~~16~~
AMBIENT PARTICULATE MATTER POLLUTION .... 17~~17~~
FLOWCHART .... 17~~17~~
INPUT DATA AND METHODOLOGICAL SUMMARY .... 17~~17~~
EXPOSURE .... 17~~17~~
THEORETICAL MINIMUM-RISK EXPOSURE LEVEL .... 23~~23~~
RELATIVE RISKS AND POPULATION ATTRIBUTABLE FRACTIONS .... 23~~23~~
HOUSEHOLD AIR POLLUTION 41
Input data and methodological summary *41*
Exposure *41*
Theoretical minimum-risk exposure level .... *44~~44~~*
Relative risks .... *45~~45~~*
SECONDHAND SMOKE .... 49~~49~~
Exposure *50*
Population attributable fraction .... *62~~63~~*
LOW BIRTHWEIGHT AND SHORT GESTATION .... 63~~64~~
Input data and methodological summary .... *64~~65~~*
Data processing .... *67~~68~~*
Relative risks and theoretical minimum risk exposure level .... *73~~74~~*

STATEMENT OF GATHER COMPLIANCE .... **76~~76~~**

REFERENCES .... **78~~7~~8**

GBD Regions

Map of GBD Regions and Super-Regions

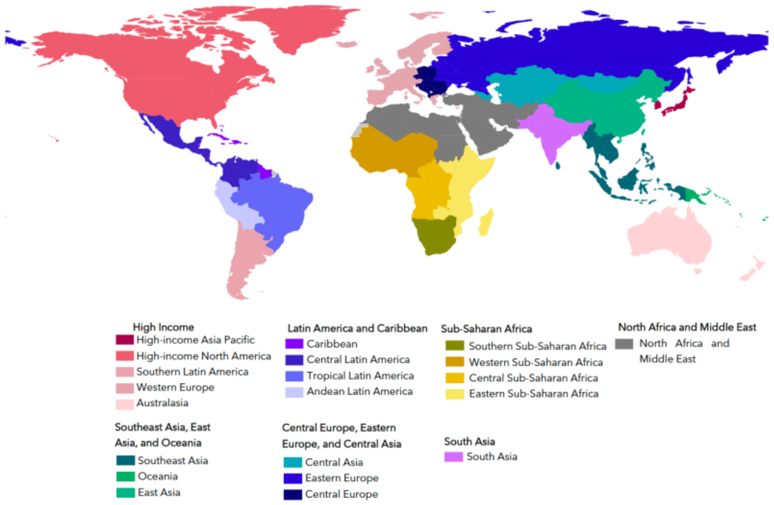

Fatal Upper respiratory infections

Appendix Figure 1: Flowchart of URI mortality estimation

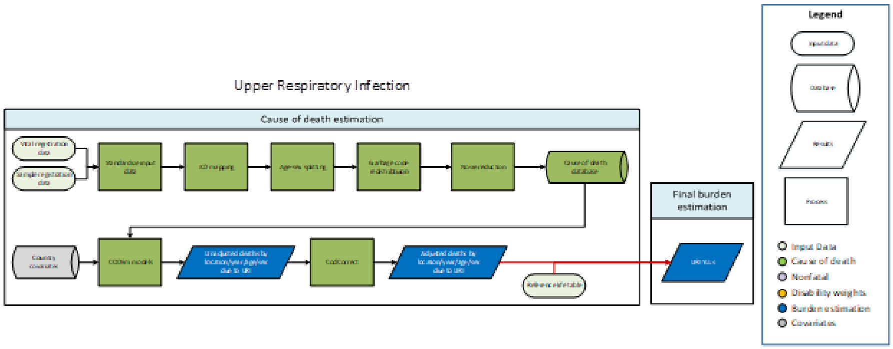

Input data and methodological summary for upper respiratory infections

Input data

Vital registration and surveillance data from the cause of death (CoD) database were used. Outliers were identified by systematic examination of datapoints. Datapoints that violated well-established age or time trends, were inconsistent with other country- or region-specific points, or that resulted in extremely high or low mortality rates were determined to be outliers.

Modelling strategy

A generic CODEm approach was used to estimate mortality due to upper respiratory infections (URI) in GBD 2021. In GBD 2016, mortality from URI was modelled using a negative binomial regression. It was determined that a negative binomial regression was an appropriate approach for estimating URI due to a small number of deaths due to URI in the CoD database. However, due to changes in how we redistribute cause of death codes, more deaths were attributed to URI in the CoD database, and thus it was determined that a generic CODEm approach was feasible for estimating URI mortality in GBD 2017. The covariates used are displayed below. We have made no substantive changes to the modelling strategy in 2021.

Appendix Table 1: Covariates used for URI cause-of-death ensemble modelling

Level	Covariate	Direction
1	Smoking prevalence	+
2	Indoor pollution	+
	Outdoor pollution ($PM_{2.5}$)	+
	Healthcare Access and Quality Index	-
3	Socio-demographic Index	-
	Lag distributed income	-
	Education (years per capita)	-

Non-fatal Upper respiratory infections

Appendix Figure 2: Flowchart of URI non-fatal burden estimation

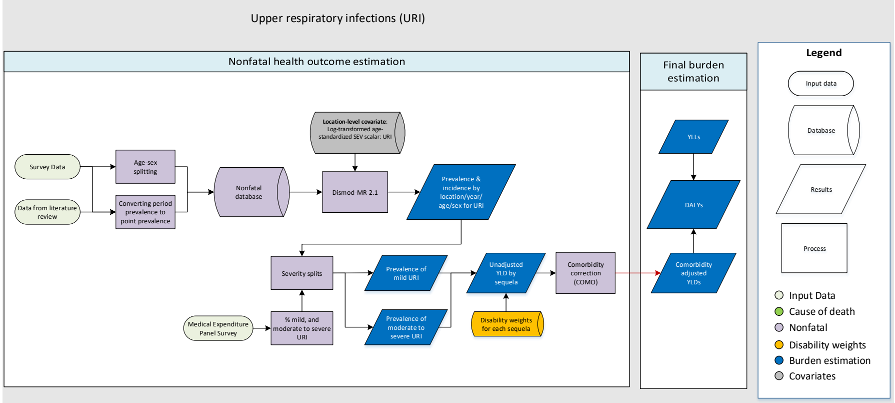

Case definition

Upper respiratory infections (URI) are characterized by sore throat, low-grade fever, and an adherent membrane of the tonsil(s), pharynx, and/or nose without other apparent cause. URIs include cough, acute nasopharyngitis, sinusitis, pharyngitis, tonsillitis, laryngitis/tracheitis, epiglottitis, rhinitis, rhinosinusitis, rhinopharyngitis, supraglottitis, and the common cold. For URI, ICD-10 codes are J00-J02, J02.8-J03, J03.8-J06.9, J36, J36.0, and ICD-9 codes are 460-465.9, 475-475.9, 476.9.

Input data

Model inputs

For GBD 2021, a systematic review of URI was conducted using the following PubMed search string:

((upper respiratory infection[Title/Abstract] or rhinitis[Title/Abstract] or rhinitis[MeSH] or rhinosinusitis[Title/Abstract] or sinusitis[Title/Abstract] or sinusitis[MeSH] or nasopharyngitis[Title/Abstract] or rhinopharyngitis[Title/Abstract] or common cold[Title/Abstract] or common cold[MeSH] or pharyngitis[Title/Abstract] or pharyngitis[MeSH] or tonsillitis[Title/Abstract] or epiglottitis[Title/Abstract] or supraglottitis[Title/Abstract] or supraglottitis[MeSH] or laryngitis[Title/Abstract] or laryngitis[MeSH] or laryngotracheitis[Title/Abstract] or tracheitis[Title/Abstract] or tracheitis[MeSH]) AND (prevalence[Title/Abstract] OR incidence[Title/Abstract] OR remission[Title/Abstract] OR duration[Title/Abstract]) NOT (allergies or allergy or allergic rhinitis or asthma) AND (2019/02/07[PDAT] : 2020/12/31[PDAT]) NOT (animals[MeSH] NOT humans[MeSH])

The exclusion criteria for both systematic reviews were:

1. Studies that were not population-based, eg, hospital or clinic-based studies.
2. Studies that did not provide primary data on epidemiological parameters, eg, a commentary piece.
3.
4. Reviews.

We identified 266 studies via PubMed, of which none met the above inclusion criteria. Given the low yield of the most recent systematic review, we will prioritise adding data from national surveys as opposed to journal articles in future rounds, given that we expect comprehensive, national surveys to be more likely to estimate the burden of URI.

Appendix Figure 3: Prisma Diagram of systematic review

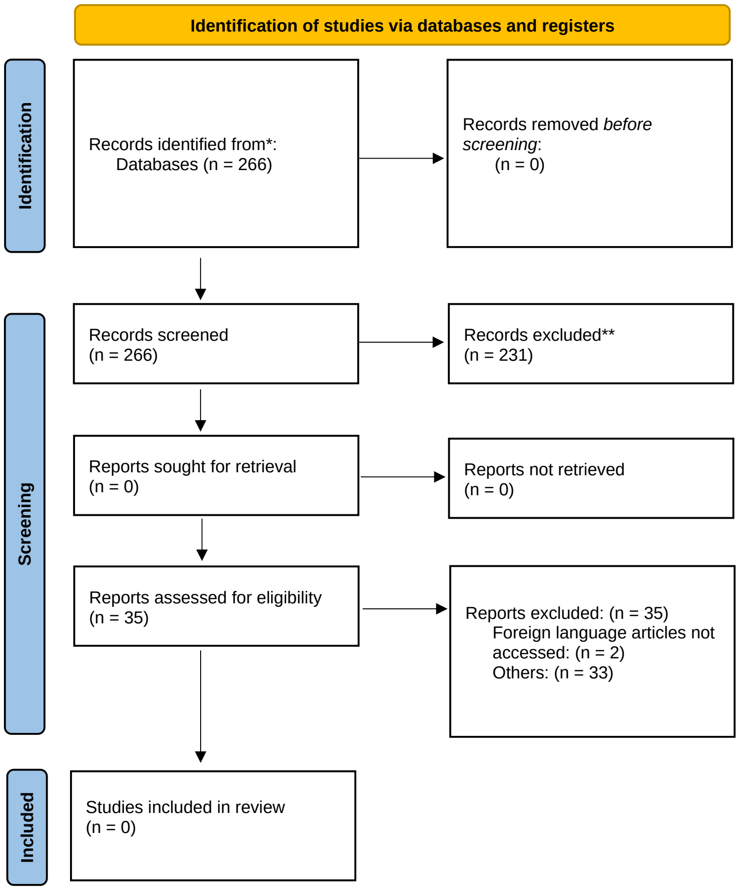

In addition, data from nationally representative surveys including United States National Health Interview Surveys and Demographic and Health Surveys were included. For these surveys, nationally representative is defined as a population-based survey with large random sample sizes where nearly all people within the country had a non-zero chance of being sampled. The process of determining whether a survey was representative involved examining the type of population that each survey covered. Surveys that provide insights into the general population of a country, region, or any geographical area are considered to be population-representative. The Demographic and Health Surveys (DHS) and Multiple Indicator Cluster Surveys (MICS) are good examples of such representative surveys. Conversely, studies focusing on distinct subsets of the

population, particularly marginalized groups such as refugees, prisoners, or individuals who inject drugs, do not align with our representativeness criteria. We applied sampling weights to adjust for unequal probabilities of selection and non-responses to ensure representative estimates of the population.

The definition of upper respiratory infections from these surveys was the two-week period prevalence of cough. We assume that cough without difficulty breathing, along with or without a fever, is the definition of upper respiratory infection. We converted these data from two-week period prevalence to point prevalence assuming a duration of five days. The equation for this adjustment is:

$$Point\ Prevalence = \frac{Period\ Prevalence * Duration}{(Recall\ Period + Duration - 1)}$$

Newly identified data sources were added to sources and studies identified in previous rounds of the GBD, resulting in a total of 239 unique data sources from 76 countries **(Appendix Table 2).**

Appendix Table 2: Data inputs for URI morbidity modeling by parameter

Measure	Total sources	Countries with data
All measures	321	81
Prevalence	303	81
Incidence	3	1
Proportion	15	1

Severity splits

The table below shows the severity distributions based on the data from Medical Expenditure Panel Surveys where we categorised "acute nasopharyngitis or acute URI multi sites/nos" as mild URI and "acute sinusitis, acute pharyngitis, acute tonsillitis, and acute laryngitis/tracheitis and epiglottitis" as moderate URI.

Appendix Table 3: URI severity split proportions

**Mild URI proportion**	**Moderate URI proportion**
0.56 (0.43–0.68)	0.44 (0.32–0.57)

The lay descriptions and disability weights for severity levels derived from the GBD disability weights study are shown below.

Appendix Table 4: URI severity split disability weights

Severity level	Lay description		DW (95% CI)
Mild upper respiratory infections	Has a low fever and mild discomfort, but no difficulty with daily activities		0.006 (0.002–0.012)
Moderate/severe upper respiratory infections	Has a fever and aches, and feels weak, which causes		0.051 (0.032–0.074)

	some difficulty with daily activities		

Modelling strategy

URI was modelled using a standard DisMod-MR 2.1 model using secondhand smoke as the location-level covariate.

Betas and exponentiated values are shown in the table below:

Appendix Table 4: URI modelling covariates

Covariate	Parameter	Beta	Exponentiated beta
Secondhand smoke	Prevalence	0.11	1.15 (1.01–1.31)
Sex	Prevalence	–0.027	1.00 (0.99–1.02)

DisMod-MR 2.1 description

The sequence of estimation in DisMod MR 2.1[1] occurs at five levels: global, super-region, region, country and, where applicable, subnational location. The super-region priors are generated at the global level with mixed-effects, nonlinear regression using all available data; the super-region fit, in turn, informs the region fit, and so on down the cascade. Subnational estimation was informed by the country fit and country covariates, plus an adjustment based on the average of the residuals between the subnational location's available data and it's prior. This mimicked the impact of a random effect on estimates between subnationals. At each level of the cascade, the DisMod-MR 2.1 enforces consistency between all parameters. Analysts have the choice to branch the cascade in terms of time and sex at different levels depending on data density.[1] We used the default option to model LRI, which is to branch by sex after the global fit but to retain all years of data until the lowest level in the cascade.

The coefficients for country covariates were re-estimated at each level of the cascade. For a given location, country coefficients were calculated using both data and prior information available for that location. In GBD 2021, we generated model fits for the years 1990, 1995, 2000, 2005, 2010, 2015, 2019, 2020 and 2021, and log-linearly interpolated estimates for the intervening years. Convergence was assessed qualitatively by visually inspecting diagnostic plots of the posterior distributions. The 95% uncertainty intervals were computed based on 1000 draws from the posterior distribution of the converged model using the 2.5th and 97.5th percentiles of the ordered 1000 values.

Analysts have the choice of using a Gaussian, log-Gaussian, Laplace or Log-Laplace likelihood function in DisMod-MR 2.1. We used the default log-Gaussian equation for the data likelihood, which is:

$$-log[p(y_j|\Phi)] = \log(\sqrt{2\pi}) + \log(\delta_j + s_j) + \frac{1}{2}\left(\frac{\log(a_j + \eta_j) - \log(m_j + \eta_j)}{\delta_j + s_j}\right)^2$$

where, y_j is a 'measurement value' (i.e., data point); Φ denotes all model random variables; η_j is the offset value, eta, for a particular 'integrand' (prevalence, incidence, remission, excess mortality rate, cause-specific mortality rate) and a_j is the adjusted measurement for data point j, defined by:

$$a_j = e^{(-u_j - c_j)} y_j$$

where u_j is the total 'area effect' (i.e., the sum of the random effects at three levels of the cascade: super-region, region and country) and c_j is the total covariate effect (i.e., the mean combined fixed effects for sex, study level and country level covariates), defined by:

$$c_j = \sum_{k=0}^{K[I(j)]-1} \beta_{I(j),k} \hat{X}_{k,j}$$

with standard deviation

$$s_j = \sum_{l=0}^{L[I(j)]-1} \zeta_{I(j),l} Z_{k,j}$$

where k denotes the mean value of each data point in relation to a covariate (also called x-covariate); I(j) denotes a data point for a particular integrand, j; $\beta_{I(j),k}$ is the multiplier of the kth x-covariate for the ith integrand; $\hat{X}_{k,j}$ is the covariate value corresponding to the data point j for covariate k; l denotes the

standard deviation of each data point in relation to a covariate (also called z-covariate); $\zeta_{I(j),k}$ is the multiplier of the lth z-covariate for the ith integrand; and δ_j is the standard deviation for adjusted measurement j, defined by:

$$\delta_j = log[y_j + e^{(-uj-c_j)}\eta_j + c_j] - log[y_j + e^{(-uj-c_j)}\eta_j]$$

Where m_j denotes the model for the jth measurement, not counting effects or measurement noise and defined by:

$$m_j = \frac{1}{B(j)-A(j)} \int_{A(j)}^{B(j)} I_j(a)\, da$$

where A(j) is the lower bound of the age range for a data point; B(j) is the upper bound of the age range for a data point; and I_j denotes the function of age corresponding to the integrand for data point j.

The source code for DisMod-MR 2.1 as well as the wrapper code is available at the following link: https://github.com/ihmeuw/ihmemodelling/tree/master/gbd_2017/shared_code/central_comp/nonfatal/dismod.

Fatal Otitis media

Appendix Figure 4: Flowchart of otitis mortality estimation

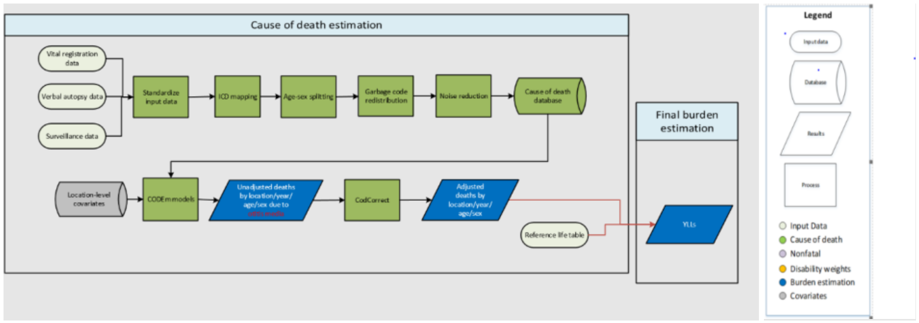

Input data and methodological summary for otitis media

Input data

Vital registration, verbal autopsy, and surveillance data were used. Outliers were identified by systematic examination of datapoints. Datapoints that violated well-established age or time trends were inconsistent with other country- or region-specific points, or that resulted in extremely high or low mortality rates were determined to be outliers.

Modelling strategy

A general CODEm modelling strategy was used. There were no substantive changes from GBD 2019 in terms of modelling strategy. The covariates used are displayed in Table 1.

Appendix Table 5: Covariates used for otitis cause-of-death ensemble modelling

Level	Covariate	Direction
1	Otitis summary exposure value (SEV)	+
	Smoking prevalence	+
2	Indoor pollution	+
	Healthcare Access and Quality Index	-
	Outdoor pollution ($PM_{2.5}$)	+
3	Socio-demographic Index (SDI)	-
	Log-transformed lag distributed income	-
	Education (years per capita)	-

Non-fatal Otitis media

Appendix Figure 5: Flowchart of otitis non-fatal burden estimation

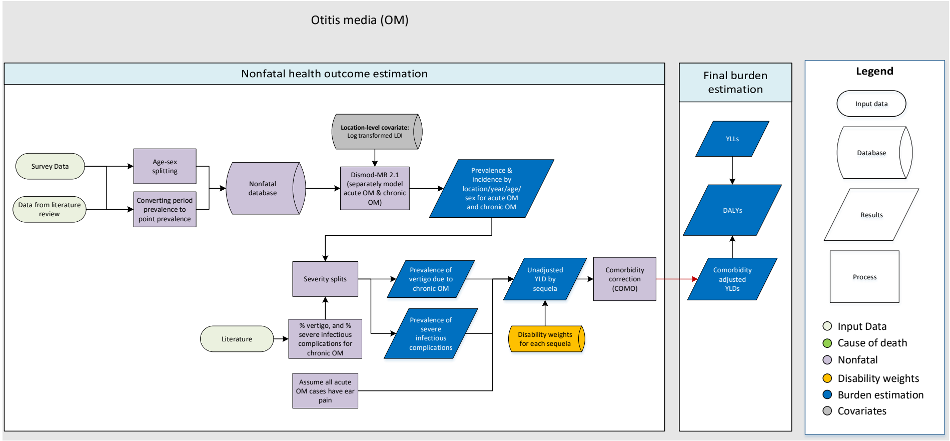

Case definition

Otitis media is an infection of the middle ear space. We included acute otitis media, chronic otitis media, and hearing loss due to chronic otitis media in the GBD non-fatal outcome modelling. Hearing loss due to chronic otitis media estimation is included in the hearing loss report provided separately. The ICD-10 codes are H65-H75.83, and ICD-9 codes are 381-384.9.

Appendix Table 6: Reference and alternative definitions used for otitis

**Quantity of interest**	**Reference or Alternative**	**Definition**
Incidence of acute otitis media	Reference	Cases of acute otitis media from clinical diagnosis, surveys, or literature.
Incidence of chronic otitis media	Reference	Cases of chronic otitis media from surveys or literature.
Prevalence of acute otitis media	Reference	Cases of acute otitis media from clinical diagnosis, surveys, or literature.
Prevalence of chronic otitis media	Reference	Cases of chronic otitis media from surveys or literature.
Remission of chronic otitis media	Reference	The rate at which chronic otitis media cases stop meeting the ICD diagnostic criteria.

Input data

A systematic review of the incidence and prevalence of otitis media was conducted for GBD 2021. The PubMed search terms were: (otitis media[Title/Abstract] AND (inciden*[Title/Abstract] OR prevalen*[Title/Abstract] OR remission[Title/Abstract] OR duration[Title/Abstract]) AND ("2017/10/01"[PDAT] : "3000"[PDAT]) NOT (animals[MESH] NOT humans[MESH]))

The exclusion criteria were:

1. Studies that were not population-based, e.g., hospital or clinic-based studies
2. Studies that did not provide primary data on epidemiological parameters, e.g., commentaries
3. ~~Studies with a sample size of less than 150~~
~~4.~~ 3. Reviews
~~5.~~ 4. Case series

Appendix Figure 6: Prisma Diagram of systematic review

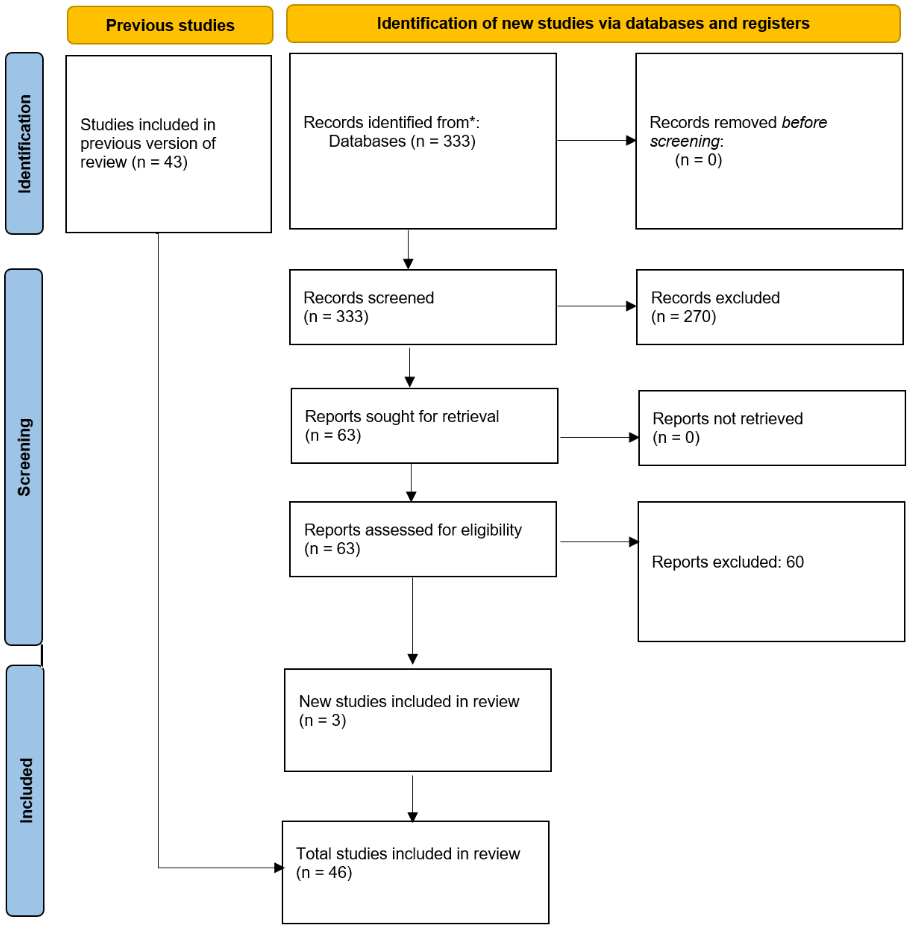

In addition, CF3-corrected data from inpatient and outpatient claims were included in the acute otitis model.

Appendix Table 7: Data inputs for otitis media morbidity modeling by parameter

	Countries with data	New sources	Total sources
Incidence	10	3	52
Prevalence	21	3	33
Remission	4	0	5
Other	0	0	0

Modelling strategy

We assume that all acute otitis media cases would experience ear pain. The severity distributions for chronic otitis media based on the study by Lin and colleagues (2009)[4] were as follows: (i) vertigo (2.9%, 95% CI: 2.4–3.6), and (ii) severe infectious complications (0.05%, 95% CI: 0.01–0.2). We assumed that all chronic otitis media cases experience either mild or moderate hearing loss. The lay descriptions and disability weights for severity levels derived from the GBD disability weights study are shown below.

Table 2. Severity distribution, details on the severity levels for otitis media and the associated disability weight (DW) with that severity.

Appendix Table 8: Otitis media severity split disability weights

Severity level	Lay description	DW (95% CI)
Acute otitis media	Has an earache that causes some difficulty with daily activities.	0.013 (0.007–0.024)
Severe infectious complications due to chronic otitis media	Has an earache that causes some difficulty with daily activities.	0.013 (0.009–0.019)
Mild hearing loss due to chronic otitis media	Has great difficulty hearing and understanding another person talking in a noisy place (for example, on an urban street).	0.01 (0.004–0.019)
Moderate hearing loss due to chronic otitis media	Is unable to hear and understand another person talking in a noisy place (for example, on an urban street), and has difficulty hearing another person talking even in a quiet place or on the phone.	0.027 (0.015–0.042)
Mild hearing loss with ringing due to chronic otitis media	Has great difficulty hearing and understanding another person talking in a noisy place (for example, on an urban street), and sometimes has annoying ringing in the ears.	0.021 (0.012–0.036)
Moderate hearing loss with ringing due to chronic otitis media	Is unable to hear and understand another person talking in a noisy place (for example, on an urban street), and has difficulty hearing another person talking even in a quiet place or on the phone, and has annoying ringing in the ears for	0.074 (0.049–0.107)

	more than 5 minutes at a time, almost every day.	
Vertigo with mild hearing loss due to chronic otitis media	*	0.122 (0.079–0.17)
Vertigo with mild hearing loss and ringing due to chronic otitis media	*	0.132 (0.086–0.184)
Vertigo with moderate hearing loss due to chronic otitis media	*	0.137 (0.089–0.189)
Vertigo with moderate hearing loss and ringing due to chronic otitis media	*	0.179 (0.12–0.247)

* See the hearing loss report for the lay descriptions and disability weights for different severity levels.

We modelled acute and chronic otitis media as separate non-fatal health outcomes using DisMod-MR 2.1. Log-transformed LDI covariate was used as a country-level covariate to model chronic otitis media.

Appendix Table 9: Covariates used for otitis media non-fatal modelling

**Covariate**	**Type**	**Parameter**	**Exponentiated beta (95% CI)**
Sex	Study-level	Prevalence	0.99 (0.66–1.50)
Sex	Study-level	Incidence	0.79 (0.78–0.80)

Appendix Table 10: Covariates used for chronic otitis media non-fatal modelling

**Covariate**	**Type**	**Parameter**	**Exponentiated beta (95% CI)**
Log LDI	Country-level	Prevalence	0.72 (0.63–0.82)
Sex	Study-level	Prevalence	1.14 (0.98–1.32)
Sex	Study-level	Incidence	1.26 (0.66–2.49)

Risk Factor Estimation

Criteria for inclusion of risk-outcome pairs

GBD requires each risk-outcome-pair to have convincing or probable evidence of a causal relationship between exposure and disease, established from multiple epidemiological studies in different populations.[3]

Ambient particulate matter pollution

Flowchart

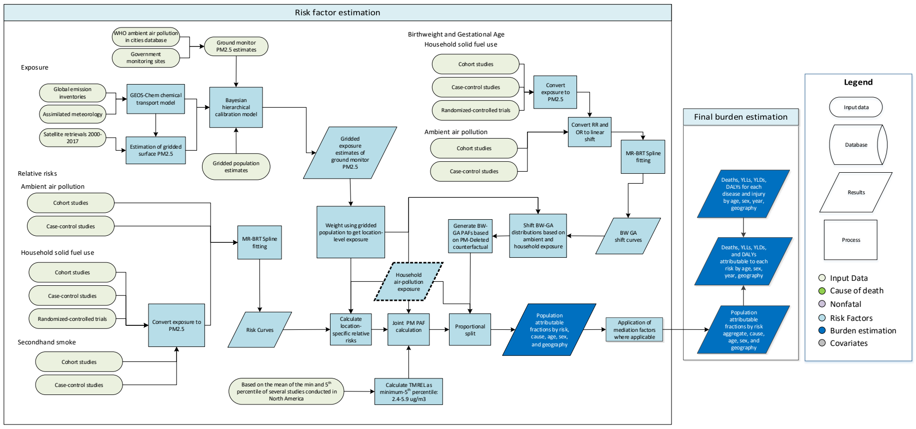

Input data and methodological summary

Exposure

Definition

Exposure to ambient particulate matter pollution is defined as the population-weighted annual average mass concentration of particles with an aerodynamic diameter less than 2.5 micrometers ($PM_{2.5}$) in a cubic meter of air. This measurement is reported in µg/m^3.

Input data

Ambient air pollution exposure estimates use input data from multiple sources. These include satellite observations of aerosols in the atmosphere, ground monitor measurements, chemical transport model simulations, population estimates, and land-use data.

Table 1: Data inputs for exposure for ambient particulate matter pollution

Input data	Exposure
Site-years (total)	5442
Number of countries with data	204
Number of GBD regions with data (out of 21 regions)	21
Number of GBD super-regions with data (out of 7 super-regions)	7

Details for updates in exposure methodology and input data for the Global Burden of Disease (GBD) Study 2021 are as follows.

$PM_{2.5}$ ground measurement database

For GBD 2021, ground monitor measurements were updated to include more recent measurements from sites included in GBD 2019 and additional measurements from new monitors. New data were

added to the database from several sources, including the European Environment Agency, United States Environmental Protection Agency, and the OpenAQ database. The complete, updated dataset included measurements of PM_{10} and $PM_{2.5}$ concentrations between 2018 and 2020 from 18,406 ground monitors from 120 countries, primarily from the USA, China, European countries, and USA embassies and consulates. Annual averages were excluded if they were based on less than 75% coverage within a year unless there was already sufficient data within the country of interest (monitor density greater than 0.1). If information on coverage was not available, data were included.

For sites with PM_{10} measurements only, these observations were converted from PM_{10} to $PM_{2.5}$ measurements using a hierarchy of conversion factors ($PM_{2.5}/PM_{10}$ ratios): (i) where possible, a "local" conversion factor was used, constructed as the ratio of the average measurements (of $PM_{2.5}$ and PM_{10}) from within 50 km of the location of the PM_{10} measurement, and within the same country, if such measurements were available; (ii) where local information was not sufficient to construct a conversion factor, a country-wide conversion factor was used; and (ii) where appropriate information within a country did not exist, a region-level factor was used. In each case, to avoid the possible effects of outliers in the measured $PM_{2.5}$ and PM_{10} data, extreme values of the ratios were excluded. These extreme values were defined as those greater/lesser than the 95th and 5th quantiles of the empirical distributions of conversion factors. As with the GBD 2013, 2015, 2016, 2017, and 2019 databases, in addition to values of $PM_{2.5}$ and whether they were direct measurements or conversions from PM_{10}, the GBD 2021 database also included additional information (where available) concerning the ground measurements, such as monitor geo-coordinates and monitor site type.

Satellite-based estimates

Global satellite-derived estimates (V4.GL.03.NoGWR) used as inputs to DIMAQ2 for 1998–2019 and for January to August 2020 are used at 0.1^{o} x 0.1^{o} resolution (~11 x 11 km resolution at the equator) and follow the methodology described in Hammer et al., 2020.[5] The algorithm uses aerosol optical depth (AOD) from several updated satellite products (MAIAC, MODIS, and MISR). Ground-based observations from a global sunphotometer network (AERONET version 3) are used to combine different AOD information sources. The GEOS-Chem chemical transport model was used for geophysical relationships between surface $PM_{2.5}$ and AOD. For GBD 2021, an additional update to biomass burning emissions from 2015 to 2020 was made. This update allows for time-varying biomass burning emissions in the simulation for those years, where they had previously been unavailable after 2014. Given lags in releases of available meteorological information used in the GEOS Chem simulations, for September to December 2020, the estimates incorporate satellite retrievals from 2020, but GEOS-Chem simulated values for 2019 as well as biomass burning emissions from 2019. Further, satellite retrievals for all of 2020 were limited to MODIS DT, DB, and MAIAC. We included MISR inputs for January to June 2020 only, as this product was not available past June when the satellite-based estimates were generated.

Chemical transport model simulations

Estimates of the sum of particulate sulfate, nitrate, ammonium, and organic carbon and the compositional concentrations of mineral dust simulated using the GEOS-Chem chemical transport model, and a measure combining elevation and the distance to the nearest urban land surface (as described in van Donkelaar et al. 2016[6] and Hammer et al. 2020)[5] were available for 2000–2020 for each $0.1^{o} \times 0.1^{o}$ grid cell.

Population data

We obtained a comprehensive, high-resolution gridded population dataset from the Gridded Population of the World (GPW) database. Estimates for 2000, 2005, 2010, 2015, and 2020 were available from the GPW version 4, with estimates for 1990 and 1995 obtained from the GPW version 3. These data are provided on a 0.0083° × 0.0083° resolution. Aggregation to each 0.1° × 0.1° grid cell was accomplished by summing the central 12 × 12 population cells. Populations estimates for 2001–2004, 2006–2009, 2011–2014, and 2016–2019 were obtained by interpolation using natural splines with knots placed at 2000, 2005, 2010, 2015, and 2020. This was performed for each grid cell.

Modelling strategy

The following is a summary of the modelling approach, known as the Data Integration Model for Air Quality (DIMAQ) used in GBD 2015, 2016, 2017, 2019, and 2020. [7,8]

Before the implementation of DIMAQ in GBD 2010 and 2013, exposure estimates were obtained using a single global function to calibrate available ground measurements to a “fused” estimate of $PM_{2.5}$: the mean of satellite-based estimates and those from the TM5 chemical transport model, calculated for each 0.1° × 0.1° grid cell. This approach was recognised to represent a trade-off between accuracy and computational efficiency when utilising all the available data sources. In particular, the GBD 2013 exposure estimates were known to underestimate ground measurements in specific locations (see discussion in Brauer et al., 2015).[9] This underestimation was largely due to the use of a single, global calibration function, whereas in reality, the relationship between ground measurements and other variables varies spatially.

In GBD 2015 and 2016, coefficients in the calibration model were estimated for each country through DIMAQ. Where data were insufficient within a country, information was “borrowed” from a region-level aggregation, and where information was still insufficient, from the super-region-level aggregation. Individual country-level estimates were therefore based on a combination of information from the country and its region and super-region. This was implemented within a Bayesian hierarchical modelling (BHM) framework. BHMs provide an extremely useful and flexible framework in which to model complex relationships and dependencies in data. Uncertainty can also be propagated through the model, allowing uncertainty arising from different components (both data sources and models) to be incorporated within estimates of uncertainty associated with the final estimates. The results of the modelling comprise a posterior distribution for each grid cell, rather than just a single point estimate, allowing a variety of summaries to be calculated. The primary outputs for this process are the median and 95% uncertainty intervals for each grid cell. Based on the availability of ground measurement data, modelling and evaluation were focused on the year 2016.

The model used from GBD 2017 onward (GBD 2017, 2019, and now 2021) also included within-country calibration variation.[7] This model, henceforth referred to as DIMAQ2, provides a number of substantial improvements over the initial formulation of DIMAQ. In DIMAQ, ground measurements from different years were all assumed to have been made in the primary year of interest and then regressed against values from other inputs (satellites, etc.) made in that year. In the presence of changes over time, therefore, and particularly in areas where no recent measurements were available, there was the possibility of mismatches between the ground measurements and other variables. In DIMAQ2, ground measurements are matched with other inputs (over time), and the (global-level) coefficients are allowed to vary over time, subject to smoothing that is induced by a first-order random walk process. In addition,

the manner in which spatial variation can be incorporated within the model has developed: where there are sufficient data, the calibration equations can now vary (smoothly) both within and between countries, achieved by allowing the coefficients to follow (smooth) Gaussian processes. Where there are insufficient data within a country, to produce accurate equations, information is borrowed as before from lower down the hierarchy and is supplemented with information from the wider region.

DIMAQ2 as described above was used for all regions except for the north Africa/Middle East and sub-Saharan Africa super-regions, where there are insufficient data across years to allow the extra complexities of the new model to be implemented. In these super-regions, a simplified version of DIMAQ2 is used in which the temporal component is dropped.

Inference and prediction

Continuous explanatory variables:

- (SAT) Estimate of $PM_{2.5}$ (in μg/m^3) from satellite remote sensing on the log-scale.
- (POP) Estimate of population for the same year as SAT on the log-scale.
- (SANOC) Estimate of the sum of sulfate, nitrate, ammonium, and organic carbon simulated using the GEOS-Chem chemical transport model.
- (DST) Estimate of compositional concentrations of mineral dust simulated using the GEOS-Chem chemical transport model.
- (EDxDU) The log of the elevation difference between the elevation at the ground measurement location and the mean elevation within the GEOS-Chem simulation grid cell multiplied by the inverse distance to the nearest urban land surface.

Discrete explanatory variables:

- (LOC) Binary variable indicating whether exact location of ground measurement is known.
- (TYPE) Binary variable indicating whether exact type of ground monitor is known.
- (CONV) Binary variable indicating whether ground measurement is $PM_{2.5}$ or converted from PM_{10}.

Interactions*:*

- Interactions between the binary variables and the effects of SAT.

Random effects:

- Regional temporal (random walk) hierarchical random-effects on the intercept
- Regional hierarchical random-effects for the coefficient associated with SAT
- Regional hierarchical random-effects for the coefficient associated with POP
- Smoothed, spatially varying, random-effects for the intercept
- Smoothed, spatially varying, random-effects for the coefficient associated with SAT

Due to both the complexity of the models and the size of the data, notably the number of spatial predictions that are required, recently developed techniques that perform "approximate" Bayesian inference based on integrated nested Laplace approximations (INLA) were used.[10] Computation was performed using the R interface to the INLA computational engine (R-INLA). For GBD 2019 and GBD 2021, the model also implements an innovative way to use samples from the (Bayesian) model to represent distributions of estimated concentrations in each grid cell. Estimates, and distributions

representing uncertainty, of concentrations for each grid cell are obtained by taking repeated (joint) samples from the posterior distributions of the parameters and calculating estimates based on a linear combination of those samples and the input variables.[11]

DIMAQ2 was used to produce grid-cell-level (0.1° × 0.1°) estimates of ambient $PM_{2.5}$ for 1990, 1995, and 2010–2020 by matching the gridded estimates with the corresponding coefficients from the calibration. For the year 2020, additional analysis was conducted to incorporate updated ground monitor (1777 observations for 2020) and satellite-based data (as described above) to examine potential impacts of the COVID-19 pandemic on ambient particulate matter pollution.

Model evaluation

Model development and comparison was performed using within- and out-of-sample assessment. For evaluation, cross-validation was performed using 25 combinations of training (80%) and validation (20%) datasets. Validation sets were obtained by taking a stratified random sample, using sampling probabilities based on the cross-tabulation of $PM_{2.5}$ categories (0–24.9, 25–49.9, 50–74.9, 75–99.9, 100+ $\mu g/m^3$) and super-regions, resulting in sets with the same distribution of $PM_{2.5}$ concentrations and super-regions as the overall set of sites. The following metrics were calculated for each training/validation set combination: for model fit—R^2; for predictive accuracy—root mean squared error (RMSE) and population-weighted root mean squared error (PwRMSE).

Evaluation of model results for GBD 2021 were comparable to those from GBD 2013 and GBD 2017 (the most recent model evaluation prior to GBD 2021). For GBD 2021, DIMAQ2 predictions of ground measurements in all super-regions produced a mean out-of-sample population-weighted RMSE of 8.50 (95% UI 6.17–12.77) $\mu g/m^3$ and an R^2 of 0.909 (0.886–0.926). The high-income super-region produced the most accurate predictions, with a mean population-weighted RMSE of 2.16 (2.09–2.23) $\mu g/m^3$, while south Asia produced the largest population-weighted mean RMSE, 31.56 (18.95– 51.88) $\mu g/m^3$. Trends in relative magnitude of PwRMSE are consistent with previous DIMAQ evaluations in GBD 2017 and 2019.

Figure 1: Summary measure of predictive ability, globally and by super-region. Points denote median values of out-of-sample population-weighted root mean square error ($\mu g/m^3$) from 25 validation sets. Vertical lines denote 95% uncertainty interval bounds.

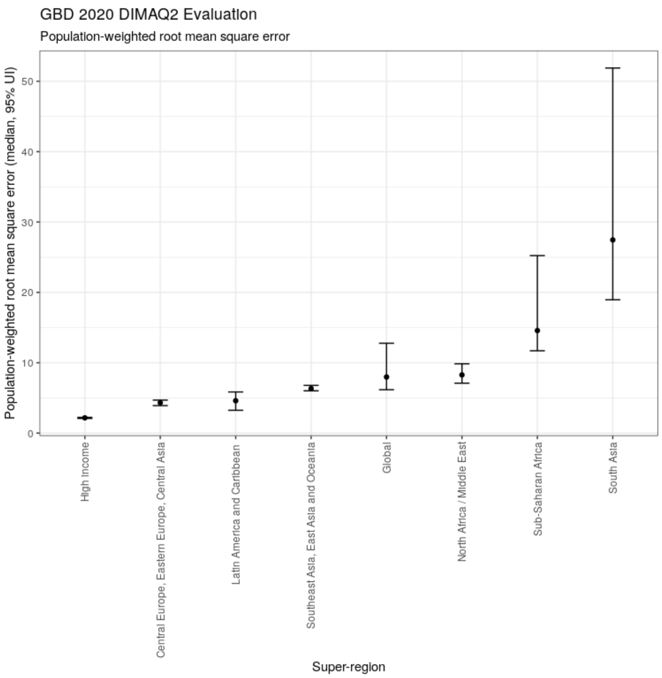

Table 2: Summary measure of predictive ability, globally and by super-region. Values denote median, lower, and upper 95% uncertainty interval bounds of out-of-sample population-weighted relative error (root mean square error/mean $PM_{2.5}$ prediction reported in μg/m^3) from 25 validation sets.

Location	Median	Lower	Upper
Global	0.115	0.105	0.133
Central Europe, eastern Europe, central Asia	0.189	0.180	0.199
High income	0.151	0.147	0.155
Latin America and Caribbean	0.234	0.179	0.313
North Africa and Middle East	0.243	0.217	0.263
South Asia	0.452	0.349	0.616
Southeast Asia, east Asia, and Oceania	0.174	0.169	0.184
Sub-Saharan Africa	0.322	0.256	0.409

Theoretical minimum-risk exposure level

The theoretical minimum-risk exposure level (TMREL) was assigned a uniform distribution with lower/upper bounds given by the average of the minimum and 5th percentiles of outdoor air pollution cohort studies exposure distributions conducted in North America, with the assumption that current evidence was insufficient to precisely characterise the shape of the concentration-response function below the 5th percentile of the exposure distributions. The TMREL was defined as a uniform distribution rather than a fixed value in order to represent the uncertainty regarding the level at which the scientific evidence was consistent with adverse effects of exposure. The specific outdoor air pollution cohort studies selected for this averaging were based on the criteria that their 5th percentiles were less than that of the American Cancer Society Cancer Prevention II (CPSII) cohort's 5th percentile of 8.2 based on Turner et al. (2016).[12] This criterion was selected because GBD 2010 used the minimum, 5.8, and 5th percentile solely from the CPS II cohort. The resulting lower/upper bounds of the distribution for GBD 2021 were 2.4 and 5.9. This has not changed since GBD 2015.

Relative risks and population attributable fractions

Input data

For GBD 2021, as in previous GBD cycles, we created one set of cause-specific risk curves for both household air pollution and ambient particulate matter pollution as two different sources of $PM_{2.5}$. In GBD 2017, we estimated the particulate-matter-attributable burden of disease based on the relation of long-term exposure to $PM_{2.5}$ with ischaemic heart disease, stroke (ischaemic and haemorrhagic), COPD, lung cancer, acute lower respiratory infection, and type 2 diabetes. In GBD 2019, we added adverse birth outcomes including low birthweight and short gestation as contributors to $PM_{2.5}$-attributable burden. Because these are risk factors (not outcomes) included in the GBD study, we performed a mediation analysis, in which a proportion of the burden attributable to low birthweight and short gestation is attributed to $PM_{2.5}$ pollution. For GBD 2021, as in previous cycles, we used risk curves to calculate burden for ages 25+ for ischaemic heart disease, stroke (ischaemic and haemorrhagic), COPD, lung cancer, and type 2 diabetes and for all ages for acute lower respiratory infection. Burden calculation for mediated outcomes is described below.

For the six non-mediated outcomes, we used results from cohort and case-control studies of ambient $PM_{2.5}$ pollution and cohort studies, case-control studies, and randomised-controlled trials of household use of solid fuel for cooking. For GBD 2021, we excluded secondhand smoke cohort and case-control studies from risk curve input data.

We conducted a literature review for studies of $PM_{2.5}$ (ambient and household air pollution) and risk of lower respiratory infection using the search string below. We searched the PubMed database for studies published between January 1, 2017, and July 22, 2020 (date of search). 32 initial results were obtained from the database, 31 of which were excluded during title-abstract and full-text screening. The remaining study was later excluded due to insufficient information reported on the study-specific exposure distribution.

Search string: ((("Air Pollution"[Mesh] OR "Particulate Matter"[Mesh] OR "air pollution"[Title/Abstract] OR "urban air pollution"[Title/Abstract] OR "ambient air pollution"[Title/Abstract] OR "airborne particulate matter"[Title/Abstract]) OR (“Air Pollution, Indoor”[Mesh] OR “Household air”[Title/Abstract] OR “Indoor air pollution”[Title/Abstract] OR “Indoor fine particulate

matter”[Title/Abstract] OR “Indoor particulate matter”[Title/Abstract] OR “Indoor air quality”[Title/Abstract])) AND ("lower respiratory infection"[Title/Abstract] OR "LRI"[Title/Abstract]))

Table 3: Data inputs for relative risks for ambient particulate matter pollution

Input data	Relative risk
Site-years (total)	196
Number of countries with data	53
Number of GBD regions with data (out of 21 regions)	18
Number of GBD super-regions with data (out of 7 super-regions)	7

For GBD 2021, as in GBD 2019, the meta-regression—Bayesian, regularised, trimmed (MR-BRT) meta-regression tool was used to create relative risk estimates, with three key updates to input data. In GBD 2017, we used relative estimates for active smoking and secondhand smoke (converting cigarettes per day to $PM_{2.5}$ exposure) to estimate relative risk predictions for $PM_{2.5}$ exposure at the highest end of the exposure–response curve. These data were included because the majority of the air pollution epidemiological studies have been performed in high-income countries which have lower levels of ambient $PM_{2.5}$ pollution. This posed a barrier to extrapolating relative risk estimates from the steep relationship at the beginning of the exposure range to locations with high exposures but no relative risk estimates, such as India and China. In GBD 2019, we incorporated estimates at high $PM_{2.5}$ levels by adding recently published ambient $PM_{2.5}$ studies conducted in China and other higher-exposure settings and additional HAP studies.[13–17]Additionally, the switch to MR-BRT splines in GBD 2019 (instead of the integrated exposure–response function employed in GBD 2017) presented a more flexible approach that allowed the curve to fit ambient and household data and removed the need for active smoking data to anchor the curve at higher exposures. The inclusion of active smoking and secondhand smoking data in previous GBD cycles required conversion from cigarettes per day to $PM_{2.5}$ exposure and introduced other differences, including differences in dose rates and those between voluntary (active smoking) and involuntary (ambient $PM_{2.5}$, household air pollution, secondhand smoke) exposures. Due to these factors, in GBD 2019, we removed active smoking data from the relative risk model’s input data. In GBD 2021, we also removed secondhand smoking data, completing the transition to only using $PM_{2.5}$ and HAP relative risk input data. This removes important sources of uncertainty in our earlier estimates.[18,19] The following plot displays $PM_{2.5}$ risk curves from GBD 2019 and from GBD 2021, with and without secondhand smoking RR input data:

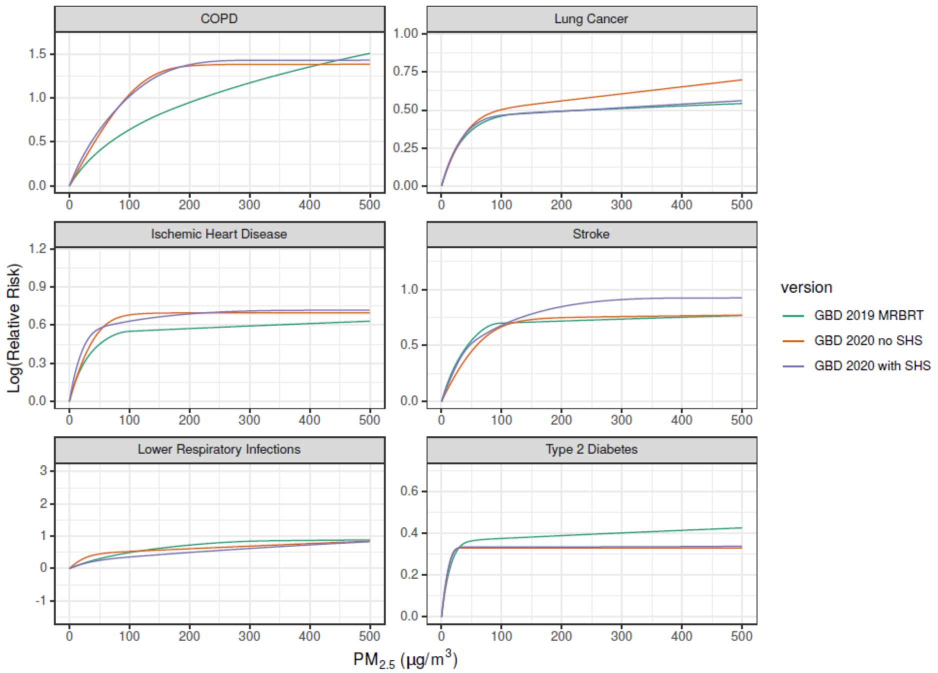

For GBD 2019, we implemented age-specific risk curves for cardiovascular diseases (ischaemic heart disease and stroke) due to evidence suggesting relative risk decreases with age for these outcomes.[20] These risk curves were created for five-year age groups from 25–29 to 95+. For GBD 2021, we dropped the use of age-specific risk curves for cardiovascular disease outcomes. Linear regressions on cardiovascular disease input data predicting log(RR) by mean cohort age, with and without random effects on study ID, were fit to ischaemic heart disease and stroke input data separately. None of these regressions showed evidence for a significant association between the two variables. Additionally, we used the MR-BRT automated covariate selection tool (detailed below) to test mean cohort age for significance as a bias covariate and found no significant results. We therefore generated a single risk curve for each of the cardiovascular outcomes and applied it across all age groups.

For all $PM_{2.5}$ outcomes, the standard error of observations from studies with multiple observations for a single cohort that reported an unstratified sample size were multiplied by the square root of *n*, where *n* is the total number of observations for a given cohort. This adjustment was made to prevent a single cohort or study from unduly weighting the final risk curve.

As in previous GBD cycles, we considered the published relative risk over a range of exposure data when fitting the risk curves. For OAP studies, the relative risk informs the curve from the 5th to the 95th percentile of observed exposure. When this is not available in the published study, we estimate the distribution from the provided information (mean and standard deviation, mean and IQR, etc.). We scale the RR to this range. For HAP studies, we allow each study to inform the curve from the Exp_{OAP} to the

Exp_{OAP}+Exp_{HAP}, where Exp_{OAP} is the GBD 2019 estimate of the ambient exposure level in the study location and year, and Exp_{HAP} is the GBD 2021 estimate of the excess exposure for those who use solid fuel for cooking in the study location and year.

MR-BRT risk splines

To estimate relative risk curves for each of the $PM_{2.5}$ outcomes, we used the MR-BRT meta-regression tool to fit splines on the input datasets of OAP and HAP studies. We used the following functional form, where X and X_{CF} represent the range of exposure characterised by the strength of association between exposure and outcome :

$$log\left(\frac{MRBRT(X)}{MRBRT(X_{CF})}\right) \sim \log\left(Published\ Effect\ Size\right)$$

Several key updates were made to the model fitting methods. For each risk–outcome pair, model settings and priors were tested when fitting the MR-BRT splines. The final models used third-order splines with three interior knots and a constraint on the right-most segment forcing the fit to be linear rather than cubic. Splines were also constrained to be concave and monotonically increasing, the most biologically plausible shape for the $PM_{2.5}$ risk curve. We used an ensemble approach to generate final spline predictions, in which 50 different models were run with randomly placed knots, then weighted and combined based on a measure of fit that penalises excessive changes in the maximum derivative of the curve. Knots were free to be placed across the entire domain of the input exposure data. To prevent over-fitting, on the non-linear segments, we implemented a Gaussian prior on the third derivative of mean 0 and variance 1e-4. On the linear segment, a stronger prior of mean 0 and variance 1e-6 was used to ensure that the risk curves do not continue to increase beyond the range of the data. 10% of all observations were trimmed during model fitting, in accordance with GBD protocol across risk factor teams.

To select significant covariates from those extracted (see table below) to quantify between-study heterogeneity, we performed covariate selection. The MR-BRT automated covariate selection tool implements a two-step process. First, a series of loosening Lasso penalty parameters are applied to a log-linear meta-regression on all input strength of association between exposure and outcome observations. Then, covariates with a non-zero coefficient are tested for significance using a Gaussian prior (significance threshold = 0.05). A Gaussian prior was used on each covariate's beta during spline fitting with a mean 0 and variance of 0.1 multiplied by the standard deviation of the beta from the log-linear meta-regression. Type 2 diabetes was the only outcome for which a significant covariate was identified. Its selected covariate was cv_hap, a binary indicator for whether or not an observation was from a household air pollution study.

**Covariate name**	**Covariate description**
cv_subpopulation	Study represents the general population; study represents a subgroup (eg, high-risk group)
cv_exposure_population	Study measures individual-level exposure (≤1 km radius); study measures population-level exposure
cv_exposure_self_report	Exposure is self-reported; exposure is measured externally
cv_exposure_study	Exposure is measured multiple times; exposure is measured only at baseline

cv_outcome_self_report	Outcome is self-reported; outcomes is based on death certificate or medical record
cv_outcome_unblinded	Study implements unblinded assessment; assessment of outcome is blind to exposure (and vice versa)
cv_reverse_causation	Study presents no risk of reverse causation; risk of reverse causation
cv_confounding_nonrandom	Non-randomised study; randomised study
cv_confounding_uncontrolled	Study is randomised/outcome controlled for age, sex, education, income, and all critical determinants of outcome; study is controlled for age, sex, and other critical determinants of outcome; study is controlled for only age and sex
cv_selection_bias	Study reports >95% follow-up; study reports 85–95% follow-up; study reports <85% follow-up
cv_hap	Studies household air pollution; studies ambient air pollution

1000 predictions of the strength of association between exposure and outcome were generated across the exposure distribution for use in calculating burden estimates. These predictions were created by incorporating predictions of between-study heterogeneity to characterise the model's uncertainty. We implemented the Fisher scoring correction to the heterogeneity parameter, which corrects for data-sparse situations. In such cases, the between-study heterogeneity parameter estimate may be 0, simply from lack of data. The Fisher scoring correction uses a quantile of gamma, which is sensitive to the number of studies, study design, and reported uncertainty.

Risk-outcome scoring

Risk-outcome scores provide an empirical measure of the strength of evidence for risk-outcome pairs across risk factors in the GBD and are therefore useful for standardised comparison. Risk-outcome scores evaluate the area between the lower bound of the 95% uncertainty interval and the x-axis for harmful risk factors, including $PM_{2.5}$ pollution.

Prior to generating a risk-outcome score, we conducted an additional post-analysis step to detect and flag publication bias in the input data. This approach is based on the classic Egger's regression strategy, which is applied to the residuals in our model. In the current implementation, we do not correct for publication bias, but flag the risk–outcome pairs where the risk for publication bias is significant. Of the $PM_{2.5}$ outcomes, three were flagged for publication bias: birthweight, ischaemic heart disease, and type 2 diabetes.

Outcome	Egger p-value	Egger mean	Egger SD	Publication bias
Birthweight	0.0208	–0.322	0.158	X
Gestational age	0.249	–0.130	0.192	
Ischaemic heart disease	0.0164	0.322	0.151	X
Stroke	0.0717	0.186	0.127	

LRI	0.178	0.102	0.110	
Lung cancer	0.191	0.108	0.123	
COPD	0.423	0.0359	0.186	
Type 2 diabetes	0.0419	0.408	0.236	X

To calculate the risk-outcome score, we generated an uncertainty interval from 1000 draws of the adjusted summary strength of association between exposure and outcome (retaining uncertainty information from between-study heterogeneity predictions and the Fisher information correction). We then evaluated the risk-outcome score between the 15th and 85th percentiles of the input data exposure distribution. Risk-outcome scores and star ratings are below. Risk-outcome scores are not reported for birthweight and gestational age because these are mediated outcomes.

**Outcome**	**Risk-outcome score**	**Star rating**
Ischaemic heart disease	0.259	3
Stroke	0.167	3
LRI	0.126	2
Lung cancer	0.342	3
COPD	0.441	4
Type 2 diabetes	0.188	3

The following table includes all ambient and household sources used to generate GBD 2021 risk curves.

The following figures display risk curves for each outcome. The dashed line depicts the GBD 2017 IER including active smoking data, the dotted line depicts the GBD 2019 MR-BRT curve without active smoking but with secondhand smoking data, and the solid line depicts the GBD 2021 MR-BRT curve without the inclusion of active smoking or secondhand smoking data. For GBD 2021, a single curve is used for cardiovascular diseases (ischaemic heart disease, stroke) for all ages, so only one plot is displayed for each of these outcomes. For the GBD 2017 and GBD 2021 curves, the curve for the age group 60–64 is plotted for the cardiovascular disease outcomes because these cycles used age-specific cardiovascular disease curves. For birthweight and gestational age, no curve is displayed for GBD 2017 because these outcomes were added to the GBD in the 2019 cycle. The grey shaded areas represent the 95% CI. The red box represents the TMREL area of the curve. On each page, the first figure depicts the typical range of outdoor exposure, whereas the second plot includes higher levels typical of household air pollution exposure.

Each point or number represents one study effect size. Each is plotted at the 95th percentile of the exposure distribution (OAP) or the expected level of exposure for individual using solid fuel (HAP). The relative risk is plotted relative to the predicted relative risk at the 5th percentile of exposure distribution (OAP) or the expected (ambient only) level of exposure for individuals not using solid fuel (HAP). For

example, a study predicting a relative risk of 1.5 for an exposure range of 10 to 20 would be plotted at (20, MRBRT(10)*1.5). Arrows represent studies that would have been outside the range of the plot but have been shifted to be included in the figure.

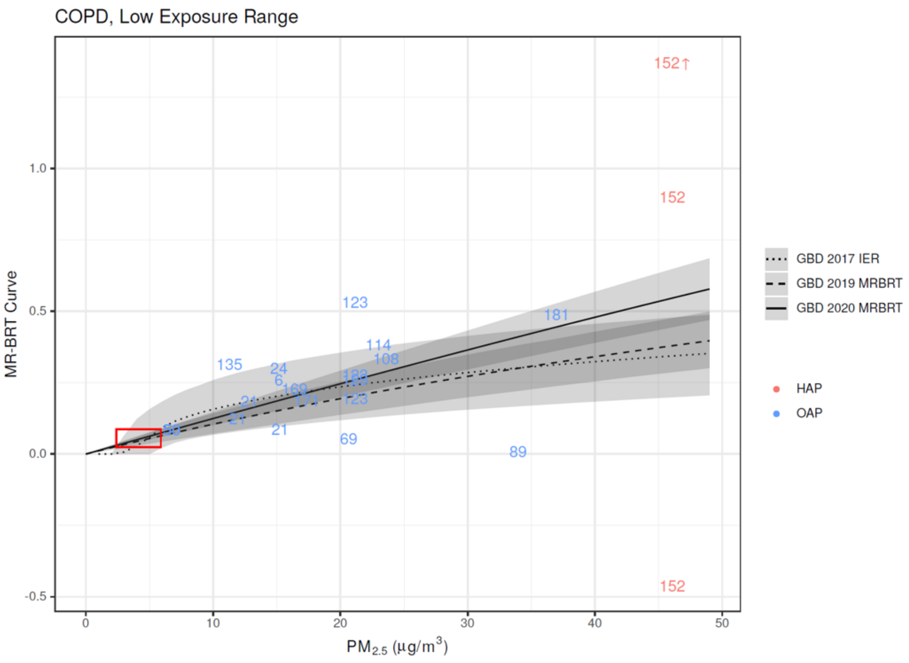
COPD, Low Exposure Range
MR-BRT Curve
PM2.5 (µg/m3)
GBD 2017 IER
GBD 2019 MRBRT
GBD 2020 MRBRT
HAP
OAP

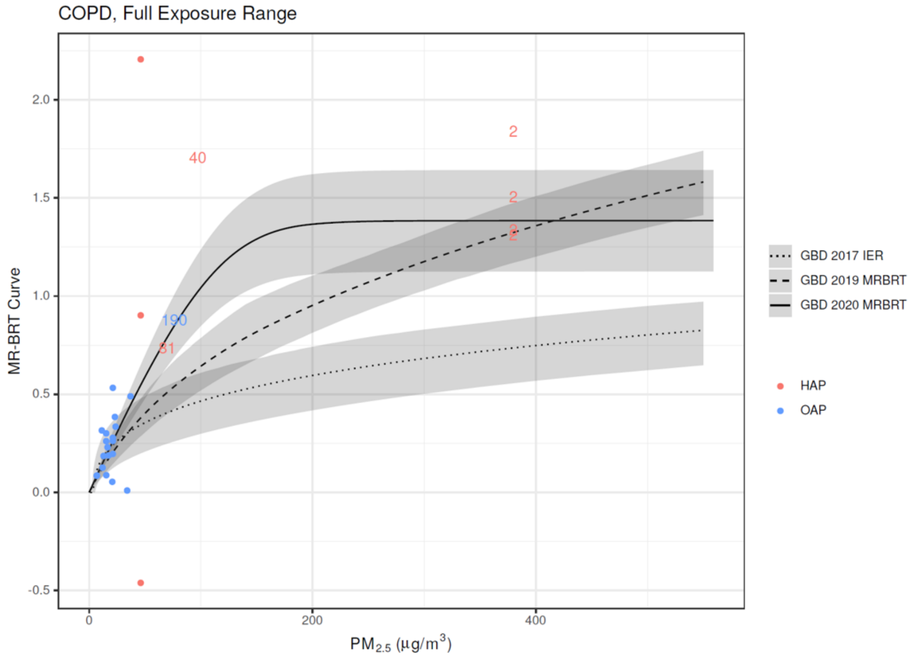
COPD, Full Exposure Range
MR-BRT Curve
PM2.5 (µg/m3)
GBD 2017 IER
GBD 2019 MRBRT
GBD 2020 MRBRT
HAP
OAP

Lower Respiratory Infections, Low Exposure Range

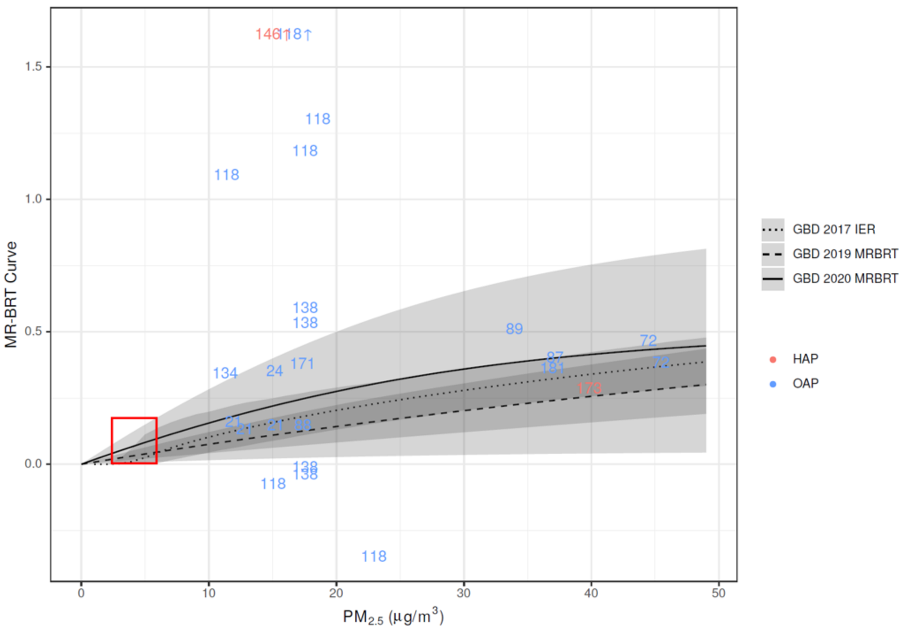

Lower Respiratory Infections, Full Exposure Range

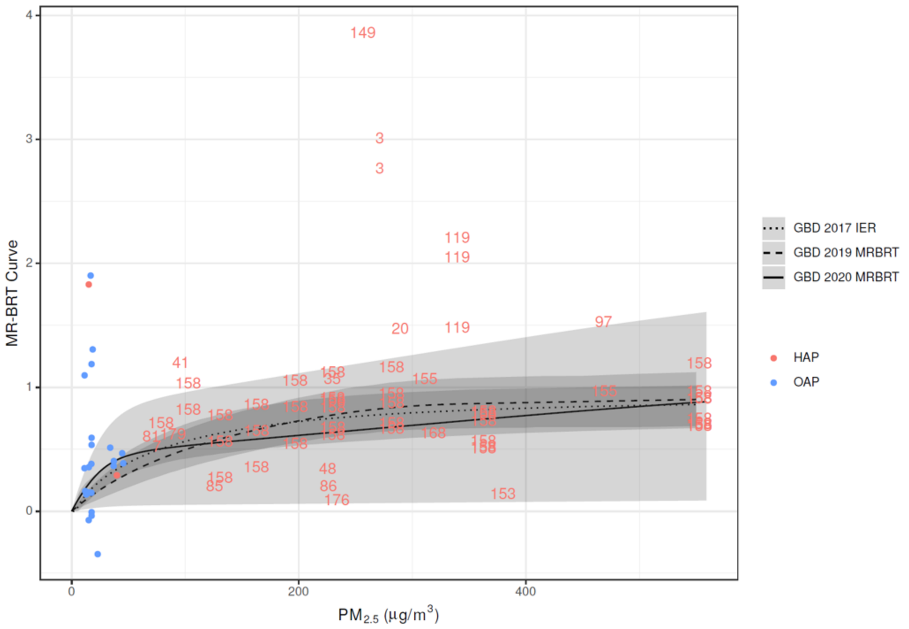

Lung Cancer, Low Exposure Range

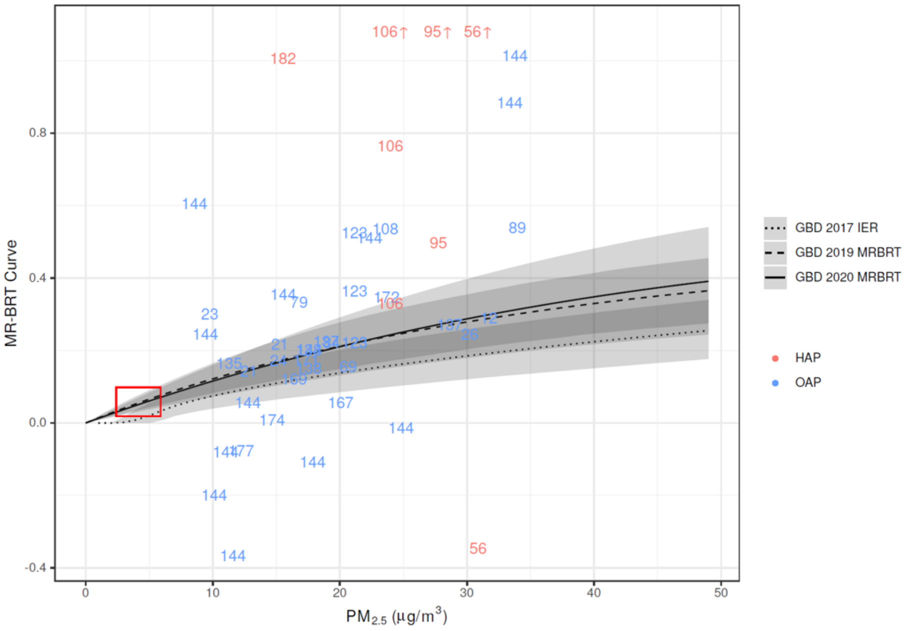

Lung Cancer, Full Exposure Range

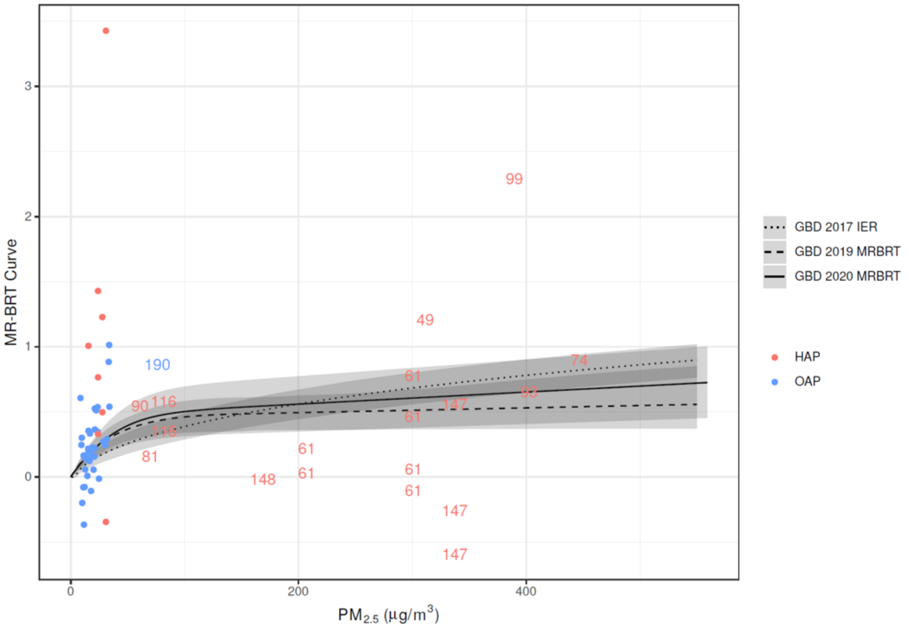

Type 2 Diabetes, Low Exposure Range

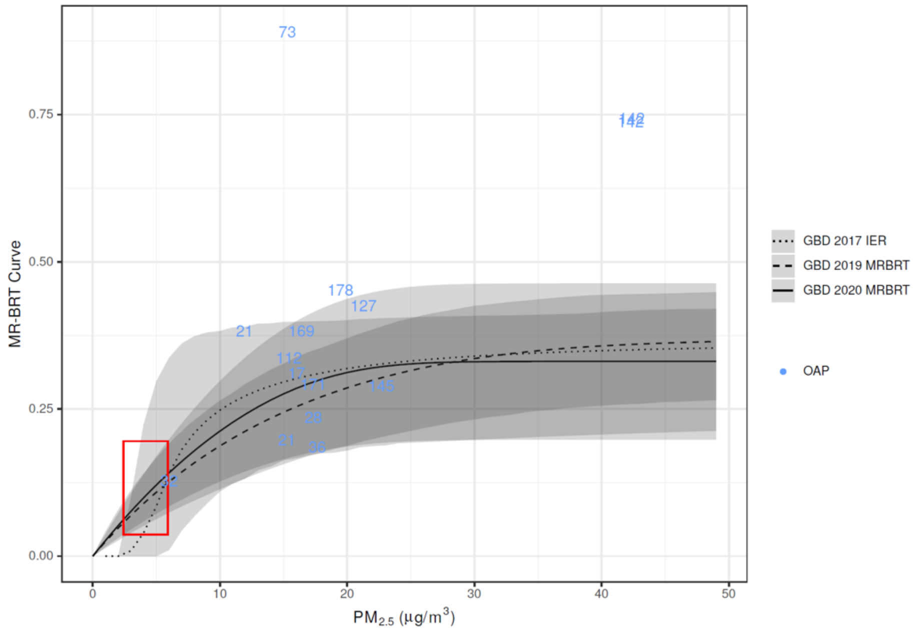

Type 2 Diabetes, Full Exposure Range

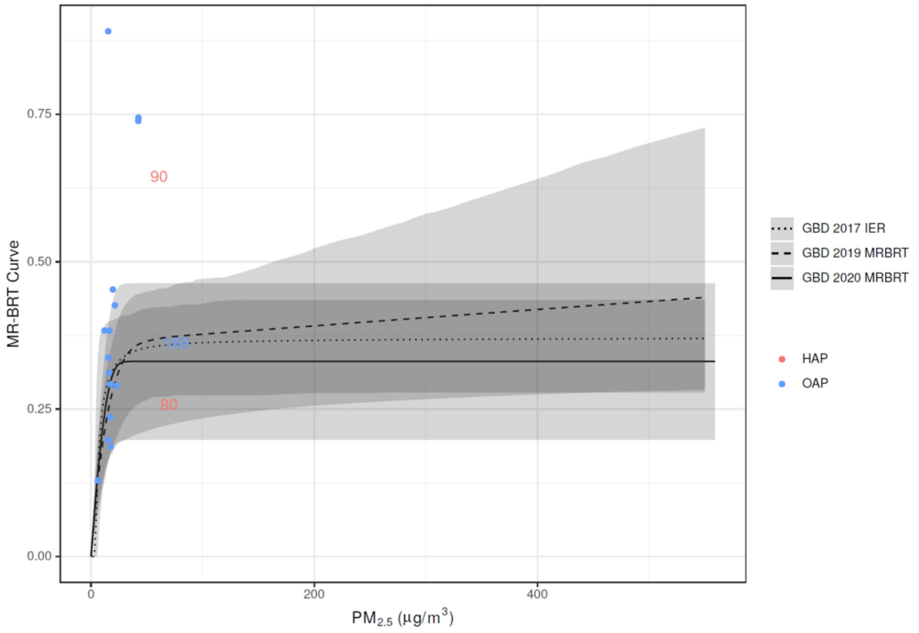

Ischemic Heart Disease, Low Exposure Range

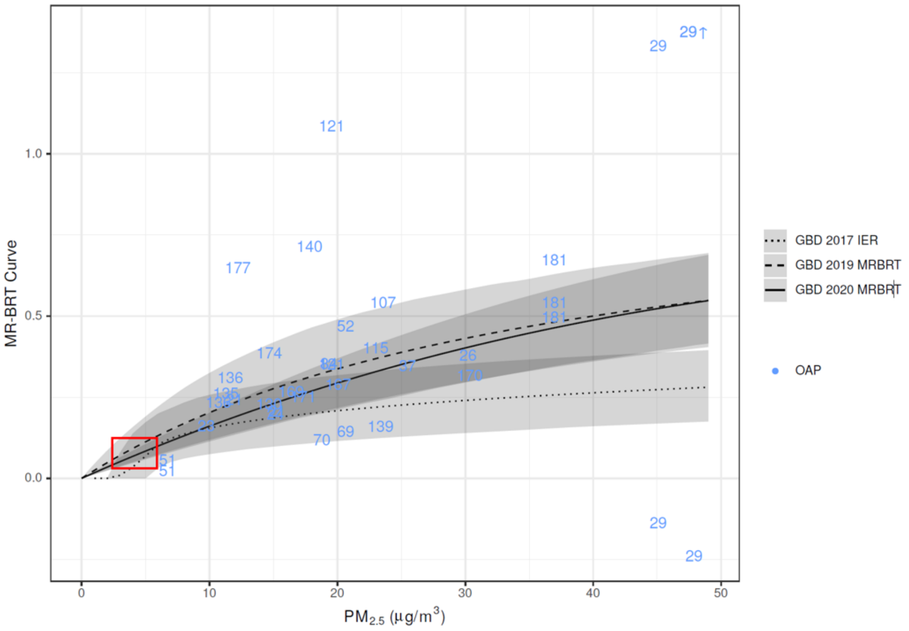

Ischemic Heart Disease, Full Exposure Range

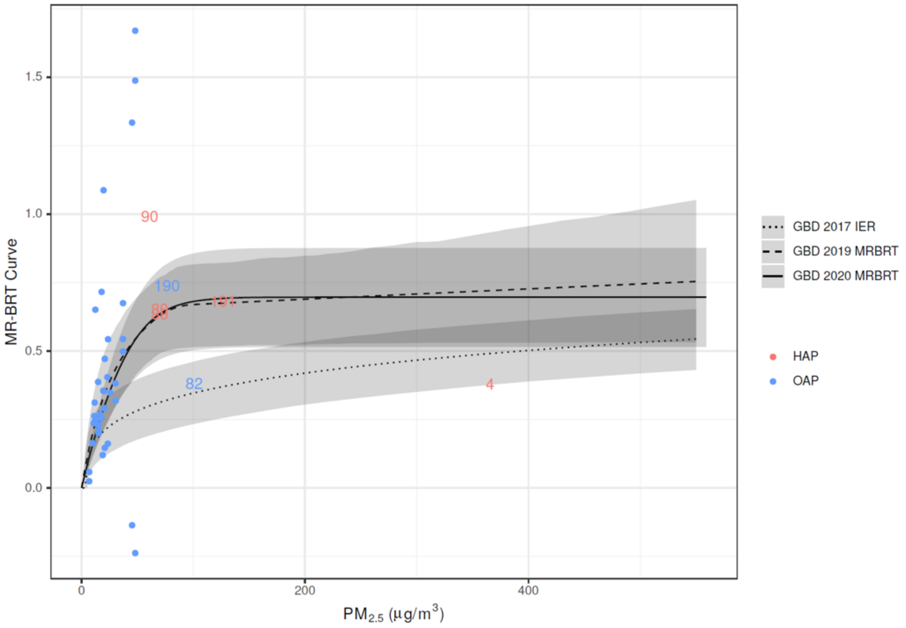

Stroke, Low Exposure Range

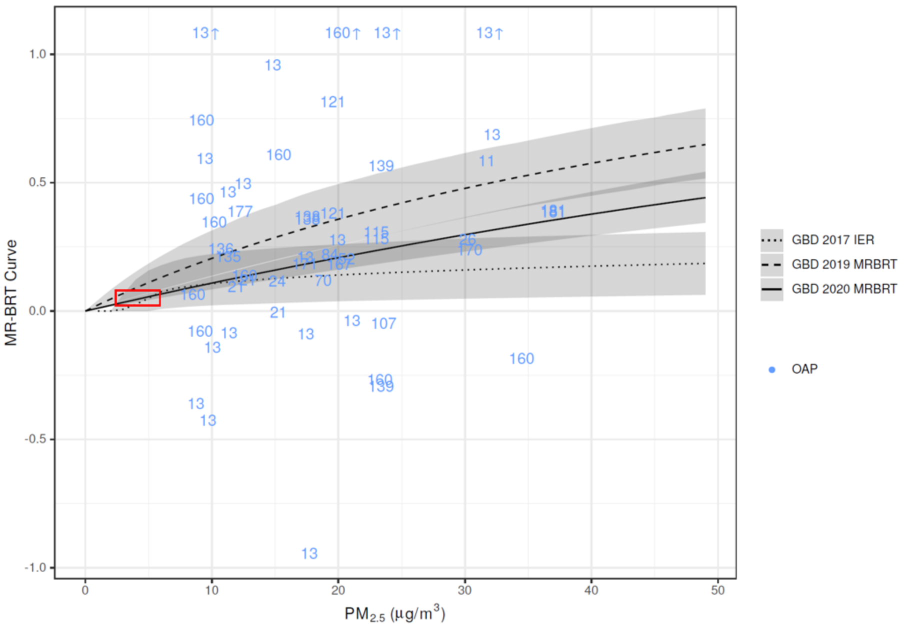

Stroke, Full Exposure Range

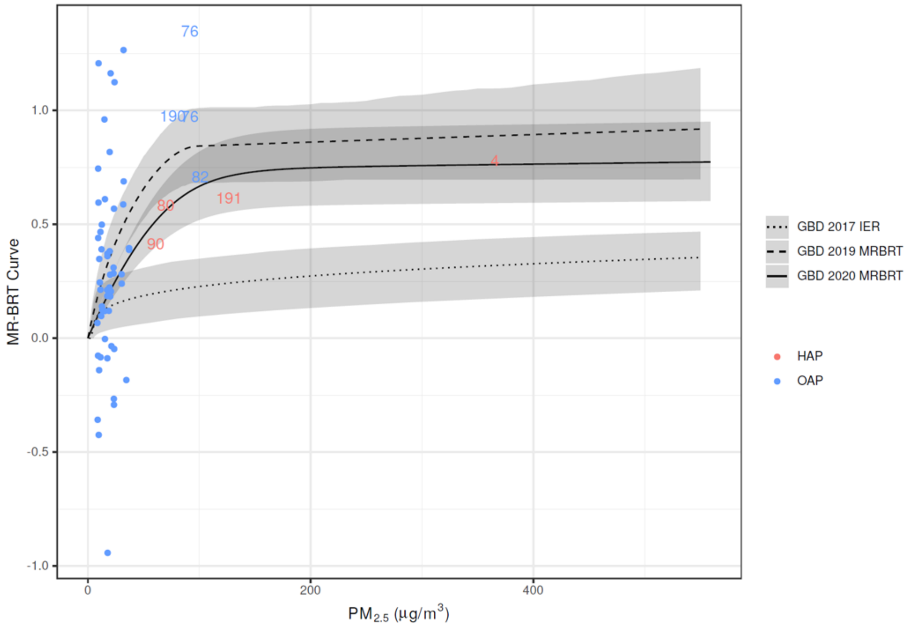

Low birthweight and short gestation mediation analysis

As in GBD 2019, in GBD 2021, low birthweight and short gestation were included as $PM_{2.5}$ outcomes via a mediation analysis. Low birthweight and short gestation includes mortality due to diarrhoeal diseases, lower respiratory infections, upper respiratory infections, otitis media, meningitis, encephalitis, neonatal preterm birth, neonatal encephalopathy due to birth asphyxia and trauma, neonatal sepsis and other neonatal infections, haemolytic disease and other neonatal jaundice, and other neonatal disorders. Morbidity estimates were also calculated for neonatal preterm birth. These outcomes are specific to the neonatal ages: 0–6 days and 7–27 days.

The following is a summary of methods used to conduct the mediation analysis. For GBD 2019, we conducted a systematic review of all cohort, case-control, or randomised-controlled trial studies of ambient $PM_{2.5}$ pollution or household air pollution and birthweight or gestational age outcomes for GBD 2019.[21] Outcomes measured included continuous birthweight (bw), continuous gestational age (ga), low birthweight (LBW) (<2500 g), preterm birth (PTB) (<37 weeks), and very preterm birth (VPTB) (<32 weeks). We included any papers published until April 4, 2021.

Birthweight and gestational age are modelled using a continuous joint distribution for the GBD. To determine how these distributions are influenced by $PM_{2.5}$ pollution, we used available literature to model the continuous shift in birthweight (bw, grams) and gestational age (ga, weeks) at a given $PM_{2.5}$ exposure level. When available, we used estimates of continuous shifts in bw or ga directly from each study. When shifts were not available, we converted the published OR/RR/HR for LBW, PTB, or VPTB using the following strategy:

1. Extract the OR/RR/HR from the study.
2. Select the GBD 2017 estimated bw-ga joint distribution for the study location and year.
3. Calculate the number of grams or weeks required to shift the distribution such that the proportion of births under the specified threshold (P) is reduced by the study effect size to a counterfactual level (P_{cf}).
4. Save the resulting shift and 95% CI as the continuous effect.

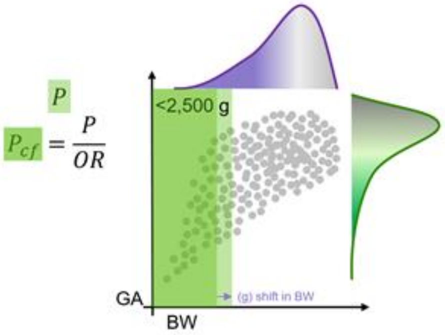

When preparing HAP data to fit splines, we used the same strategy described above for other outcomes to map HAP input data to $PM_{2.5}$ exposure values. We then fit MR-BRT splines to the input studies, where the difference in the value of the model at the upper concentration (X) and the value of the model at the counterfactual concentration (X_{CF}) is equal to the published or calculated shift in bw or ga:

$$MRBRT(X) - MRBRT(X_{CF}) \sim Shift$$

We used the same model fitting process, settings, and covariate selection process as described above for the other outcomes. The only exception is that, because the change in birthweight and gestational age was expected to be negative, the splines were constrained to be monotonically decreasing.

The following figures display MR-BRT curves for linear shift in grams (bw) and weeks (ga).

Birthweight (g), Low Exposure Range

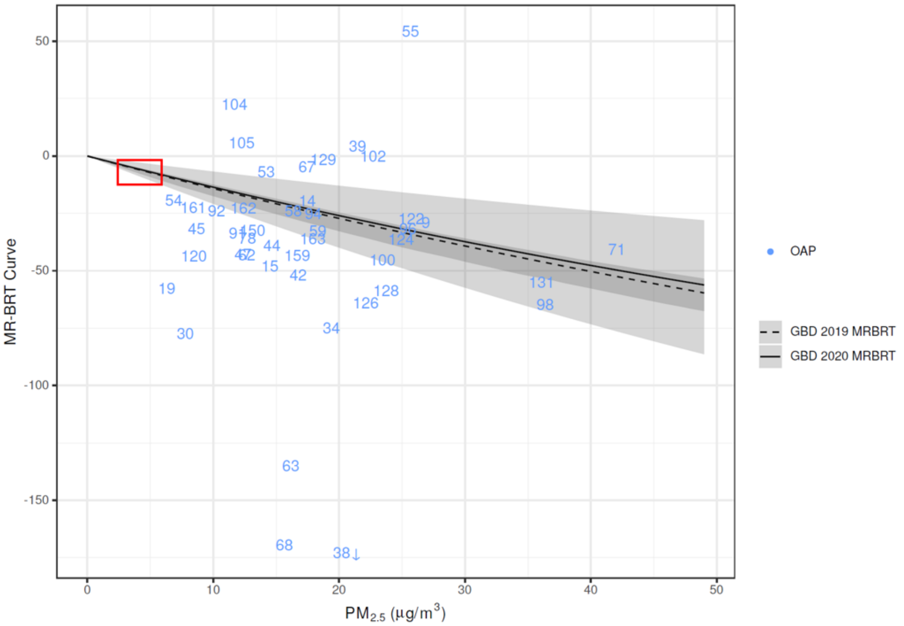

Birthweight (g), Full Exposure Range

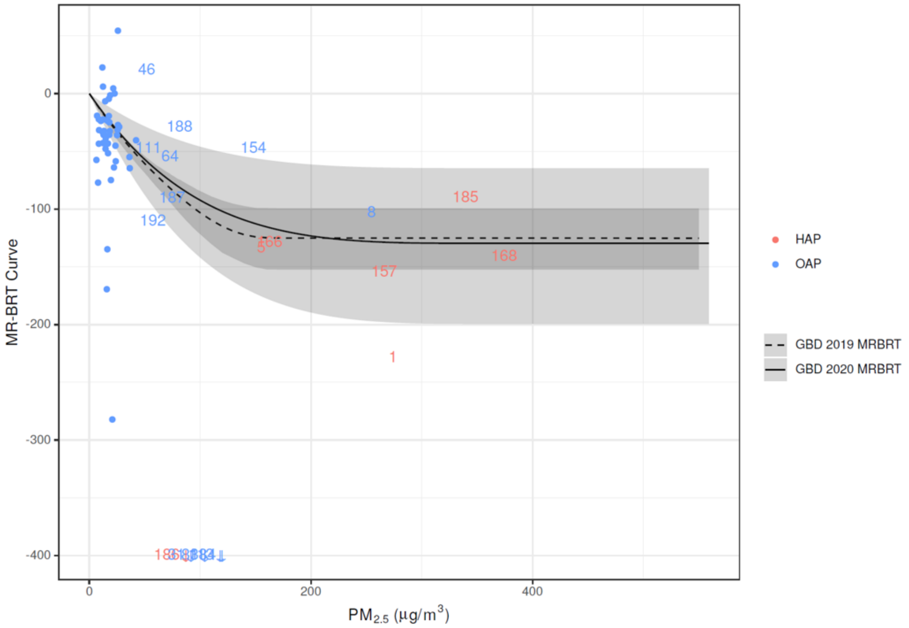

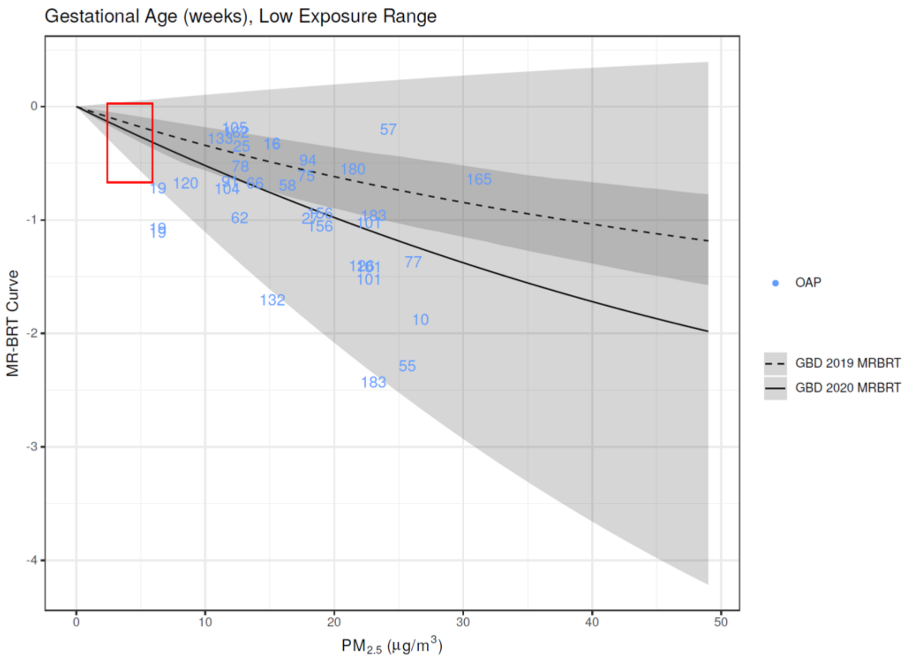
Gestational Age (weeks), Low Exposure Range
MR-BRT Curve
PM2.5 (µg/m3)
OAP
GBD 2019 MRBRT
GBD 2020 MRBRT

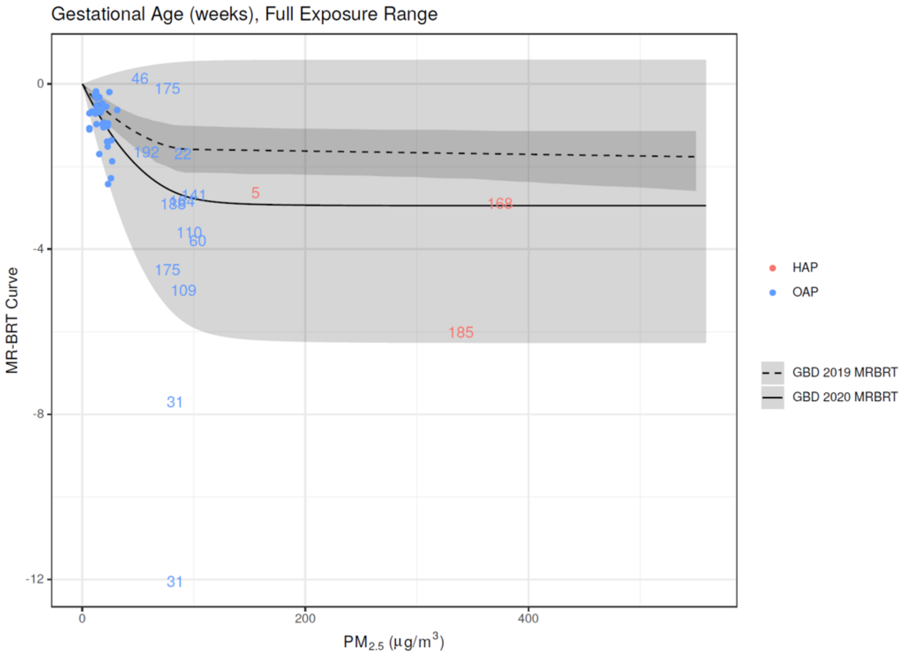
Gestational Age (weeks), Full Exposure Range
MR-BRT Curve
PM2.5 (µg/m3)
HAP
OAP
GBD 2019 MRBRT
GBD 2020 MRBRT

We used the curves of estimated shifts across the exposure range to predict the shift in both birthweight and gestational age for total female particulate matter pollution exposure in each location and year. Because the epidemiological studies mutually controlled for birthweight and gestational age, we assumed these shifts are independent. We then shifted the observed distributions to reflect the expected bwga distribution in the absence of particulate matter pollution. These shifted distributions were used as the counterfactual in the PAF calculation equation to calculate the burden attributable to $PM_{2.5}$ pollution.

To calculate PAFs, the distribution is divided into 56 bw-ga categories, each with a unique RR. Let p_i be the observed proportion of babies in category, *i* and p_i' be the counterfactual proportion of babies in category, *i* if there were no particulate matter pollution.

$$PAF_{PM} = \frac{\sum_{i \in bwga\ category} RR_i p_i - \sum_{i \in bwga\ category} RR_i p_i'}{\sum_{i \in bwga} RR_i p_i}$$

We proportionately split this PAF to ambient and HAP based on exposure as described below. One important assumption to note is that we assume the shift in bw and ga is linear across the bwga distribution.

For lower respiratory infections, $PM_{2.5}$-attributable PAFs are directly estimated in addition to estimated through bwga mediation. We expect that some of the directly estimated PAFs are mediated through bw and ga. Additionally, the directly estimated PAF is based on a summary of relative risks for all children under 5 years, so there is a possibility that the mediated PAF, which is more finely resolved, could be greater. To avoid double counting, for the two neonatal age groups (0–6 days and 0–27 days), we take the maximum of the two PAF estimates. If the directly estimated PAF is greater than the bwga-mediated PAF, we take the direct estimate, and if the mediated PAF is greater, we take the mediated estimate.

PTB incidence and mortality are both outcomes measured in the GBD. 100% of the burden for this cause is attributable to short gestation. To calculate the percentage attributable to particulate matter pollution, we estimated the percentage of babies born at less than 37 weeks (p_{ptb}) and the percentage of babies that would have been born at less than 37 weeks in the counterfactual scenario of no particulate matter pollution (p_{ptb}').

$$PAF_{ptb,pm} = 1 - \frac{p_{ptb}'}{p_{ptb}}$$

Limitations

Although for GBD 2021 we have not used active smoking or secondhand smoking data to estimate $PM_{2.5}$ risk curves, we still use an integrated exposure–response approach because we integrate relative risk estimates across ambient and HAP sources. The use of both source types to construct a risk curve with $PM_{2.5}$ as the exposure indicator assumes equitoxicity of particles regardless of source, despite evidence suggesting differences in health impacts by specific PM source (eg, motor vehicles, coal-fired power plant), size, and/or chemical composition. However, in the absence of sufficient estimates of source- or composition-specific exposure–response relationships and consistent and robust evidence of differential toxicity by source, integrating across all OAP and HAP studies is the approach most consistent with the current evidence, as reviewed by USA EPA and WHO.[22,23]

Proportional PAF approach

Prior to GBD 2017, relative risks for both ambient and HAP exposures were obtained from the risk curve as a function of exposure, relative to the same TMREL. In reality, were a country to reduce only one of these risk factors, the other would remain. We did not consider the joint effects of particulate matter from outdoor exposure and burning solid fuels for cooking. For GBD 2017, we developed a new approach to use the risk curve for obtaining PAFs for both OAP and HAP, which was also implemented in GBD 2019 and 2021.

Let Exp_{OAP} be the ambient $PM_{2.5}$ exposure level and Exp_{HAP} be the excess exposure for those who use solid fuel for cooking. Let P_{HAP} be the proportion of the population using solid fuel for cooking. We calculated PAFs at each 0.1° × 0.1° grid cell. We assumed that the distribution of those using solid fuel for cooking (HAP) was equivalent across all grid cells of the GBD location.

For the proportion of the population not exposed to HAP the relative risk was:

$$RR_{OAP} = MRBRT(z = Exp_{OAP})/MRBRT(z = TMREL),$$

And for those exposed to HAP, the relative risk was

$$RR_{HAP} = MRBRT(z = Exp_{OAP} + Exp_{HAP})/MRBRT(z = TMREL).$$

We then calculate a population-level RR and PAF for all particulate matter exposure:

$$RR_{PM} = RR_{OAP}(1 - P_{HAP}) + RR_{HAP}P_{HAP}$$

$$PAF_{PM} = \frac{RR_{PM} - 1}{RR_{PM}}$$

We population weight the grid-cell level particulate matter PAFs to get a country-level PAF, and finally, we split this PAF based on the average exposure to each OAP and HAP:

$$PAF_{OAP} = \frac{Exp_{OAP}}{Exp_{OAP} + P_{HAP} * Exp_{HAP}} PAF_{PM}, \text{ and } PAF_{HAP} = \frac{P_{HAP} * Exp_{HAP}}{Exp_{OAP} + P_{HAP} * Exp_{HAP}} PAF_{PM}.$$

With this strategy, $PAF_{PM} = PAF_{HAP} + PAF_{OAP}$, and no burden is counted twice.

Household air pollution

Appendix Figure 7: Flowchart of household air pollution

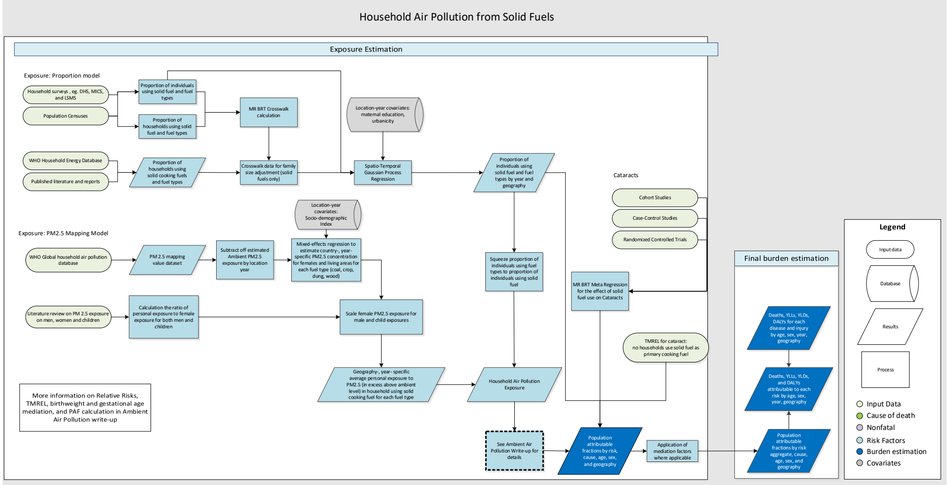

Input data and methodological summary

Exposure

Definition

Exposure to household air pollution from solid fuels (HAP) is estimated from both the proportion of individuals using solid cooking fuels and the level of exposure to particulate matter less than 2.5 micrometers in diameter ($PM_{2.5}$) air pollution for these individuals. Solid fuels in our analysis include wood, coal/charcoal, dung, and agricultural residues.

Input data

We extracted information on the use of solid fuels for cooking from standard multi-country survey series, including the Demographic and Health Surveys (DHS), Living Standards Measurement Surveys (LSMS), Multiple Indicator Cluster Surveys (MICS), and World Health Surveys (WHS). We also used data from censuses and country-specific survey series, such as the Kenya Welfare Monitoring Survey and South Africa General Household Survey. To fill remaining gaps in survey and census data, we downloaded the WHO Household Energy Database and updated estimates using extracted information from literature through a systematic review.[24] From this combined body of input data, each nationally or subnationally representative datapoint provided an estimate of the percentage of households or individuals using solid cooking fuels. We used studies from 1980 to 2020 to inform our time series estimates.

We excluded sources that did not distinguish specific primary fuel types, estimated fuel used for purposes other than cooking (eg, lighting or heating), failed to report standard error or sample size, reported over 15% missingness for households surveyed, reported fuel use in physical units, or were secondary sources referencing primary analyses.

Appendix Table 11: Data inputs for exposure for household air pollution.

Input data	Exposure
Site-years (total)	1173
Number of countries with data	161
Number of GBD regions with data (out of 21 regions)	20
Number of GBD super-regions with data (out of 7 super-regions)	7

Family size crosswalk

Many estimates in the WHO Energy Database and other reports quantify the proportion of households using solid fuel for cooking; however, we are interested in the proportion of individuals using solid fuel for cooking for exposure and burden assessment. To crosswalk these estimates, where available, we extracted fuel use at both the individual and household levels. We used studies that reported values for both household and individual solid fuel use and did not report a mean of 0 or 1. This resulted in 8074 source-specific pairs used as input data for the crosswalk model, which was modelled with the meta-regression—Bayesian, regularised, trimmed (MR-BRT) meta-regression tool. We applied this crosswalk only to proportion estimates for the parent solid fuel category. We did not adjust fuel-specific (coal/charcoal, crop, dung, or wood) proportion estimates due to lack of sufficient data for each individual fuel type.

Appendix Table 12: MR-BRT crosswalk adjustment factors for household air pollution exposure

**Data input**	**Reference or alternative case definition**	**Gamma**	**Beta coefficient, logit (95% UI)***	**Adjustment factor (95% UI)****
Proportion of individuals	Ref	0.095	---	---
Proportion of households	Alt		-0.094 (-0.097, -0.090)	1.099 (1.094–1.102)

MR-BRT crosswalk adjustments can be interpreted as the factor the alternative case definition is adjusted by to reflect what it would have been had it been measured using the reference case definition. If the log/logit beta coefficient is negative, then the alternative is adjusted up to the reference. If the log/logit beta coefficient is positive, then the alternative is adjusted down to the reference.

*****The adjustment factor column is the exponentiated negative beta coefficient. For log beta coefficients, this is the relative rate between the two case definitions. For logit beta coefficients, this is the relative odds between the two case definitions.***

We applied this coefficient to household-only solid fuel reports with the following formula:

$prop_{individ}$ = the proportion of individuals using solid fuel for cooking, and

$prop_{hh}$ = the proportion of households using solid fuel for cooking.

$$\log\left(\frac{prop_{individ}}{1 - prop_{individ}}\right) = \log\left(\frac{prop_{hh}}{1 - prop_{hh}}\right) - \beta$$

or

$$prop_{individ} = \frac{prop_{hh} * e^{-\beta}}{1 - prop_{hh} + prop_{hh} * e^{-\beta}}$$

As a result, household studies were inflated to account for bias in size between households that use solid cooking fuels and those that do not. Larger households are more likely to use solid fuels for cooking. The following figure depicts the 8074 datapoints that informed the crosswalk model. Red points indicate the 10% of studies trimmed as outliers during model fitting.

Appendix Figure 8: MR-BRT crosswalk for household air pollution exposure

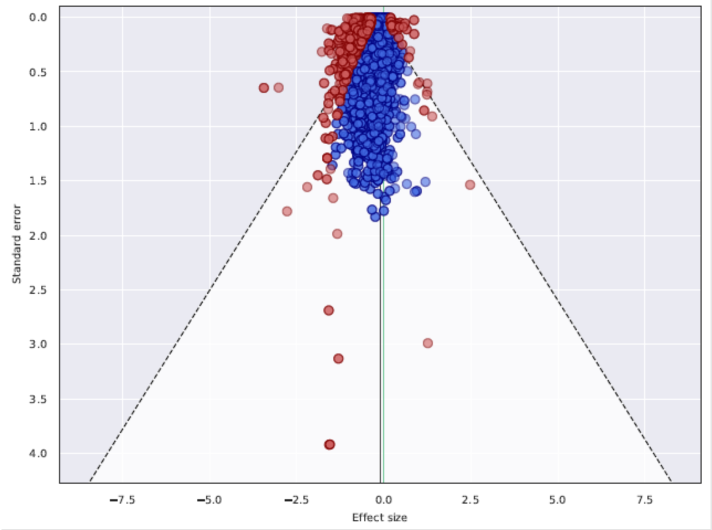

$logit(prop_{hh}) - logit(prop_{individual})$

Modelling strategy

As in the Global Burden of Disease (GBD) Study 2019, household air pollution was modelled at the individual level using a three-step modelling strategy implementing linear regression, spatiotemporal regression, and Gaussian process regression (GPR). The full ST-GPR process is specified elsewhere in this appendix.

For GBD 2021, we updated the HAP proportion model to disaggregate estimates of solid fuel use to estimate the proportion of individuals using each of the following component fuel type categories: 1) coal or charcoal, 2) crop residue, 3) dung, and 4) wood. With this strategy, we can more finely

characterise individual exposure to $PM_{2.5}$ due to solid fuel use by applying fuel-specific mapping values to fuel-specific proportion estimates. This change addresses an important limitation in our model, in that it previously assumed equal $PM_{2.5}$ exposure for all solid fuel categories.

Fuel type-specific estimates were generated by first using ST-GPR to generate location- and year-specific estimates for coal, crop, dung, and wood. ST-GPR was also used to create estimates for the parent solid fuel category, as in GBD 2019. The first step of the ST-GPR modelling process is a mixed-effect linear regression of logit-transformed proportion of individuals using solid cooking fuels. For each of the linear models, maternal education and the proportion of population living in urban areas were used as covariates. These models also included nested random effects by GBD region and GBD super-region.

Appendix Table 13: First-stage linear model and coefficients (solid model)

**Variable**	**Beta (95% UI)**
Intercept	3.36 (2.01, 4.71)
Maternal education (years per capita)	-0.55 (-0.58, -0.51)
Urbanicity (proportion of population living in urban areas)	-0.14 (-0.67, 0.39)

The four fuel-type-specific proportion estimates were then squeezed to the estimates for the overall proportion of individuals using solid fuel for cooking. For each location and year, we used the following formula, where $prop_{coal}$, $prop_{crop}$, $prop_{dung,}$ $prop_{wood}$, and $prop_{solid}$ indicate the proportion of individuals using coal, crop, dung, wood, or any type of solid fuel, respectively.

$$\text{Let } prop_{total} = prop_{coal} + prop_{wood} + prop_{crop} + prop_{dung}$$

$$S = prop_{total} \,/\, prop_{solid}$$

For each fuel category, with coal shown below as an example, the adjusted (squeezed) proportion is calculated as

$$prop_{coal}{}' = prop_{coal} \,/\, S$$

In preliminary model iterations, we mapped mixed fuel strings (eg, “wood and agricultural residues") to the category associated with highest $PM_{2.5}$ exposure to avoid underestimating HAP exposure. However, fuel-specific ST-GPR models were unstable with this approach. We therefore excluded mixed-fuel string studies from final estimates for fuel-specific proportions, though we retained these studies when modelling the proportion of overall solid fuel use.

Theoretical minimum-risk exposure level

For all HAP outcomes except cataract, burden is related to both ambient and household air pollution. These PAFs are estimated jointly and the theoretical minimum-risk exposure level (TMREL) is defined as a uniform distribution between 2.4 and 5.9 μg/m^3 $PM_{2.5}$. For cataract, the TMREL is defined as no individuals using solid cooking fuel.

Relative risks

The outcomes associated with household air pollution are lower respiratory infections (LRI), stroke, ischaemic heart disease (IHD), chronic obstructive pulmonary disease (COPD), lung cancer, type 2 diabetes, and cataract. Low birthweight and short gestation are also outcomes attributable to household air pollution through a mediation analysis. With the exception of cataract, all causes share risk curves and are calculated jointly with ambient particulate matter pollution.

Appendix Table 14: MR-BRT relative risk meta-analysis for household air pollution and cataract

Covariate	Gamma	Beta coefficient, logit (95% UI)	Beta coefficient, adjusted (95% UI)
Intercept	0.109	0.939 (0.623–1.278)	2.56 (1.86–3.59)

Appendix Figure 9: Household air pollution and cataract risk literature funnel plot

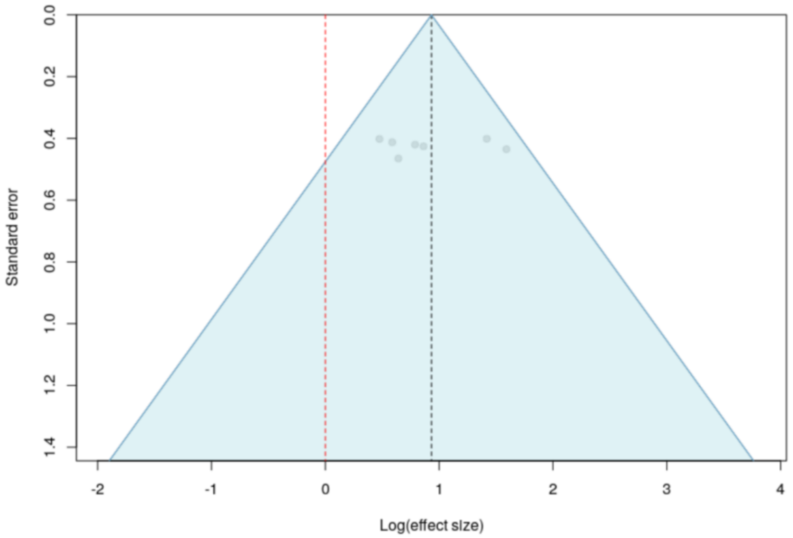

Studies reported effect sizes for males, females, and/or both sexes. In a sensitivity analysis conducted in GBD 2019 and repeated in GBD 2021, we included a covariate for sex and found no significant difference in effect size by sex. We therefore estimate cataract as an outcome of household air pollution in both males and females.

For GBD 2021, we also implemented evidence scoring to provide an empirical measure of strength of evidence for risk-outcome pairs across risk factors in the GBD study (described in more detail elsewhere). Prior to generating an evidence score, we conducted an additional post-analysis step to detect and flag publication bias in the input data. This approach is based on the classic Egger's Regression strategy, which is applied to the residuals in our model. In the current implementation, we

do not correct for publication bias, but flag the risk-outcome pairs where the risk for publication bias is significant. Publication bias was not detected for HAP-cataract risk literature. The resulting evidence score for HAP and cataract was -0.009, which corresponds to a star rating of 1.

In GBD 2021, we also made key changes to our particulate matter risk curves. These risk curves, the mediation analysis for birthweight and gestational age, and the joint-estimation PAF approach are described in the Ambient Particulate Matter Pollution appendix.

$PM_{2.5}$ mapping value estimation

To calculate relative risks from particulate matter risk curves for individuals using solid fuels for cooking, we first estimated the $PM_{2.5}$ exposure level resulting from usage of each fuel type. Input data for the HAP mapping model included indoor and personal measurement data from the WHO Global Household Air Pollution Measurements database, which contains 196 studies with measurements from 43 countries of various pollution metrics in households using solid fuel for cooking.[27] For GBD 2021, we also added data from the PURE-AIR study published in 2020, which includes additional measurements from 120 rural locations in Bangladesh, Chile, China, Colombia, India, Pakistan, Tanzania, and Zimbabwe.[28] The final dataset included 390 estimates from 76 studies in 47 unique locations. We included 281, 81, 9, and 19 measurements for indoor exposure and personal monitors for females, children (under 5), and males, respectively. 314 estimates were in households using solid fuels, 61 in households using clean fuels (gas or electricity) only, and 15 in households using a mixture of solid and clean fuels. Of measurements from households using solid fuels, we included 40, 20, 13, 155, and 86 measurements for coal, crop, dung, wood, and mixed fuels, respectively.

The following models were used to predict log-transformed estimates of excess $PM_{2.5}$ for each individual fuel type (coal, crop, dung, wood) and for the parent solid category. Predictions for the parent solid category were used only to prepare relative risk input data for analysis, not for predicting individual exposure to $PM_{2.5}$ from solid fuel use.

Fuel types:

$$\log(excess\ PM) \sim crop + coal + dung + wood + measure\ group + 24\ hr\ measurement + LDI + (1|study)$$

Solid:

$$\log(excess\ PM) \sim solid + measure\ group + 24\ hr\ measurement + LDI + (1|study)$$

Where,

- 24-hour measurement: binary variable equal to 1 if the measurement occurred over at least a 24-hour period and not only during mealtimes
- Measure group: categorical variable indicating indoor, female, male, or children
- Solid: indicator variable equal to 1 if the measurements were among households using solid fuel only, 0.5 if the measurements represented a mix of clean and solid fuels, and 0 if the households only used clean fuels.

For previous GBD cycles, we also included the Socio-demographic Index (SDI) as a variable to predict a unique value of HAP for each location and year based on development. For GBD 2021, we updated the HAP mapping model to predict unique values from the lag-distributed income per capita (LDI).

Evaluations of model fit using root mean square error (RMSE) indicated that LDI is a more suitable predictor of excess $PM_{2.5}$. We also included a random effect on study and weighted each study by the square root of its sample size.

Before modelling, we subtracted off the GBD 2019 prediction of ambient $PM_{2.5}$ in the study location and year to calculate the excess particulate matter for individuals using solid fuel. The final model coefficients are included below:

Appendix Table 15: HAP mapping model and coefficients

**Variable**	**Beta, log (95% UI)**	**Beta, exponentiated (95% UI)**
Intercept	5.34 (5.16–5.52)	208.51 (174.16–249.64)
Fuel type		
• Clean (ref)		
• Crop	3.15 (3.06–3.25)	23.34 (21.33–25.79)
• Coal	1.66 (1.57–1.73)	5.26 (4.81–5.64)
• Dung	2.35 (2.22–2.48)	10.49 (9.21–11.94)
• Wood	1.99 (1.94–2.04)	7.32 (6.96–7.69)
Measure group		
• Indoor (ref)		
• Female	-0.37 (-0.42 to -0.32)	0.69 (0.66–0.73)
• Male	-0.27 (-0.36 to -0.18)	0.76 (0.70–0.84)
• Child	-1.09 (-1.19 to -1.00)	0.34 (0.30–0.37)
24-hour measurement	-0.68 (-0.83 to -0.54)	0.51 (0.44–0.58)
LDI	-0.000293 (-0.000494 to -0.0000837)	1.00 (1.00–1.00)

To derive final predicted $PM_{2.5}$ exposure values due to solid fuel usage, instead of using direct model outputs for males and children, we scaled $PM_{2.5}$ exposure values for females to the other two groups. There are few studies of personal monitoring in men and children, so we derived ratios of female-male and female-child exposures using studies that reported PM exposure values for females and one or both of the other groups. To calculate these ratios, we first subtracted off the outdoor value from each PM measurement (using GBD 2019 ambient $PM_{2.5}$ predictions as above for $PM_{2.5}$ studies and the studies' published values for PM_4 and PM_{10} studies) and then calculated ratios weighted by sample size.

Appendix Table 16: HAP mapping personal monitoring input observations

**Study**	**Location**	**Year**	**Pollutant**	**Female N**	**Female PM**	**Group**	**N**	**PM**	**Outdoor**

Balakrishnan et al, 2004	Andhra Pradesh, Rural	2004	PM4	591	352	male	503	187	94
Gao X et al, 2009.	Tibet	2009	PM2.5	52	127	male	85	111	78
Dasgupta et al, 2006	Bangladesh	2006	PM10	944	209	male	944	166	50
Devkumar et al, 2014	Nepal	2014	PM2.5	405	169	male	429	167	167
Balakrishnan et al, 2004	Andhra Pradesh, Rural	2004	PM4	591	352	child	56	262	94
Dionisio et al, 2008.	Republic of the Gambia	2008	PM2.5	13	275	child	13	219	147
Dasgupta et al, 2006	Bangladesh	2006	PM10	944	209	child	944	199	50
Gurley et al, 2013	Bangladesh	2013	PM2.5			child	37	308	
Shupler et al, 2020	Sub-Saharan Africa	2018	PM2.5	37	153	male	20	120	26.05
Shupler et al, 2020	India	2018	PM2.5	11	150	male	5	178	42.3
Shupler et al, 2020	India	2018	PM2.5	63	89	male	48	82	42.3
Shupler et al, 2020	South Asia	2018	PM2.5	5	148	male	3	147	64
Shupler et al, 2020	South Asia	2018	PM2.5	27	148	male	17	90	64
Shupler et al, 2020	South Asia	2018	PM2.5	5	147	male	2	73	64
Shupler et al, 2020	South Asia	2018	PM2.5	15	183	male	6	135	64
Shupler et al, 2020	Latin America and Caribbean	2018	PM2.5	24	39	male	12	40	27.2
Shupler et al, 2020	China	2018	PM2.5	36	71	male	35	61	58.9
Shupler et al, 2020	China	2018	PM2.5	23	94	male	21	93	58.9
Shupler et al, 2020	China	2018	PM2.5	55	45	male	47	44	58.9
Shupler et al, 2020	China	2018	PM2.5	4	64	male	3	37	58.9

The final ratios, updated with information from the 2020 PURE-AIR study, were 0.85 (95% UI 0.67–1.09) for children and 0.64 (0.52–0.79) for males compared to 0.85 (0.56–1.31) for children and 0.64 (0.45–0.91) for males in GBD 2019. These results were used to scale the $PM_{2.5}$ mapping model fuel-type-specific predictions for these age and sex groups to calculate relative risks from the $PM_{2.5}$ risk curves.

HAP population-attributable fractions (PAFs) are calculated jointly with those for ambient particulate matter pollution. Details of PAF calculation, relative risks, and evidence scores for all outcomes besides cataract are provided in the Ambient Particulate Matter Pollution appendix.

Secondhand Smoke

Appendix Figure 10: Flowchart of secondhand smoke estimation

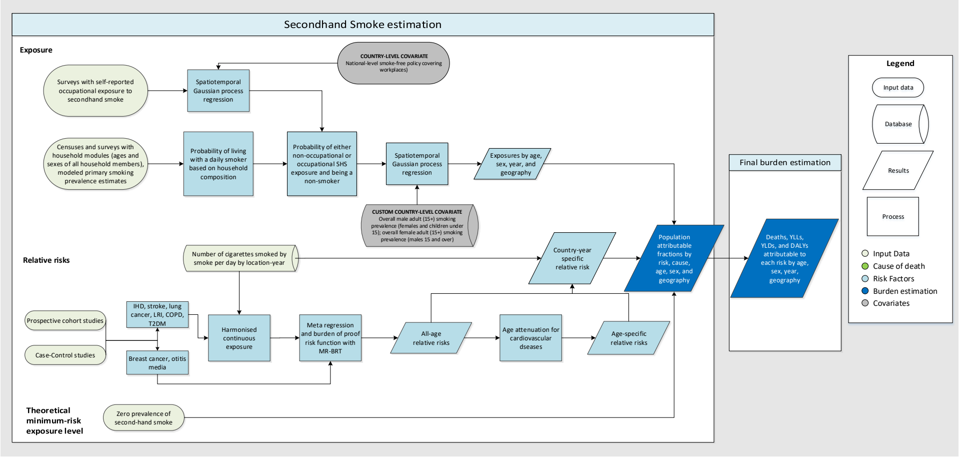

Exposure

Case definition

We define secondhand smoke exposure as current exposure to secondhand tobacco smoke at home or at work. We use household composition as a proxy for household secondhand smoke exposure and make the assumption that all persons living with a daily smoker are exposed to tobacco smoke. We use surveys to estimate the proportion of the population exposed to secondhand smoke at work. We only consider non-smokers to be exposed to secondhand smoke. Non-smokers are defined as all persons who are not daily smokers. Ex-smokers and occasional smokers are considered non-smokers in this analysis. Exposure is evaluated for both children and adults.

Input data

To calculate the proportion of non-smokers who live with at least one daily smoker, two types of data were used: 1) unit record data on household composition, which included the ages and sexes of all persons living in the same household, and 2) GBD daily smoking estimates for each location, year, sex, and age group. Major survey series with a household composition module – including the Demographic Health Surveys (DHS), the Multiple Indicator Cluster Surveys (MICS), and the Living Standards Measurement Surveys (LSMS) – and national and subnational censuses, which included those captured in the Integrated Public Use Microdata Series (IPUMS) project, were used.

To calculate the proportion of the population exposed to secondhand smoke at work, by age and sex, we used cross-sectional surveys that ask respondents about self-reported occupational secondhand smoke exposure. Sources include the Global Adult Tobacco Surveys (GATS), Eurobarometer Surveys, WHO Stepwise Approach to NCD Risk Factor Surveillance (STEPS) Surveys, and other regional and national survey series.

We updated our systematic review in GBD 2021 by searching the Global Health Data Exchange (GHDx) using the keywords “environmental tobacco smoke”, for workplace exposure, and “family composition”,

for identifying household composition modules. We prioritised extraction of surveys used for estimating exposure at the workplace and of new household modules for filing in location and time gaps. Sources that reported exposure to secondhand smoke in a setting other than the workplace were not used. Due to the type of analysis performed, we restricted our data sources to those with available microdata (tabulated data-only sources were excluded). Given the nature of the data used in our models (microdata), no crosswalk for case definition adjustment or age and sex splitting processes were required. Table 1 provides a summary of the exposure input data.

Appendix Table 17: Data inputs for exposure for secondhand smoke

	**Countries with data**	**New sources**	**Total sources**
Exposure	176	480	1198

Modelling strategy

Identical to GBD 2019, we estimated the probability that each person is living with a smoker and is also a non-smoker themselves using set theory. Household composition data were used at the individual level to capture the ages and sexes of each person in the household. In the past, we analyzed surveys with both household composition data and tobacco use questions and determined that the distribution of household size, mean age of the household members, and the age distribution were not significantly different between households with and without a self-reported smoker. Since we did not find that household composition varied between smokers and non-smokers, we then used the updated GBD 2021 daily smoking prevalence estimates to calculate the probability that each household member is a daily smoker. Next, we used the probability of the union of sets on each individual household member to calculate the overall probability that at least one of the other household members was a daily smoker.

As in GBD 2019, we incorporated occupational exposure by modelling prevalence of current exposure to secondhand smoke at work, by age, sex, location, and year, in a three-step spatiotemporal Gaussian process regression (ST-GPR), which generates exposure estimates from a mixed-effects hierarchical linear model plus weighted residuals smoothed across time, space, and age. For this, we first processed all data to capture exposure to secondhand smoke at work among anyone working primarily indoors. Using information from survey-specific gateway questions, we considered all those not currently working or not currently working primarily indoors not exposed to secondhand smoke.

The processed microdata was used to generate a complete time series from 1990 to 2022 for the proportion of the population exposed to tobacco smoke at the workplace using the ST-GPR. The first step of the ST-GPR process is a linear mixed-effects regression of our data on a set of potentially predictive covariates. In addition to the daily smoking prevalence estimates taken from the GBD study covariates database, in GBD 2021 we incorporated a dummy covariate to reflect if a national-level smoking ban covering workplaces was in place in each location-year. The data used to create this covariate came mainly from several iterations of the WHO report on the global tobacco epidemic.

With the estimated workplace exposure from ST-GPR, in order to avoid double counting, we calculated the probability that an individual is exposed through either household exposure or occupational exposure, given their age, sex, and household composition. Lastly, we multiplied this probability of exposure by the probability that the individual is not a smoker themselves (ie, 1 minus primary daily

smoking prevalence for that person's location, year, age, and sex). We then collapsed these individual-level probabilities to produce average probabilities of exposure by location, year, age, and sex.

These final probabilities were modelled in the GBD ST-GPR framework. The linear model formula was fit separately by sex using restricted maximum likelihood in R. We used the sex-specific overall daily smoking prevalence for adults (age 15 and older) as a country-level covariate in the model. The overall male adult daily smoking prevalence was used as the covariate for females of all ages and for males under age 15. The overall female adult daily smoking prevalence was used as the covariate for males age 15 and older.

All input datapoints from the probability calculation had a measure of uncertainty (variance and sample size) coming from the uncertainty of the primary smoking prevalence model and the sample size from the unit record data going into the modelling process. Geographical r*andom effects were used in model fitting but were not used in prediction.*

Theoretical minimum risk exposure level

The theoretical minimum risk exposure level for secondhand smoke is zero exposure among non-smokers, meaning that non-smokers would not live with any daily smokers and would not be exposed to tobacco smoke at their workplace.

Relative risks

The same risk-outcome pairs from GBD 2019 were used. For children ages 0–14, we estimated the burden of otitis media attributable to secondhand smoke exposure. For all ages, we estimated the burden of lower respiratory infections (LRI) and for adults greater than or equal to 25 years of age, we estimated the burden of lung cancer, chronic obstructive pulmonary disease (COPD), ischaemic heart disease (IHD), ischaemic stroke, breast cancer, and type 2 diabetes (T2DM).

Input data

In GBD 2021, we moved from deriving our relative risks from the integrated exposure response curves (IER) for $PM_{2.5}$ air pollution to creating relative risk curves using secondhand smoke-specific studies. We conducted an updated systematic review for studies published before December 31, 2019, evaluating the relationship between exposure to secondhand smoke and risk of IHD, stroke, COPD, breast cancer, and otitis media. We searched for studies in PubMed using the search strings reported in Table 2. Meta-analysis identified through our search were reviewed and underlying studies were considered for inclusion if not previously captured by our search strings. For the remaining outcomes – lung cancer, LRI, and T2DM –, we selected the secondhand smoke studies from the database that was used in GBD 2019 for generating the IER curve.

Appendix Table 18: Search strings used to search PubMed database

Outcome	String
Ischaemic heart disease	(Tobacco smoke pollution [MeSH Terms] OR second-hand[Title/Abstract] OR secondhand[Title/Abstract] OR environmental tobacco[Title/Abstract] OR tobacco smoke[Title/Abstract] OR cigarette smoke[Title/Abstract] OR passive smok*[Title/Abstract] OR involuntary smok*[Title/Abstract] OR parental smoking[Title/Abstract] OR maternal smoking[Title/Abstract]) AND (Coronary Artery Disease[MeSH] OR Myocardial Ischemia[MeSH] OR atherosclerosis[MeSH] OR Coronary Artery

	Disease[Title/Abstract] OR Myocardial Ischemia[Title/Abstract] OR cardiac ischemia[Title/Abstract] OR silent ischemia[Title/Abstract] OR atherosclerosis [Title/Abstract] OR Ischaemic heart disease[Title/Abstract] OR Ischemic heart disease[Title/Abstract] OR coronary heart disease[Title/Abstract] OR myocardial infarction[Title/Abstract] OR heart attack[Title/Abstract] OR heart infarction[Title/Abstract]) AND (Case-Control Studies[MeSH Terms] OR Cross-Over Studies[MeSH Terms] OR Cohort Studies[MeSH Terms] OR Systematic Review[Publication Type] OR Meta-Analysis[Publication Type] OR "systematic review"[Title/Abstract] OR "meta-analysis"[Title/Abstract] OR "cohort"[Title/Abstract] OR "cross-over"[Title/Abstract] OR "crossover"[Title/Abstract] OR "case-control"[Title/Abstract] OR "prospective"[Title/Abstract] OR "retrospective"[Title/Abstract] OR "longitudinal"[Title/Abstract] OR "follow-up"[Title/Abstract] OR Dose-Response Relationship, Drug[MeSH Terms] OR "dose-response"[Title/Abstract]) AND (Risk[MeSH Terms] OR Odds Ratio[MeSH Terms] OR "risk"[Title/Abstract] OR "odds ratio"[Title/Abstract] OR "cross-product ratio"[Title/Abstract] OR "hazards ratio"[Title/Abstract] OR "hazard ratio"[Title/Abstract]) AND ("1970/01/01"[PDat] : "2019/12/31"[PDat]) AND (English[LA]) NOT (animals[MeSH Terms] NOT Humans[MeSH Terms])
Ischaemic stroke	(Tobacco smoke pollution [MeSH Terms] OR second-hand[Title/Abstract] OR secondhand[Title/Abstract] OR environmental tobacco[Title/Abstract] OR tobacco smoke[Title/Abstract] OR cigarette smoke[Title/Abstract] OR passive smok*[Title/Abstract] OR involuntary smok*[Title/Abstract] OR parental smoking[Title/Abstract] OR maternal smoking[Title/Abstract]) AND (brain infarction[MeSH Terms] OR stroke[MeSH Terms] OR intracranial hemorrhages[MeSH Terms] OR "stroke"[Title/Abstract] OR "brain infarction"[Title/Abstract] OR "cerebral infarction"[Title/Abstract] OR "intracerebral hemorrhage"[Title/Abstract] OR "intracerebral haemorrhage"[Title/Abstract] OR "subarachnoid hemorrhage"[Title/Abstract] OR "subarachnoid haemorrhage"[Title/Abstract]) AND (Case-Control Studies[MeSH Terms] OR Cross-Over Studies[MeSH Terms] OR Cohort Studies[MeSH Terms] OR Systematic Review[Publication Type] OR Meta-Analysis[Publication Type] OR "systematic review"[Title/Abstract] OR "meta-analysis"[Title/Abstract] OR "cohort"[Title/Abstract] OR "cross-over"[Title/Abstract] OR "crossover"[Title/Abstract] OR "case-control"[Title/Abstract] OR "prospective"[Title/Abstract] OR "retrospective"[Title/Abstract] OR "longitudinal"[Title/Abstract] OR "follow-up"[Title/Abstract] OR Dose-Response Relationship, Drug[MeSH Terms] OR "dose-response"[Title/Abstract]) AND (Risk[MeSH Terms] OR

	Odds Ratio[MeSH Terms] OR “risk”[Title/Abstract] OR “odds ratio”[Title/Abstract] OR “cross-product ratio”[Title/Abstract] OR “hazards ratio”[Title/Abstract] OR “hazard ratio”[Title/Abstract]) AND ("1970/01/01"[PDat] : "2019/12/31"[PDat]) AND (English[LA]) NOT (animals[MeSH Terms] NOT Humans[MeSH Terms])
Chronic obstructive pulmonary disease	(Tobacco smoke pollution [MeSH Terms] OR second-hand[Title/Abstract] OR secondhand[Title/Abstract] OR environmental tobacco[Title/Abstract] OR tobacco smoke[Title/Abstract] OR cigarette smoke[Title/Abstract] OR passive smok*[Title/Abstract] OR involuntary smok*[Title/Abstract] OR parental smoking[Title/Abstract] OR maternal smoking[Title/Abstract]) AND (Pulmonary Disease, Chronic Obstructive[MeSH] OR "COPD"[Title/Abstract] OR "emphysema"[Title/Abstract] OR "chronic obstructive pulmonary disease"[Title/Abstract]) AND (Case-Control Studies[MeSH Terms] OR Cross-Over Studies[MeSH Terms] OR Cohort Studies[MeSH Terms] OR Systematic Review[Publication Type] OR Meta-Analysis[Publication Type] OR “systematic review”[Title/Abstract] OR “meta-analysis”[Title/Abstract] OR “cohort”[Title/Abstract] OR “cross-over”[Title/Abstract] OR “crossover”[Title/Abstract] OR “case-control”[Title/Abstract] OR “prospective”[Title/Abstract] OR “retrospective”[Title/Abstract] OR “longitudinal”[Title/Abstract] OR “follow-up”[Title/Abstract] OR Dose-Response Relationship, Drug[MeSH Terms] OR “dose-response”[Title/Abstract]) AND (Risk[MeSH Terms] OR Odds Ratio[MeSH Terms] OR “risk”[Title/Abstract] OR “odds ratio”[Title/Abstract] OR “cross-product ratio”[Title/Abstract] OR “hazards ratio”[Title/Abstract] OR “hazard ratio”[Title/Abstract]) AND ("1970/01/01"[PDat] : "2019/12/31"[PDat]) AND (English[LA]) NOT (animals[MeSH Terms] NOT Humans[MeSH Terms])
Breast cancer	(Tobacco smoke pollution [MeSH Terms] OR second-hand[Title/Abstract] OR secondhand[Title/Abstract] OR environmental tobacco[Title/Abstract] OR tobacco smoke[Title/Abstract] OR cigarette smoke[Title/Abstract] OR passive smok*[Title/Abstract] OR involuntary smok*[Title/Abstract] OR parental smoking[Title/Abstract] OR maternal smoking[Title/Abstract]) AND (breast neoplasm[MeSH Terms] OR “breast cancer”[Title/Abstract] OR “breast cancers”[Title/Abstract] OR “breast neoplasm”[Title/Abstract] OR “breast neoplasms”[Title/Abstract] OR “mammary cancer”[MeSH Terms] OR “mammary cancers”[Title/Abstract] OR “breast malignant neoplasm”[Title/Abstract] OR “breast malignant neoplasms”[Title/Abstract] OR “mammary carcinoma”[Title/Abstract] OR “mammary carcinomas”[Title/Abstract] OR “breast carcinoma”[Title/Abstract] OR “breast carcinomas”[Title/Abstract] OR “mammary neoplasm”[Title/Abstract] OR “mammary

	neoplasms"[Title/Abstract] OR "breast tumor"[Title/Abstract] OR "breast tumors"[Title/Abstract] OR "cancer of the breast"[Title/Abstract] OR "cancers of the breast"[Title/Abstract] OR "neoplasm of the breast"[Title/Abstract] OR "tumor of the breast"[Title/Abstract]) AND (Case-Control Studies[MeSH Terms] OR Cross-Over Studies[MeSH Terms] OR Cohort Studies[MeSH Terms] OR Systematic Review[Publication Type] OR Meta-Analysis[Publication Type] OR "systematic review"[Title/Abstract] OR "meta-analysis"[Title/Abstract] OR "cohort"[Title/Abstract] OR "cross-over"[Title/Abstract] OR "crossover"[Title/Abstract] OR "case-control"[Title/Abstract] OR "prospective"[Title/Abstract] OR "retrospective"[Title/Abstract] OR "longitudinal"[Title/Abstract] OR "follow-up"[Title/Abstract] OR Dose-Response Relationship, Drug[MeSH Terms] OR "dose-response"[Title/Abstract]) AND (Risk[MeSH Terms] OR Odds Ratio[MeSH Terms] OR "risk"[Title/Abstract] OR "odds ratio"[Title/Abstract] OR "cross-product ratio"[Title/Abstract] OR "hazards ratio"[Title/Abstract] OR "hazard ratio"[Title/Abstract]) AND ("1970/01/01"[PDat] : "2019/12/31"[PDat]) AND (English[LA]) NOT (animals[MeSH Terms] NOT Humans[MeSH Terms])
Otitis media	(Tobacco smoke pollution [MeSH Terms] OR second-hand[Title/Abstract] OR secondhand[Title/Abstract] OR environmental tobacco[Title/Abstract] OR tobacco smoke[Title/Abstract] OR cigarette smoke[Title/Abstract] OR passive smok*[Title/Abstract] OR involuntary smok*[Title/Abstract] OR parental smoking[Title/Abstract] OR maternal smoking[Title/Abstract]) AND (Otitis Media[MeSH Terms] OR "otitis media"[Title/Abstract] OR "middle ear infection" [Title/Abstract] OR "middle ear disease" [Title/Abstract] OR "ear infection"[Title/Abstract] OR "ear disease"[Title/Abstract] OR "otitis" [Title/Abstract]) AND (Case-Control Studies[MeSH Terms] OR Cross-Over Studies[MeSH Terms] OR Cohort Studies[MeSH Terms] OR Systematic Review[Publication Type] OR Meta-Analysis[Publication Type] OR "systematic review"[Title/Abstract] OR "meta-analysis"[Title/Abstract] OR "cohort"[Title/Abstract] OR "cross-over"[Title/Abstract] OR "crossover"[Title/Abstract] OR "case-control"[Title/Abstract] OR "prospective"[Title/Abstract] OR "retrospective"[Title/Abstract] OR "longitudinal"[Title/Abstract] OR "follow-up"[Title/Abstract] OR Dose-Response Relationship, Drug[MeSH Terms] OR "dose-response"[Title/Abstract]) AND (Risk[MeSH Terms] OR Odds Ratio[MeSH Terms] OR "risk"[Title/Abstract] OR "odds ratio"[Title/Abstract] OR "cross-product ratio"[Title/Abstract] OR "hazards ratio"[Title/Abstract] OR "hazard ratio"[Title/Abstract]) AND ("1970/01/01"[PDat] : "2019/12/31"[PDat]) AND (English[LA]) NOT (animals[MeSH Terms] NOT Humans[MeSH Terms])

Appendix Figure 11: PRISMA 2020 flow diagram for a new systematic review of the secondhand smoke and ischemic heart disease risk-outcome pair in GBD 2021

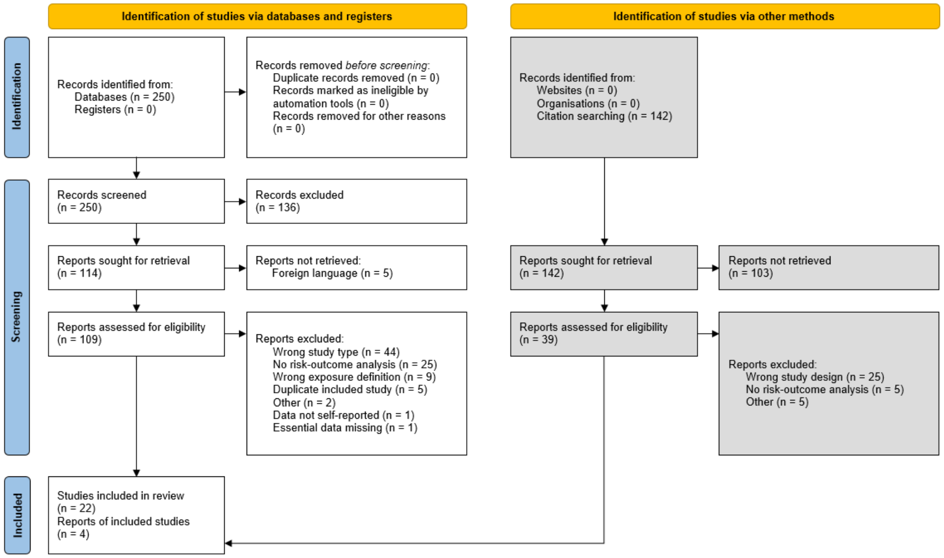

From: Page MJ, McKenzie JE, Bossuyt PM, Boutron I, Hoffmann TC, Mulrow CD, et al. The PRISMA 2020 statement: an updated guideline for reporting systematic reviews. BMJ 2021;372:n71. doi: 10.1136/bmj.n71. For more information, visit: http://www.prisma-statement.org/

Appendix Figure 12: PRISMA 2020 flow diagram for a new systematic review of the secondhand smoke and ischaemic stroke risk-outcome pair in GBD 2021

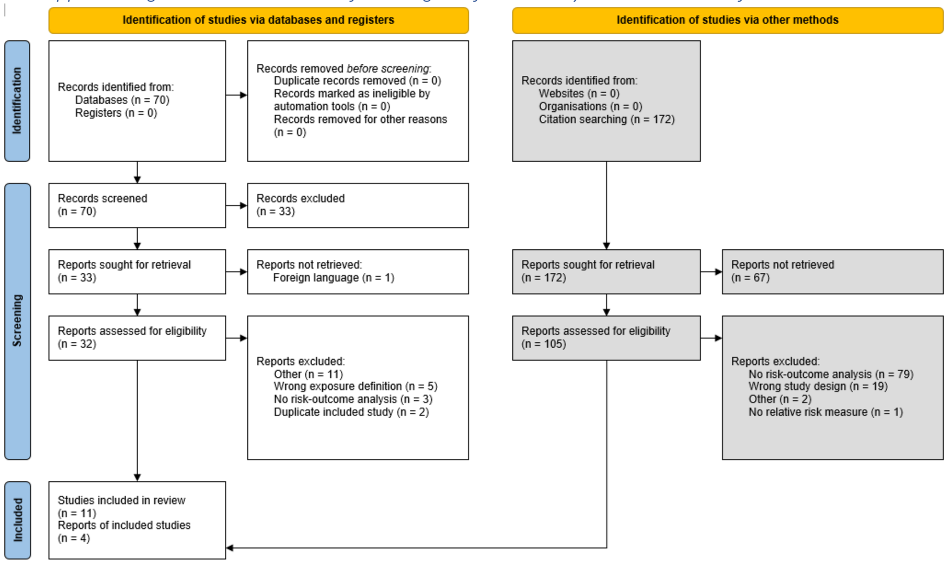

From: Page MJ, McKenzie JE, Bossuyt PM, Boutron I, Hoffmann TC, Mulrow CD, et al. The PRISMA 2020 statement: an updated guideline for reporting systematic reviews. BMJ 2021;372:n71. doi: 10.1136/bmj.n71. For more information, visit: http://www.prisma-statement.org/

Appendix Figure 13: PRISMA 2020 flow diagram for a new systematic review of the secondhand smoke and chronic obstructive pulmonary disease risk-outcome pair in GBD 2021

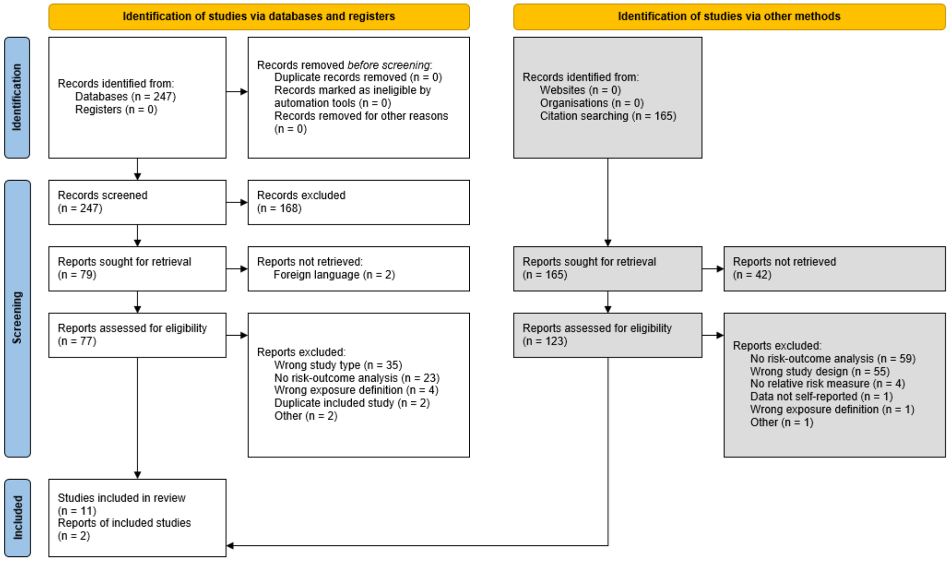

From: Page MJ, McKenzie JE, Bossuyt PM, Boutron I, Hoffmann TC, Mulrow CD, et al. The PRISMA 2020 statement: an updated guideline for reporting systematic reviews. BMJ 2021;372:n71. doi: 10.1136/bmj.n71. For more information, visit: http://www.prisma-statement.org/

Appendix Figure 14: PRISMA 2020 flow diagram for a new systematic review of the secondhand smoke and breast cancer risk-outcome pair in GBD 2021

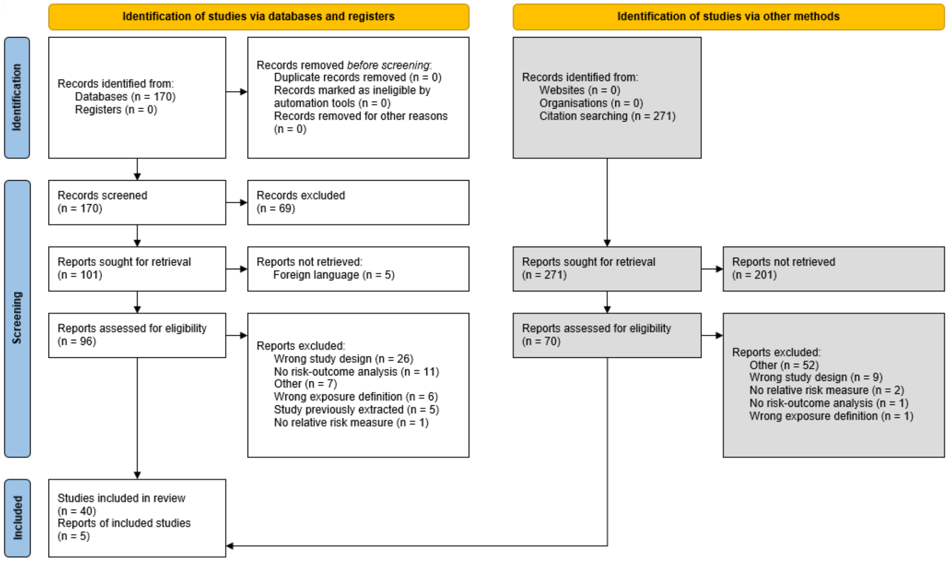

From: Page MJ, McKenzie JE, Bossuyt PM, Boutron I, Hoffmann TC, Mulrow CD, et al. The PRISMA 2020 statement: an updated guideline for reporting systematic reviews. BMJ 2021;372:n71. doi: 10.1136/bmj.n71. For more information, visit: http://www.prisma-statement.org/

Appendix Figure 15: PRISMA 2020 flow diagram for a new systematic review of the secondhand smoke and otitis media risk-outcome pair in GBD 2021

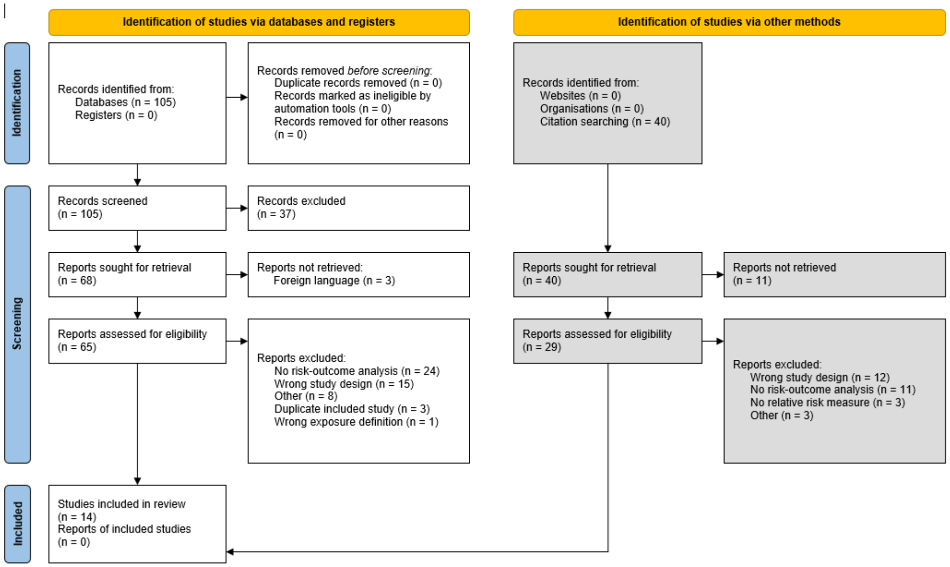

From: Page MJ, McKenzie JE, Bossuyt PM, Boutron I, Hoffmann TC, Mulrow CD, et al. The PRISMA 2020 statement: an updated guideline for reporting systematic reviews. BMJ 2021;372:n71. doi: 10.1136/bmj.n71. For more information, visit: http://www.prisma-statement.org/

We included prospective cohort studies and case-control studies that assessed exposure to secondhand smoke as a binary, categorical (level of exposure: low, moderate, high), or continuous (cigarettes per day) exposure, excluding studies that reported exposure using a different continuous metric (eg, number of hours, number of people, number of days, level of cotinine, etc.) or score. Further, we only included studies that reported risk estimates (relative risk, hazard ratio, or odds ratio) with confidence intervals, standard errors, or enough information to quantify uncertainty. In addition, we excluded studies that only reported former exposure to secondhand smoke (eg, child exposure during pregnancy) or only exposure among current smokers. Table 3 summarises the relative risk input data used in GBD 2021.

Appendix Table 19: Data inputs for relative risk for secondhand smoke

**Input data**	**Relative risk**
Source count (total)	124
Number of countries with data	33

In future rounds of the GBD, we aim to conduct systematic reviews for the outcomes not updated this round and incorporate new evidence for all outcomes as they become available. In addition, we will evaluate the evidence concerning the relationship between exposure to secondhand smoke and other diseases and add these risk-outcome pairs if general GBD inclusion criteria are met.

Modelling strategy

Prior to GBD 2021, lung cancer, IHD, stroke, and COPD risk curves were calculated jointly with ambient particulate matter pollution, while relative risks for otitis media, breast cancer, and diabetes were derived from published meta-analyses. In GBD 2021, we used the meta-regression—Bayesian, regularised, trimmed (MR-BRT) tool to estimate the log relative risk associated with each level of secondhand smoke exposure on a continuous scale for lung cancer, IHD, stroke, COPD, LRI, and T2DM. For this, we converted binary and categorical exposures reported in each study to a common continuous metric representing the number of cigarettes smoked per smoker per day in each location-year (Table 4). If a study reported exposure in number of cigarettes, we used that number directly.

Appendix Table 20: Converting exposure to a continuous scale

**Study reported exposure**	**Matched continuous exposure**
Binary	Median of the distribution of cigarettes smoked per smoker per day in the study location-year
Categorical	**Low:** 25th percentile of the distribution of cigarettes smoked per smoker per day in a specific location-year<br>**Medium:** Median of the distribution of cigarettes smoked per smoker per day in a specific location-year<br>**High:** 75th percentile of the distribution of cigarettes smoked per smoker per day in a specific location-year
Continuous (cigarettes per day)	Direct number reported associated with the relative risk reported in the study

For breast cancer and otitis media, we used the MR-BRT tool to perform our own meta-regression analysis of the risk of developing these conditions for those currently exposed to tobacco smoke relative to the reference category of those not exposed. For these outcomes, only studies reporting a binary exposure were included in the analysis. Table 5 shows the results of the MR-BRT analyses for the outcomes with dichotomous exposure.

Appendix Table 21: Otitis media and breast cancer MR-BRT network meta-analysis results (reference: not exposed to secondhand smoke)

**Outcome**	**GBD 2019 relative risk**	**GBD 2021 MR-BRT relative risk**
Otitis media	1.37 (1.25–1.50)	1.23 (1.051.45)
Breast cancer	1.07 (1.02–1.13)	1.04 (0.951.13)

For each risk-outcome pair meta-regression, we considered study-level covariates that could potentially bias the study's reported effect size estimates. These study-level covariates included indication of the study design, whether the study used a washout period, whether the study determined outcomes based on administrative records or self-reports, whether the study was generalisable to the general population, and the level of adjustment for relevant confounders like age, sex, smoking, education, and income. We also created covariates to indicate aspects related to the secondhand smoke exposure reported in each study, such as source of exposure (ie, spouse, maternal), exposure setting (ie, work, home, any), exposed population (ie, never smoker, non-smokers), and others. We adjusted for these covariates in our meta-regression if they significantly biased our estimated relative risk function. We used the MR-BRT automated covariate selection process to identify the statistically significant covariates (significance threshold = 0.05). For outcomes with enough datapoints, we introduce likelihood-based trimming to detect and remove outliers (10% trimming) before fitting the model. Outcome-specific model characteristics are described in Table 6.

Appendix Table 22: Risk-outcome pair model specifications and results.

**Outcome**	**MR-BRT models specifications**	**Trimming**	**Selected covariates**	**Mean gamma solution**	**Publication bias**
Continuous (risk curves)					
Ischaemic heart disease	Quadratic splines with 3 internal knots; right linear tail; monotonically increasing constraint; Gaussian prior (0, 0.01) on max derivative of non-linear intervals	Yes	Cohort study	0.028	No
Ischaemic stroke	Cubic splines with 3 internal knots; right linear tail; monotonically increasing constraint; Gaussian prior (0, 0.01) on max derivative of non-linear intervals	Yes	-	0.000	No
Chronic obstructive pulmonary disease	Cubic splines with 3 internal knots; right linear tail; monotonically increasing constraint; Gaussian prior (0, 0.01) on max derivative of non-linear intervals	Yes	-	0.082	No
Lung cancer	Cubic splines with 3 internal knots; right linear tail; monotonically increasing	Yes	-	0.000	No

	constraint; Gaussian prior (0, 0.01) on max derivative of non-linear intervals				
Lower respiratory infection	Cubic splines with 3 internal knots; right linear tail; monotonically increasing constraint; Gaussian prior (0, 0.01) on max derivative of non-linear intervals	Yes	Adjusted model; Multiple exposure measurements; >95% follow-up	2.377	Yes
Type 2 diabetes	Cubic splines with 3 internal knots; right linear tail; monotonically increasing constraint; Gaussian prior (0, 0.01) on max derivative of non-linear intervals	No	-	0.126	No
Dichotomous					
Breast cancer	NA	Yes	Non-smoker population	0.006	No
Otitis media	NA	Yes	Adjusted model	0.014	No

We implemented the Fisher Scoring correction to the heterogeneity parameter, which corrects for data-sparse situations. In such cases, the between-study heterogeneity parameter estimate may be 0, simply from lack of data. The Fisher Scoring correction uses a quantile of gamma, which is sensitive to the number of studies, study design, and reported uncertainty.

Prior to generating an evidence score, we conducted an additional post-analysis step to test and adjust for publication bias in the input data. This approach is based on the classic Egger's Regression strategy, which is applied to the residuals in our model. In the current implementation, we do not correct for publication bias, but flag the risk-outcome pairs where the risk for publication bias is significant. We found evidence of publication bias for LRI studies only.

There is a well-documented attenuation of the risk for cardiovascular disease throughout one's life. Thus, in GBD 2021, to incorporate this age trend in the relative risks, we first identified the median age-at-event across all IHD and stroke cohorts and considered that as the reference age group. We then assigned our risk curves to this reference age group. Next, we applied 1000 draws of the age-specific attenuation factors produced for the smoking curves to 1000 draws of our reference age group's risk curve to determine age-specific risk curves that propagated the uncertainty of both the risk function and age pattern.

Population attributable fraction

For outcomes with a risk a curve, we assigned a specific relative risk to each country-year based on the average number of cigarettes smoked per smoker in that location-year. Relative risks for otitis media and breast cancer from MR-BRT were applied to all countries for all years. Except for IHD and stroke, relative risks were applied to all estimated ages. There was no variation in relative risk by sex. We used the standard GBD population attributable fraction equation for dichotomous risks to estimate burden based on exposure, relative risks, and theoretical minimum risk exposure level.

Low birthweight and short gestation

Appendix Figure 16: Flowchart for low birthweight and short gestation estimation

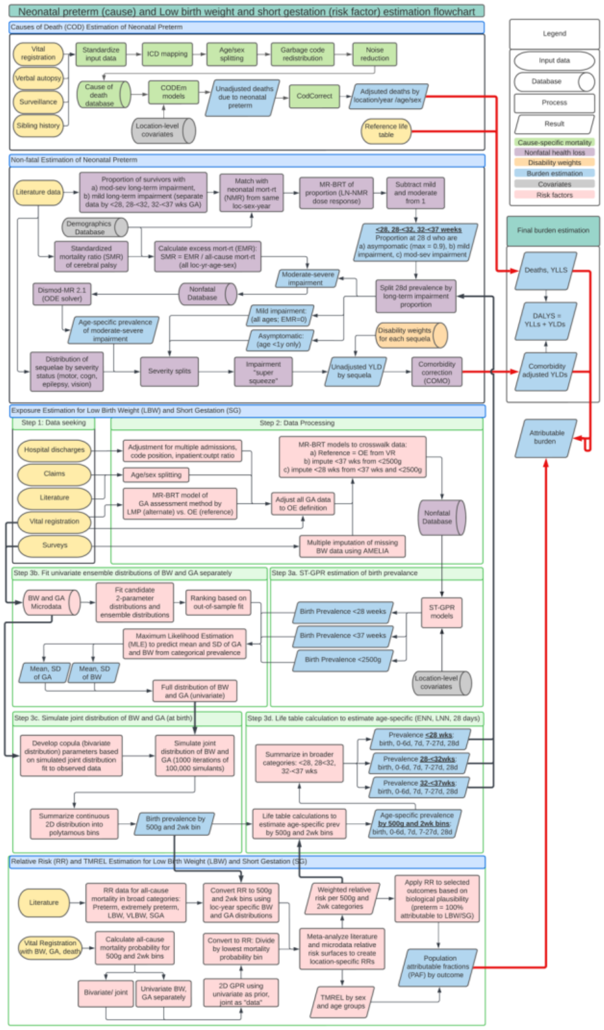

Input data and methodological summary

Short gestational age and low birthweight are highly correlated risk factors associated with poor child health outcomes. The "low birthweight and short gestation" (LBWSG) risk factor quantifies the burden of disease attributable to increased risk of death and disability due to 1) less than ideal birthweight ("low birthweight") and 2) shorter than ideal length of gestation ("short gestation").

Within GBD, attributable burden is generally estimated separately for each individual risk factor, but the combined burden attributable to multiple risk factors is of general interest. In GBD, attributable burden due to multiple risk factors is typically estimated through a "mediation analysis" that is applied after independent estimation of each risk factor's exposure, relative risk, theoretical minimum risk exposure level (TMREL), and population attributable fraction (PAF). In the mediation analysis, a "mediation factor" adjusts the PAF of each risk factor by the amount of attributable burden mediated through the other GBD risk factors. While mediation may be common, direct quantification of the joint exposure, relative risk, and PAF of the combined risk factors is conceptually more straightforward.

In GBD 2016, LBWSG became the first (and, as of GBD 2021, only) group of GBD risk factors in which combined attributable burden is quantified by direct estimation of the joint exposure, relative risk, TMREL, and PAF of multiple risk factors. After first directly estimating the joint exposure, relative risk, TMREL, and PAF of birthweight and gestational age together, we then separate out the independent PAFs due to birthweight only or gestational age only. Because of this modelling strategy, the joint GBD risk factor quantifying the burden of disease due to both less than ideal birthweight ("low birthweight") and shorter than ideal gestational age ("short gestation") is grouped into a single "parent" risk factor termed "low birthweight and short gestation". LBWSG is disaggregated into two "child" risk factors: "low birthweight for gestation" and "short gestation for birthweight". Low birthweight for gestation quantifies the burden of disease attributable to less than ideal birthweight, after adjusting for the influence of gestational age. Likewise, short gestation for birthweight quantifies the burden of disease attributable to shortened gestational age, after adjusting for the influence of birthweight.

Ideally, the model for joint exposure and joint relative risk would be fully continuous. To simplify the computation for the analysis, a grid of 500-gram and 2-week units ("bins") is used as the LBWSG dimensions and to approximate a fully continuous joint distribution model (see Figure 1).

Appendix Figure 17: Fully continuous analysis of joint gestational age and birthweight (left) is approximated with a grid of birthweight and gestational age with 500-gram and 2-week "bins" (right)

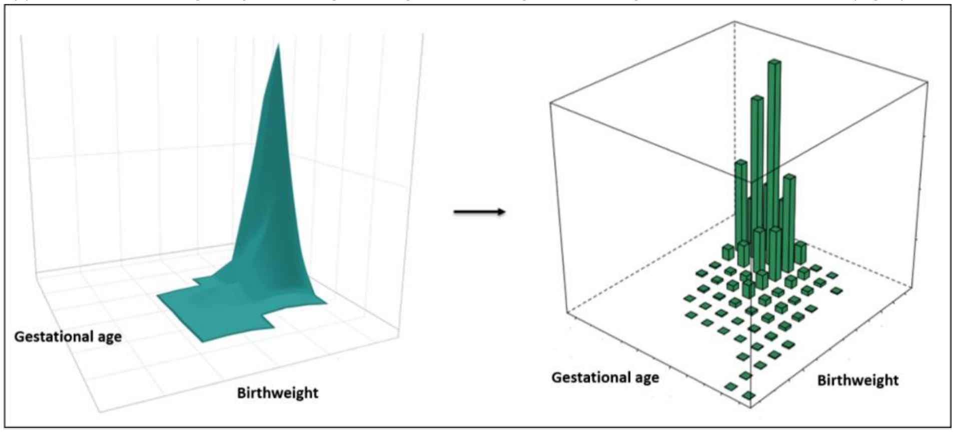

Case definition

"Low birthweight" has historically referred to any birthweight less than 2500 grams, dichotomising birthweight into two categories: "normal" and "low". In the context of the GBD LBWSG risk factor, low birthweight refers to any birthweight less than the birthweight TMREL (the birthweight that minimises risk at the population level). Because LBWSG is estimated in a grid of 500-gram and 2-week bins, any 500-gram birthweight unit less than the TMREL, which was determined as [38, 40) weeks and [3500, 4000) g for the LBWSG parent risk factor, is considered "low birthweight". This includes, for example, birthweight of [2500, 3000) grams, which the traditional, dichotomous definition of "low birthweight" would not include.

Like birthweight, gestational age is typically classified into broad categories. "Preterm" is used to describe any newborn baby born less than 37 completed weeks of gestation. In the GBD context, "short gestation" is used to refer to all gestational ages below the gestational age TMREL.

Exposure

In LBWSG, exposure refers to the portion of the joint distribution of gestational age and birthweight less than the TMREL, by location/year/sex (l/y/s), from birth to the end of the neonatal period. Modelling LBWSG exposure can be summarised in three steps:

A. Model univariate gestational age and birthweight distributions at birth, by l/y/s
B. Model joint distributions of gestational age and birthweight at birth, by l/y/s
C. Model joint distributions from birth to the end of the neonatal period, by l/y/s

Appendix Table 23: Analytic steps in estimation of YLDs due to preterm birth

	Summary of exposure modelling strategy
**Step A**<br><br>Model univariate distributions at birth	1. Model mean gestational age, prevalence of gestational age <28 weeks, and prevalence of gestational age <37 weeks, by l/y/s<br>2. Model mean birthweight and prevalence of birthweight <2500 grams, by l/y/s<br>3. Model univariate gestational age and birthweight distributions separately at birth, by l/y/s
**Step B**<br><br>Model joint distributions at birth	1. Use copulae to model the correlation structure of the joint distribution of gestational age and birthweight, globally<br>2. Model the joint distribution of gestational age and birthweight, by location/year/sex at birth, by applying the globally modelled correlation structure to the location/year/sex-specific univariate models of gestational age and birthweight distributions
**Step C**<br><br>Model joint distributions from birth to 28 days	1. Model all-cause mortality rates by gestational age and birthweight<br>2. Model gestational age and birthweight distributions of surviving neonates for all l/y/s from birth to end of the neonatal period, using all-cause mortality rates by gestational age and birthweight

Input data and data processing

Input data needed to model univariate gestational age and birthweight distributions at birth (Step A):

- Prevalence of preterm birth (<37 weeks), by l/y/s
- Prevalence of preterm birth (<28 weeks), by l/y/s
- Mean gestational age, by l/y/s
- Gestational age microdata
- Prevalence of low birthweight (<2500 grams), by l/y/s
- Mean birthweight, by l/y/s
- Birthweight microdata

To model joint distributions of gestational age and birthweight (Step B), joint microdata of gestational age and birthweight are also required. Additional inputs to modelling joint distributions from birth to 28 days (Step C) are all-cause mortality by l/y/s and joint birthweight and gestational age microdata linked to mortality outcomes.

Prevalence of extremely preterm birth (<28 weeks) and preterm birth (<37 weeks) were modelled using vital registration, survey, and clinical data. For the preterm models, only inpatient and insurance claims data were included from clinical informatics datasets; outpatient data were excluded because they were more likely to capture repeated visits by the same child rather than unique visits. Prevalence of low birthweight (<2500 grams) was modelled using only vital registration and survey data.

Literature review

Before GBD 2016, available preterm birth data were sourced by a technical working group. In GBD 2016 and GBD 2017, we conducted systematic reviews to identify additional sources beyond the data already used in the models. The PubMed database was searched using the following search string:

> ((("Infant, Premature"[Mesh] OR ("infant"[All Fields] AND "premature"[All Fields]) OR "premature infant"[All Fields] OR ("preterm"[All Fields] AND "infant"[All Fields]) OR "preterm infant"[All Fields] OR ("infant, newborn"[MeSH Terms] OR ("infant"[All Fields] AND "newborn"[All Fields]) OR "newborn infant"[All Fields] OR ("newborn"[All Fields] AND "infant"[All Fields])) AND (premature[All Fields] OR preterm[All Fields]) OR "premature birth"[MeSH Terms] OR ("premature"[All Fields] AND "birth"[All Fields]) OR "premature birth"[All Fields] OR ("preterm"[All Fields] AND "birth"[All Fields]) OR "preterm birth"[All Fields]) ((("Infant, Premature"[Mesh] OR ("infant"[All Fields] AND "premature"[All Fields]) OR "premature infant"[All Fields] OR ("preterm"[All Fields] AND "infant"[All Fields]) OR "preterm infant"[All Fields] OR ("infant, newborn"[MeSH Terms] OR ("infant"[All Fields] AND "newborn"[All Fields]) OR "newborn infant"[All Fields] OR ("newborn"[All Fields] AND "infant"[All Fields])) AND (premature[All Fields] OR preterm[All Fields]) OR "premature birth"[MeSH Terms] OR ("premature"[All Fields] AND "birth"[All Fields]) OR "premature birth"[All Fields] OR ("preterm"[All Fields] AND "birth"[All Fields]) OR "preterm birth"[All Fields]) AND ("1985"[PDAT] : "3000"[PDAT]) AND "humans"[MeSH Terms].

The exclusion criteria were: studies that did not provide primary data on epidemiological parameters, non-representative studies (eg, only high-risk pregnancies), and reviews. Table 3 shows the search hits, number of full-texts reviewed, and number of extracted sources.

Appendix Table 24. LBWSG search hits, full-text review, extracted sources

**Search**	**Hits**	**Full-text review**	**Extracted**	**Search date**
GBD 2017	16,174	2200	154	6/6/2017

Appendix Table 25. Input data for exposure models

**Input data**	**Exposure**
Source count (total)	2233
Number of countries with data	176

Data processing

Any data that didn't fit a GBD age groups was split into age groups using a model that was run using only age-specific data. Starting in GBD 2019, as was the case with all other non-fatal analyses, we applied empirical age and sex ratios from previous models to disaggregate observations that did not entirely fit in one GBD age category or sex. Ratios were determined by dividing the result for a specific age and sex by the result for the aggregate age and sex specified in a given observation.

Low birthweight (<2500 grams) data were extracted from literature, vital registration systems, and surveys. Survey data (most commonly from DHS and MICS) were observed to have high missingness of birthweight responses. We evaluated the patterns of missingness and found a number of distinct patterns that suggested non-random omission of birthweight observations. We therefore imputed missing birthweight values using the Amelia II (Version 1.7.6) package in R. Birthweight was predicted using the following variables also in the DHS surveys: urbanicity, sex, birthweight recorded on card, birth

order, maternal education, paternal education, child age, child weight, child height, mother's age at birth, mother's weight, shared toilet facility, and household water treated.

After imputation, we completed a number of additional steps to standardize the dataset by applying a series of crosswalks. "Crosswalking" is a process of reducing non-random bias by adjusting non-standard data to the likely value had the data been collected using a reference definition, technique, or sample. Three crosswalks were applied for birthweight and gestational age data, all of the statistical models for which were developed using meta-regression – regularized, Bayesian, trimmed (MR-BRT).

First was a crosswalk for method of gestational age assessment that included three separate models. All microdata that reported GA and both obstetric estimate (OE) and last menstrual period were crosswalked to OE using the relationship derived from USA GA microdata (Figure 2). This crosswalk was developed with a spline on LMP in order to reliably match on the data that needed to be crosswalked.

Next, for all data that were only categorical, we adjusted all gestational age data to a reference definition of obstetric estimate (OE), which also included tabulations of the crosswalked microdata above. Two alternate definitions regularly appeared and both were crosswalked separately. These were Last Menstrual Period (LMP) for each of <37 weeks and <28 weeks gestation (Tables 5 and 6) and other measure of gestation age (Table 7 and 8).

The second set of crosswalks adjusted data derived from clinical administrative sources (ie. Hospital discharges and insurance claims) to matched vital registration data using OE (Tables 9 and 10).

The third set of crosswalks served to "square the input dataset" to ensure that every location-year with data had an observation for each of <2500g (birthweight), <37 weeks, and <28 weeks. This process utilized relationships between input data types to maximize the volume of data later input to models. Low birthweight data (<2500g) were crosswalked to preterm (<37 weeks) data (Table 11), preterm to extremely preterm (Table 12), and extremely preterm to preterm (Table 13).

Appendix Figure 18. MR-BRT OE-LMP crosswalk adjustment factor by LMP-reported gestational age

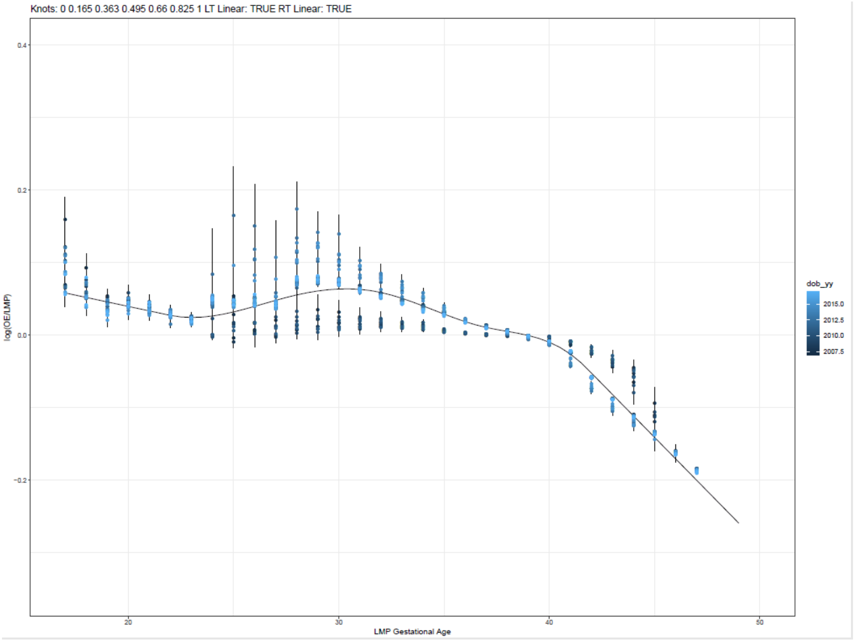

Appendix Figure 18 shows an error-bar plot of OE-LMP crosswalk adjustment factor (log[OE/LMP]) by LMP-based gestational age, using micro-data from the USA. Only individuals with available data for both OE and LMP were included in the analysis. Each plot represents the observed adjustment factor (log[OE/LMP]) by GBD location (state) and birth year of the child, plotted against the observed LMP-based gestational age (on the X axis). In other words, the plots show the point estimates of the adjustment factor by state and birth year, each with an uncertainty interval. The line represents the model-derived overall adjustment factor estimates for all states and years of birth, plotted against the observed LMP-based gestational age (on the X axis).

Appendix Table 26. MR-BRT OE-LMP crosswalk adjustment factor for preterm birth (<37 weeks of gestation)

Data input	Reference or alternative case definition	Gamma	Beta coefficient, log (95% CI)	Adjustment factor (95% UI)*
Obstetric estimate	Reference	0.01	---	---
Last menstrual period	Alternative		0.187 (0.142,0.231)	1.205 (1.153, 1.260)

Appendix Table 27. MR-BRT OE-LMP crosswalk adjustment factor for extremely preterm (<28 weeks gestation)

Data input	Reference or alternative case definition	Gamma	Beta coefficient, log (95% CI)	Adjustment factor (95% UI)*
Obstetric estimate	Reference	0.00	---	---
Last menstrual period	Alternative		0.0284 (0.268,0.300)	1.328 (1.308, 1.349)

Appendix Table 28. MR-BRT OE-other measure crosswalk adjustment factor for preterm birth (<37 weeks gestation)

Data input	Reference or alternative case definition	Gamma	Beta coefficient, log (95% CI)	Adjustment factor (95% UI)*
Obstetric estimate	Reference	0.10	---	---
Other measurement	Alternative		-0.243 (--0.494, 0.009)	0.785 (0.610, 1.01)

Appendix Table 29. MR-BRT OE-other measure crosswalk adjustment factor for extremely preterm birth (<28 weeks gestation)

Data input	Reference or alternative case definition	Gamma	Beta coefficient, log (95% CI)	Adjustment factor (95% UI)*
Obstetric estimate	Reference	0.37	---	---
Other measurement	Alternative		0.154 (-0.486, 0.793)	1.166 (0.615, 2.210)

Appendix *Table 30. MR-BRT VR-claims crosswalk adjustment factor for preterm birth (<37 weeks gestation)*

Data input	Reference or alternative case definition	Gamma	Beta coefficient, log (95% CI)	Adjustment factor (95% UI)*
Vital registration	Reference	0.07	---	---
Insurance claims	Alternative		-0.712 (-0.909, -0.515)	0.491 (0.403, 0.597)

Appendix Table 31. MR-BRT VR-insurance claims crosswalk adjustment factor for extremely preterm birth (<28 weeks of gestation)

Data input	Reference or alternative case definition	Gamma	Beta coefficient, log (95% CI)	Adjustment factor (95% UI)*
Vital registration	Reference	0.02	---	---
Insurance claims	Alternative		-1.258 (-1.447, -1.07)	0.284 (0.235, 0.344)

Appendix Table 32. MR-BRT low birthweight to preterm birth (<37 weeks gestation)

Data input	Reference or alternative case definition	Gamma	Beta coefficient, log (95% CI)	Adjustment factor (95% UI)*
Preterm birth	Reference	0.08	---	---
Low birthweight	Alternative		-0.479 (-0.518, -0.440)	0.620 (0.596, 0.644)

Appendix Table 33. MR-BRT preterm (<37 weeks gestation) to extremely preterm (<28 weeks gestation)

Data input	Reference or alternative case definition	Gamma	Beta coefficient, log (95% CI)	Adjustment factor (95% UI)*
28 weeks	Reference	0.06	---	---
37 weeks	Alternative		3.221 (3.161, 3.281)	25.053 (23.600, 26.604)

Appendix Table 34. MR-BRT extremely preterm (<28 weeks gestation) to preterm (<37 weeks gestation)

Data input	Reference or alternative case definition	Gamma	Beta coefficient, log (95% CI)	Adjustment factor (95% UI)*
37 weeks	Reference	0.05	---	---
28 weeks	Alternative		-3.208 (-3.266, -3.150)	0.0404 (0.0381, 0.0428)

**MR-BRT crosswalk adjustments can be interpreted as the factor the alternative case definition is adjusted by to reflect what it would have been had it been measured using the reference case definition. If the log/logit beta coefficient is negative, then the alternative is adjusted up to the reference. If the log/logit beta coefficient is positive, then the alternative is adjusted down to the reference.*

***The adjustment factor column is the exponentiated beta coefficient. For log beta coefficients, this is the relative rate between the two case definitions. For logit beta coefficients, this is the relative odds between the two case definitions.*

These data adjustments had the effect of dramatically increasing the size of each of the modelling datasets and are primarily responsible for most changes in preterm estimates between GBD 2019 and GBD 2021. After all crosswalks, we performed a deduplication step on GA models. Namely, if low birthweight data in countries that were 1) categorised as "data-rich" locations in cause-of-death modelling or had at least 10 consecutive years of vital registration data recording gestational age, and 2) had both preterm birth and low birthweight data, then crosswalked low birthweight data were outliered so that the model was informed only by the gestational age data.

Modelling strategy

Step A: Model univariate birthweight and gestational age distributions at birth, by l/y/s

Microdata are the ideal data source for modelling distributions; however, microdata are not widely available for birthweight and are scarcer for gestational age. Categorical prevalence data are more readily available from a wider range of locations and years for low birthweight (<2500g), extremely preterm (<28 weeks of gestation), and preterm birth (<37 weeks of gestation). Because categorical

prevalence has wider availability than microdata, we use prevalence data to assist in modelling birthweight and gestational age ensemble distributions.

Ensemble distribution models can be constructed with three pieces of information: mean of the distribution, variance of the distribution, and the weights of the distributions being used in the ensemble. To model mean and variance for all l/y/s for birthweight and gestational age, we first used spatiotemporal Gaussian process regression (ST-GPR) models to model prevalence of low birthweight, extremely preterm, and preterm birth for all l/y/s at birth. To model mean birthweight for all l/y/s, OLS linear regression was used to regress mean birthweight on log-transformed low birthweight prevalence. This model was then used to predict mean birthweight for all l/y/s, using the prevalence of low birthweight (<2500 grams) modelled for all l/y/s in ST-GPR. Similarly, to model gestational age mean for all l/y/s, OLS linear regression model was used to regress mean gestational age on log-transformed preterm prevalence. Mean gestational age for all l/y/s was predicted using the preterm birth (<37 weeks) estimated modelled in ST-GPR.

Global ensemble weights for gestational age were derived by using all available gestational age and birthweight microdata in Table 14 to select the ensemble weights. The distribution families included in the optimization process were exponential, gamma, gumbel, Weibull, log-normal, normal, mirrored gamma, and mirrored gumbel. As an advancement in GBD 2020, ensemble weights were fit that specifically targeted the fit at 28 weeks and 37 weeks for gestational age and 1500 grams and 2500 grams for low birthweight. In previous GBD cycles the fit of these models had been optimized to reduce error across the entire distribution. Additionally, as an improvement in GBD 2020, this ensemble weight fitting strategy optimized on all microdata sources simultaneously, as opposed to separately.

For each l/y/s, given the mean and ensemble weights, the variance was optimised to minimise error on the prevalence of preterm birth (<37 weeks) for the gestational age distribution and prevalence of low birthweight (<2500 grams) for the birthweight distribution.

Step B: Model joint birthweight and gestational age distributions at birth, by l/y/s

In order to model the joint distribution of gestational age and birthweight from separate distributions, information was needed about the correlation between the two distributions. Distributions of gestational age and birthweight are not independent; the Spearman correlation for each country where joint microdata were available (Table 14), pooling across all years of data available, ranged from 0.25 to 0.49. The overall Spearman correlation was 0.38, pooling across all countries in the dataset.

Appendix Table 35: Summary of microdata inputs

Location	Years of data	Total births*	Format of data	Spearman correlation **	Used in ensemble weight selection	Used in copula parameter selection	Used in relative risk models
BRA	2016	2,854,380	Microdata	0.37	Yes	Yes	No
ECU	2003–2015	2,473,039	Microdata	0.34	Yes	Yes	No
ESP	1990–2014	8,537,220	Microdata	0.42	Yes	Yes	No
JPN	1995–2015	23,644,506	Tabulations	0.41	No	No	Yes
MEX	2008–2012	10,256,117	Microdata	0.35	Yes	Yes	No
NOR	1990–2014	1,489,210	Microdata	0.44	Yes	Yes	Yes
NZL	1990–2016	1,600,501	Microdata	0.25	Yes	Yes	Yes
SGP	1993–2015	972,775	Tabulations	0.41	No	No	Yes
TWN	1998–2002	1,331,760	Tabulations	0.38	No	No	Yes
URY	1996–2014	698,622	Microdata	0.49	Yes	Yes	No

USA	1990–2014	81,929,879	Microdata	0.38	Yes	Yes	Yes

** Pooled across all years and sexes, excluding data missing year of birth, gestational age, or birthweight*

**GBD estimates include uncertainty from a substantial number of different sources, for example relative risk estimation, exposure estimation, and crosswalk beta co-efficients. There are some components, including Spearman correlation, where uncertainty is not captured in final GBD estimates

Joint distributions between the birthweight and gestational age marginal distributions were modelled with copulae. The Copula and VineCopula packages in R were used to select the optimal copula family and copula parameters to model the joint distribution, using joint microdata from the country-years in Table 14. The copula family selected from the microdata was "Survival BB8", with theta parameter set to 1.75 and delta parameter set to 1.

The joint distribution of birthweight and gestational age per location-year-sex was modelled using the global copula family and parameters selected and the location-year-sex gestational age and birthweight distributions. The joint distribution was simulated 100 times to capture uncertainty. Each simulation consisted of 10,000 simulated joint birthweight and gestational age datapoints. Each joint distribution was divided into 500g by 2-week bins to match the categorical bins of the relative risk surface. Birth prevalence was then calculated for each 500g by 2-week bin.

Step C: Model joint distributions from birth to the end of the neonatal period, by l/y/s

Early neonatal prevalence and late neonatal prevalence were estimated using life table approaches for each 500g and 2-week bin. Using the all-cause early neonatal mortality rate for each location-year-sex, births per location-year-sex-bin, and the relative risks for each location-year-sex-bin in the early neonatal period, the all-cause early neonatal mortality rate was calculated for each location-year-sex-bin. The early neonatal mortality rate per bin was used to calculate the number of survivors at seven days and prevalence in the early neonatal period. Using the same process, the all-cause late neonatal mortality rate for each location-year-sex was paired with the number of survivors at seven days and late neonatal relative risks per bin to calculate late neonatal prevalence and survivors at 28 days.

Relative risks and theoretical minimum risk exposure level

LBWSG is paired with the outcomes listed in Table 15 and is only attributed to burden in the early and late neonatal period.

Appendix Table 36: Cause list of outcomes for low birthweight and short gestation

Cause name
Diarrhoeal diseases
Lower respiratory infections
Upper respiratory infections
Otitis media
Pneumococcal meningitis
H influenzae type B meningitis
Meningococcal meningitis
Other meningitis
Encephalitis
Neonatal preterm birth complications
Neonatal encephalopathy due to birth asphyxia and trauma

Neonatal sepsis and other neonatal infections
Haemolytic disease and other neonatal jaundice
Other neonatal disorders
Sudden infant death syndrome

Causes

The available data for deriving relative risk was only for all-cause mortality. The exception was the USA linked infant birth-death cohort data, which contained three-digit ICD causes of death, but also had nearly 30% of deaths coded to causes that are ill-defined, or intermediate, in the GBD cause classification system. We analysed the relative risk of all-cause mortality across all available sources and selected outcomes based on criteria of biological plausibility. Some causes, most notably congenital birth defects, haemoglobinopathies, malaria, and HIV/AIDS, were excluded based on the criteria that reverse causality could not be excluded.

Input data

In the Norway, New Zealand, and USA Linked Birth/Death Cohort microdata datasets, livebirths are reported with gestational age, birthweight, and an indicator of death at 7 days and 28 days. For this analysis, gestational age was grouped into two-week categories, and birthweight was grouped into 500-gram categories. The Taiwan, Japan, and Singapore datasets were prepared in tabulations of joint 500-gram and two-week categories. A pooled country analysis of mortality risk in the early neonatal period and late neonatal period by "small-for-gestational-age" category in developing countries in Asia and sub-Saharan Africa were also used to inform the relative risk analysis.

Appendix Table 37: Input data for relative risk models

Input data	Relative risk
Source count (total)	113
Number of countries with data	6

Modelling strategy

For each location, data were pooled across years, and the risk of all-cause mortality at the early neonatal period and late neonatal period at joint birthweight and gestational age combinations was calculated. In all datasets except for the USA, sex-specific data were combined to maximise sample size. The USA analyses were sex-specific. To calculate relative risk at each 500-gram and two-week combination, logistic regression was first used to calculate mortality odds for each joint two-week gestational age and 500-gram birthweight category. Mortality odds were smoothed with Gaussian process regression, with the independent distributions of mortality odds by birthweight and mortality odds by gestational age serving as priors in the regression.

A pooled country analysis of mortality risk in the early neonatal period and late neonatal period by SGA category in developing countries in Asia and sub-Saharan Africa were also converted into 500-gram and two-week bin mortality odds surfaces. The relative risk surfaces produced from microdata and the Asia and Africa surfaces produced from the pooled country analysis were meta-analysed, resulting in a meta-

analysed mortality odds surface for each location. The meta-analysed mortality odds surface for each location was smoothed using Gaussian process regression and then converted into mortality risk. To calculate mortality relative risks, the risk of each joint two-week gestational age and 500-gram birthweight category were divided by the risk of mortality in the joint gestational age and birthweight category with the lowest mortality risk.

For each of the country-derived relative risk surfaces, the 500-gram and two-week gestational age joint bin with the lowest risk was identified. This bin differed within each country dataset. To identify the universal 500-gram and two-week gestational age category that would serve as the universal TMREL for our analysis, we chose the bins that was identified to be the TMREL in each country dataset to contribute to the universal TMREL. Therefore, the joint categories that served as our universal TMREL for the LBWSG risk factor were “38–40 weeks of gestation and 3500–4000 grams”, “38–40 weeks of gestation and 4000–4500 grams”, and “40–42 weeks of gestation and 4000–4500 grams”. As the joint TMREL, all three categories were assigned to a relative risk equal to 1.

Population attributable fraction

The total PAF for the low birthweight and short gestation joint risk factor was calculated by summing the PAF calculated from each 500g x two-week category, with the lowest risk category among all the 500g x two-week categories serving as the TMREL. The equation for calculating PAF for each 500g x two-week category is:

$$PAF_{joasgt} = \frac{\sum_{x=1}^{u} RR_{joast}(x)P_{jasgt}(x) - RR_{joasg}(TMRE_{jas})}{\sum_{x=1}^{u} RR_{joas}(x)P_{jasgt}(x)}$$

To calculate the PAFs for the univariate risks (‘short gestation for birthweight’ and ‘low birthweight for gestation’), relative risks are first weighted by global exposure in 2019, summed across one of the dimensions (gestational age or birthweight), and then rescaled by the maximum relative risk in the TMREL block (38-42 weeks of gestation and 3500-4500 grams). Any relative risk less than 1 was set to 1. Exposure was also summed across the same dimension, and the univariate PAF equalled the sum of the product of the weighted relative risks and exposures.

Statement of GATHER Compliance

Appendix Table 38. Checklist of information that should be included in reports of global health estimates, with description of compliance and location of information the current study

#	GATHER checklist item	Description of compliance	Reference
**Objectives and funding**			
1	Define the indicators, populations, and time periods for which estimates were made.	Narrative provided in paper and methods appendix describing indicators, definitions, and populations	Main text (Methods) and methods appendix
2	List the funding sources for the work.	Funding sources listed in paper	Summary (Funding)
**Data Inputs**			
For all data inputs from multiple sources that are synthesized as part of the study:			
3	Describe how the data were identified and how the data were accessed.	Narrative description of data seeking methods provided	Main text (Methods) and methods appendix
4	Specify the inclusion and exclusion criteria. Identify all ad-hoc exclusions.	Narrative about inclusion and exclusion criteria by data type provided	Main text (Methods) and methods appendix
5	Provide information on all included data sources and their main characteristics. For each data source used, report reference information or contact name/institution, population represented, data collection method, year(s) of data collection, sex and age range, diagnostic criteria or measurement method, and sample size, as relevant.	An interactive, online data source tool that provides metadata for data sources by component, geography, cause, risk, or impairment has been developed	Online data citation tool, http://ghdx.healthdata.org/gbd-2021
6	Identify and describe any categories of input data that have potentially important biases (e.g., based on characteristics listed in item 5).	Summary of known biases by cause included in methods appendix	Main text (Methods) and Methods appendix
For data inputs that contribute to the analysis but were not synthesized as part of the study:			

7	Describe and give sources for any other data inputs.	Included in online data source tool, http://ghdx.healthdata.org/gbd-2021 (link will be live upon publication)	Online data citation tools
For all data inputs:			
8	Provide all data inputs in a file format from which data can be efficiently extracted (e.g., a spreadsheet as opposed to a PDF), including all relevant meta-data listed in item 5. For any data inputs that cannot be shared due to ethical or legal	Downloads of input data available through online tools, including data visualization tools	Online data visualization tool, http://ghdx.healthdata.org/gbd-2021

Formatted: Font color: Auto

References

1. GBD 2021 Disability-Adjusted Life Years (DALYs) Collaborators. Global incidence, prevalence, years lived with disability (YLDs), disability-adjusted life-years (DALYs), and healthy life expectancy (HALE) for 371 diseases and injuries in 204 countries and territories and 811 subnational locations, 1990–2021: A systematic analysis for the Global Burden of Disease Study 2021. The Lancet (under review).

2. Naghavi M, Ong KL, Aali A, Ababneh HS, Abate YH, Abbafati C, et al. Global burden of 288 causes of death and life expectancy decomposition in 204 countries and territories and 811 subnational locations, 1990–2021: a systematic analysis for the Global Burden of Disease Study 2021. The Lancet [Internet]. 2024 Apr 3 [cited 2024 Apr 15];0(0). Available from: https://www.thelancet.com/journals/lancet/article/PIIS0140-6736(24)00367-2/fulltext

3. GBD 2021 Risk Factors Collaborators. Global burden and strength of evidence for 88 risk factors in 204 countries, 1990–2021: a systematic analysis for the Global Burden of Disease Study 2021. Lancet (in review).

4. Lin YS, Lin LC, Lee FP, Lee KJ. The prevalence of chronic otitis media and its complication rates in teenagers and adult patients. Otolaryngology - Head and Neck Surgery. 2009 Feb 1;140(2):165–70.

5. Hammer MS, van Donkelaar A, Li C, Lyapustin A, Sayer AM, Hsu NC, et al. Global Estimates and Long-Term Trends of Fine Particulate Matter Concentrations (1998-2018). Environ Sci Technol. 2020 Jul 7;54(13):7879–90.

6. van Donkelaar A, Martin RV, Brauer M, Hsu NC, Kahn RA, Levy RC, et al. Global Estimates of Fine Particulate Matter using a Combined Geophysical-Statistical Method with Information from Satellites, Models, and Monitors. Environ Sci Technol. 2016 Apr 5;50(7):3762–72.

7. Shaddick G, Thomas ML, Green A, Brauer M, Donkelaar A, Burnett R, et al. Data Integration Model for Air Quality: A Hierarchical Approach to the Global Estimation of Exposures to Ambient Air Pollution. Journal of the Royal Statistical Society Series C: Applied Statistics. 2018 Jan 1;67(1):231–53.

8. Shaddick G, Thomas ML, Mudu P, Ruggeri G, Gumy S. Half the world’s population are exposed to increasing air pollution. npj Clim Atmos Sci. 2020 Jun 17;3(1):1–5.

9. Brauer M, Freedman G, Frostad J, van Donkelaar A, Martin RV, Dentener F, et al. Ambient Air Pollution Exposure Estimation for the Global Burden of Disease 2013. Environ Sci Technol. 2016 Jan 5;50(1):79–88.

10. Rue H, Martino S, Chopin N. Approximate Bayesian Inference for Latent Gaussian models by using Integrated Nested Laplace Approximations. Journal of the Royal Statistical Society Series B: Statistical Methodology. 2009 Apr 1;71(2):319–92.

11. Thomas ML, Shaddick G, Simpson D, de Hoogh K, Zidek JV. Spatio-temporal downscaling for continental scale estimation of air pollution concentrations. Journal of the Royal Statistical Society: Series C. 2020;Submitted.

12. Turner MC, Jerrett M, Pope CA, Krewski D, Gapstur SM, Diver WR, et al. Long-Term Ozone Exposure and Mortality in a Large Prospective Study. Am J Respir Crit Care Med. 2016 May 15;193(10):1134–42.

13. Yin P, Brauer M, Cohen A, Burnett RT, Liu J, Liu Y, et al. Long-term Fine Particulate Matter Exposure and Nonaccidental and Cause-specific Mortality in a Large National Cohort of Chinese Men. Environ Health Perspect. 2017 Nov 7;125(11):117002.

14. Li T, Zhang Y, Wang J, Xu D, Yin Z, Chen H, et al. All-cause mortality risk associated with long-term exposure to ambient PM2·5 in China: a cohort study. The Lancet Public Health. 2018 Oct 1;3(10):e470–7.

15. Yang Y, Tang R, Qiu H, Lai PC, Wong P, Thach TQ, et al. Long term exposure to air pollution and mortality in an elderly cohort in Hong Kong. Environ Int. 2018 Aug;117:99–106.

16. Hystad P, Larkin A, Rangarajan S, AlHabib KF, Avezum Á, Calik KBT, et al. Associations of outdoor fine particulate air pollution and cardiovascular disease in 157 436 individuals from 21 high-income, middle-income, and low-income countries (PURE): a prospective cohort study. Lancet Planet Health. 2020 Jun;4(6):e235–45.

17. Yusuf S, Joseph P, Rangarajan S, Islam S, Mente A, Hystad P, et al. Modifiable risk factors, cardiovascular disease, and mortality in 155 722 individuals from 21 high-income, middle-income, and low-income countries (PURE): a prospective cohort study. Lancet. 2020 Mar 7;395(10226):795–808.

18. Burnett RT, Pope CA, Ezzati M, Olives C, Lim SS, Mehta S, et al. An integrated risk function for estimating the global burden of disease attributable to ambient fine particulate matter exposure. Environ Health Perspect. 2014 Apr;122(4):397–403.

19. Pope CA, Cohen AJ, Burnett RT. Cardiovascular Disease and Fine Particulate Matter: Lessons and Limitations of an Integrated Exposure Response Approach. Circ Res. 2018 Jun 8;122(12):1645–7.

20. Lind L, Sundström J, Ärnlöv J, Lampa E. Impact of Aging on the Strength of Cardiovascular Risk Factors: A Longitudinal Study Over 40 Years. J Am Heart Assoc. 2018 Jan 6;7(1):e007061.

21. Ghosh R, Causey K, Burkart K, Wozniak S, Cohen A, Brauer M. Ambient and household PM2.5 pollution and adverse perinatal outcomes: A meta-regression and analysis of attributable global burden for 204 countries and territories. PLOS Medicine. 2021 Sep 28;18(9):e1003718.

22. US EPA NATIONAL CENTER FOR ENVIRONMENTAL ASSESSMENT RTPN, Sacks J. Integrated Science Assessment (ISA) for Particulate Matter (Final Report, Dec 2009) [Internet]. [cited 2024 Apr 15]. Available from: https://cfpub.epa.gov/ncea/risk/recordisplay.cfm?deid=216546

23. World Health Organization. Review of evidence on health aspects of air pollution – REVIHAAP Project technical report. Copenhagen: WHO Regional Office for Europe [Internet]. 2013. Available from: https://iris.who.int/bitstream/handle/10665/341712/WHO-EURO-2013-4101-43860-61757-eng.pdf?sequence=1

24. World Health Organization (WHO). WHO Household Energy Database 1960-2017. In Geneva, Switzerland: World Health Organization (WHO); 2019.

25. Smith KR, Bruce N, Balakrishnan K, Adair-Rohani H, Balmes J, Chafe Z, et al. Millions dead: how do we know and what does it mean? Methods used in the comparative risk assessment of household air pollution. Annu Rev Public Health. 2014;35:185–206.

26. Tanchangya J, Geater AF. Use of traditional cooking fuels and the risk of young adult cataract in rural Bangladesh: a hospital-based case-control study. BMC Ophthalmology. 2011 Jun 16;11(1):16.

27. Shupler M, Balakrishnan K, Ghosh S, Thangavel G, Stroud-Drinkwater S, Adair-Rohani H, et al. Global household air pollution database: Kitchen concentrations and personal exposures of particulate matter and carbon monoxide. Data Brief. 2018 Dec;21:1292–5.

28. Shupler M, Hystad P, Birch A, Miller-Lionberg D, Jeronimo M, Arku RE, et al. Household and personal air pollution exposure measurements from 120 communities in eight countries: results from the PURE-AIR study. The Lancet Planetary Health. 2020 Oct 1;4(10):e451–62.
