# Supplementary material for: Global, regional, and national burden of upper respiratory infections and otitis media, 1990–2021: a systematic analysis from the Global Burden of Disease Study 2021
Source: Lancet Infect Dis. 2025 Jan;25(1):36–51. doi: 10.1016/S1473-3099(24)00430-4 (PMC11680489; doi:10.1016/S1473-3099(24)00430-4)
Supplement: Supplementary appendix 3 [file mmc3.pdf]

THE LANCET
Infectious Diseases

Supplementary appendix 3

This appendix formed part of the original submission and has been peer reviewed. We post it as supplied by the authors.

Supplement to: GBD 2021 Upper Respiratory Infections and Otitis Media Collaborators. Global, regional, and national burden of upper respiratory infections and otitis media, 1990–2021: a systematic analysis from the Global Burden of Disease Study 2021. *Lancet Infect Dis* 2024; published online Sept 9. https://doi.org/10.1016/S1473-3099(24)00430-4.

Appendix 3: Authorship appendix to "Global, regional, and national burden of upper respiratory infections and otitis media, 1990–2021: results from the Global Burden of Disease Study 2021"

This appendix provides further authorship detail for "Global, regional, and national burden of upper respiratory infections and otitis media, 1990–2021: results from the Global Burden of Disease Study 2021"

Table of Contents

GBD 2021 Upper Respiratory Infection & Otitis Media Collaborators 2

Affiliations 4

Authors' Contributions.. 18

Managing the overall research enterprise.. 18

Writing the first draft of the manuscript 18

Primary responsibility for applying analytical methods to produce estimates 18

Primary responsibility for seeking, cataloguing, extracting, or cleaning data; designing or coding figures and tables.. 18

Providing data or critical feedback on data sources.. 18

Developing methods or computational machinery 19

Providing critical feedback on methods or results 20

Drafting the work or revising it critically for important intellectual content 22

Managing the estimation or publications process.. 23

GBD 2021 Upper Respiratory Infection & Otitis Media Collaborators

Sarah Brooke Sirota,* Matthew C Doxey,* Regina-Mae Villanueva Dominguez, Rose Grace Bender, Avina Vongpradith, Samuel B Albertson, Amanda Novotney, Katrin Burkart, Austin Carter, Parsa Abdi, Meriem Abdoun, Ayele Mamo Abebe, Kedir Hussein Abegaz, Richard Gyan Aboagye, Hassan Abolhassani, Lucas Guimarães Abreu, Hasan Abualruz, Eman Abu-Gharbieh, Salahdein Aburuz, Mesafint Molla Adane, Isaac Yeboah Addo, Victor Adekanmbi, Qorinah Estiningtyas Sakilah Adnani, Leticia Akua Adzigbli, Muhammad Sohail Afzal, Saira Afzal, Bright Opoku Ahinkorah, Sajjad Ahmad, Ayman Ahmed, Haroon Ahmed, Syed Anees Ahmed, Karolina Akinosoglou, Mohammed Ahmed Akkaif, Salah Al Awaidy, Samer O Alalalmeh, Mohammed Albashtawy, Mohammad T AlBataineh, Adel Ali Saeed Al-Gheethi, Fadwa Naji Alhalaiqa, Robert Kaba Alhassan, Abid Ali, Liaqat Ali, Mohammed Usman Ali, Syed Shujait Ali, Waad Ali, Joseph Uy Almazan, Jaber S Alqahtani, Ahmad Alrawashdeh, Rami H Al-Rifai, Najim Z Alshahrani, Khaled Altartoor, Jaffar A Al-Tawfiq, Nelson Alvis-Guzman, Yaser Mohammed Al-Worafi, Hany Aly, Safwat Aly, Karem H Alzoubi, Walid Adnan Al-Zyoud, Abebe Feyissa Amhare, Hubert Amu, Ganiyu Adeniyi Amusa, Abhishek Anil, Saeid Anvari, Ekenedilichukwu Emmanuel Anyabolo, Jalal Arabloo, Mosab Arafat, Demelash Areda, Brhane Berhe Aregawi, Abdulfatai Aremu, Seyyed Shamsadin Athari, Avinash Aujayeb, Zewdu Bishaw Aynalem, Sina Azadnajafabad, Ahmed Y Azzam, Muhammad Badar, Pegah Bahrami Taghanaki, Saeed Bahramian, Atif Amin Baig, Milica Bajcetic, Senthilkumar Balakrishnan, Maciej Banach, Mainak Bardhan, Hiba Jawdat Barqawi, Mohammad-Mahdi Bastan, Kavita Batra, Ravi Batra, Amir Hossein Behnoush, Maryam Beiranvand, Alemu Gedefie Belete, Melaku Ashagrie Belete, Apostolos Beloukas, Azizullah Beran, Pankaj Bhardwaj, Ashish Bhargava, Ajay Nagesh Bhat, Mohiuddin Ahmed Bhuiyan, Veera R Bitra, Aadam Olalekan Bodunrin, Eyob Ketema Bogale, Sri Harsha Boppana, Hamed Borhany, Souad Bouaoud, Colin Stewart Brown, Danilo Buonsenso, Yasser Bustanji, Luis Alberto Cámera, Carlos A Castañeda-Orjuela, Luca Cegolon, Muthia Cenderadewi, Sandip Chakraborty, Vijay Kumar Chattu, Esther T W Cheng, Fatemeh Chichagi, Patrick R Ching, Hitesh Chopra, Sonali Gajanan Choudhari, Devasahayam J Christopher, Dinh-Toi Chu, Isaac Sunday Chukwu, Erin Chung, Alexandru Corlateanu, Natalia Cruz-Martins, Sriharsha Dadana, Omid Dadras, Tukur Dahiru, Xiaochen Dai, Jai K Das, Nihar Ranjan Dash, Mohsen Dashti, Mohadese Dashtkoohi, Fernando Pio De la Hoz, Shayom Debopadhaya, Berecha Hundessa Demessa, Asmamaw Bizuneh Demis, Vinoth Gnana Chellaiyan Devanbu, Devananda Devegowda, Kuldeep Dhama, Vishal R Dhulipala, Daniel Diaz, Michael J Diaz, Thanh Chi Do, Thao Huynh Phuong Do, Masoud Dodangeh, Fariba Dorostkar, Ashel Chelsea Dsouza, Haneil Larson Dsouza, Senbagam Duraisamy, Oyewole Christopher Durojaiye, Arkadiusz Marian Dziedzic, Abdelaziz Ed-Dra, Michael Ekholuenetale, Temitope Cyrus Ekundayo, Iman El Sayed, Faris El-Dahiyat, Muhammed Elhadi, Mohammed Elshaer, Majid Eslami, Ugochukwu Anthony Eze, Adeniyi Francis Fagbamigbe, Ali Faramarzi, Folorunso Oludayo Fasina, Nuno Ferreira, Florian Fischer, Ida Fitriana, Luisa S Flor, Santosh Gaihre, Márió Gajdács, Nasrin Galehdar, Mohammad Arfat Ganiyani, Miglas Welay Gebregergis, Mesfin Gebrehiwot, Teferi Gebru Gebremeskel, Genanew K Getahun, Molla Getie, Keyghobad Ghadiri, Afsaneh Ghasemzadeh, Mahsa Ghorbani, Mohamad Goldust, Mahaveer Golechha, Pouya Goleij, Giuseppe Gorini, Anmol Goyal, Shi-Yang Guan, Giovanni Guarducci, Mesay Dechasa Gudeta, Renu Gupta, Sapna Gupta, Veer Bala Gupta, Vivek Kumar Gupta, Mostafa Hadei, Najah R Hadi, Arvin Haj-Mirzaian, Rabih Halwani, Samer Hamidi, Ahmad Hammoud, Nasrin Hanifi, Fahad Hanna, Zaim Anan Haq, Md Rabiul Haque, S M Mahmudul Hasan, Hamidreza Hasani, Md Saquib Hasnain, Hadi Hassankhani, Johannes Haubold, Khezar Hayat, Omar E Hegazi, Kamal Hezam, Ramesh Holla, Praveen Hoogar, Nobuyuki Horita, Mihaela Hostiuc, Hong-Han Huynh, Segun Emmanuel Ibitoye, Olayinka Stephen Ilesanmi, Irena M Ilic, Milena D Ilic,

Mohammad Tarique Imam, Mustafa Alhaji Isa, Md Rabiul Islam, Sheikh Mohammed Shariful Islam, Nahlah Elkudssiah Ismail, Masao Iwagami, Vinothini J, Abdollah Jafarzadeh, Khushleen Jaggi, Ammar Abdulrahman Jairoun, Mihajlo Jakovljevic, Elham Jamshidi, Shubha Jayaram, Bijay Mukesh Jeswani, Ravi Prakash Jha, Jobinse Jose, Nitin Joseph, Charity Ehimwenma Joshua, Jacek Jerzy Jozwiak, Vaishali K, Zubair Kabir, Himal Kandel, Kehinde Kazeem Kanmodi, Surya Kant, Rami S Kantar, Ibraheem M Karaye, Arman Karimi Behnagh, Navjot Kaur, Himanshu Khajuria, Amirmohammad Khalaji, Faham Khamesipour, Gulfaraz Khan, M Nuruzzaman Khan, Maseer Khan, Mohammad Jobair Khan, Min Seo Kim, Ruth W Kimokoti, Sonali Kochhar, Vladimir Andreevich Korshunov, Soewarta Kosen, Kewal Krishan, Hare Krishna, Vijay Krishnamoorthy, Barthelemy Kuate Defo, Md Abdul Kuddus, Mohammed Kuddus, Ilari Kuitunen, Mukhtar Kulimbet, Dewesh Kumar, Om P Kurmi, L V Simhachalam Kutikuppala, Chandrakant Lahariya, Dharmesh Kumar Lal, Savita Lasrado, Kaveh Latifinaibin, Huu-Hoai Le, Nhi Huu Hanh Le, Thao Thi Thu Le, Trang Diep Thanh Le, Seung Won Lee, Wei-Chen Lee, Ming-Chieh Li, Peng Li, Stephen S Lim, Gang Liu, Runben Liu, Wei Liu, Xiaofeng Liu, Xuefeng Liu, László Lorenzovici, Lisha Luo, Azeem Majeed, Elaheh Malakan Rad, Kashish Malhotra, Iram Malik, Aseer Manilal, Bharati Mehta, Tesfahun Mekene Meto, Mathewos M Mekonnen, Hadush Negash Meles, Ziad Ahmed Memish, Max Alberto Mendez-Lopez, Sultan Ayoub Meo, Mohsen Merati, Tomislav Mestrovic, Sachith Mettananda, Le Huu Nhat Minh, Erkin M Mirrakhimov, Arup Kumar Misra, Ahmed Ismail Mohamed, Nouh Saad Mohamed, Mesud Mohammed, Mustapha Mohammed, Ali H Mokdad, Lorenzo Monasta, Mohammad Ali Moni, AmirAli Moodi Ghalibaf, Catrin E Moore, Lidia Morawska, Rohith Motappa, Vincent Mougin, Parsa Mousavi, Ghulam Mustafa, Pirouz Naghavi, Ganesh R Naik, Firzan Nainu, Mohammad Sadeq Najafi, Soroush Najdaghi, Hastyar Hama Rashid Najmuldeen, Shumaila Nargus, Delaram Narimani Davani, Mohammad Naser, Zuhair S Natto, Biswa Prakash Nayak, Seyed Aria Nejadghaderi, Dang H Nguyen, Hau Thi Hien Nguyen, Van Thanh Nguyen, Taxiarchis Konstantinos Nikolouzakis, Efaq Ali Noman, Chisom Adaobi Nri-Ezedi, Virginia Nuñez-Samudio, Vincent Ebuka Nwatah, Ismail A Odetokun, Akinkunmi Paul Okekunle, Osaretin Christabel Okonji, Patrick Godwin Okwute, Titilope O Olanipekun, Isaac Iyinoluwa Olufadewa, Bolajoko Olubukunola Olusanya, Goran Latif Omer, Kenneth Ikenna Onyedibe, Michal Ordak, Verner N Orish, Esteban Ortiz-Prado, Nikita Otstavnov, Amel Ouyahia, Mahesh Padukudru P A, Jagadish Rao Padubidri, Ashok Pandey, Ioannis Pantazopoulos, Shahina Pardhan, Pragyan Paramita Parija, Romil R Parikh, Seoyeon Park, Ashwaghosha Parthasarathi, Maja Pasovic, Aslam Ramjan Pathan, Shankargouda Patil, Shrikant Pawar, Prince Peprah, Arokiasamy Perianayagam, Dhayaneethie Perumal, Ionela-Roxana Petcu, Hoang Nhat Pham, Hoang Tran Pham, Anil K Philip, David M Pigott, Zahra Zahid Piracha, Dimitri Poddighe, Roman V Polibin, Maarten J Postma, Reza Pourbabaki, Elton Junio Sady Prates, Jagadeesh Puvvula, Asma Saleem Qazi, Gangzhen Qian, Quinn Rafferty, Fakher Rahim, Mehran Rahimi, Vafa Rahimi-Movaghar, Md Obaidur Rahman, Mosiur Rahman, Muhammad Aziz Rahman, Mohammad Rahmanian, Nazanin Rahmanian, Vahid Rahmanian, Masoud Rahmati, Prashant Rajput, Mahmoud Mohammed Ramadan, Shakthi Kumaran Ramasamy, Pushkal Sinduvadi Ramesh, Indu Ramachandra Rao, Mithun Rao, Sowmya J Rao, Sina Rashedi, Mohammad-Mahdi Rashidi, Devarajan Rathish, Nakul Ravikumar, Salman Rawaf, Elrashdy Moustafa Mohamed Redwan, Luis Felipe Felipe Reyes, Nazila Rezaei, Nima Rezaei, Omid Rezahosseini, Syed Mohd Danish Rizvi, Jefferson Antonio Buendia Rodriguez, Luca Ronfani, Shekoufeh Roudashti, Priyanka Roy, Guilherme de Andrade Ruela, Basema Ahmad Saddik, Mohammad Reza Saeb, Umar Saeed, Pooya Saeedi, Mehdi Safari, Fatemeh Saheb Sharif-Askari, Narjes Saheb Sharif-Askari, Amirhossein Sahebkar, Monalisha Sahu, Joseph W Sakshaug, Nasir Salam, Afeez Abolarinwa Salami, Mohamed A Saleh, Malik Sallam, Yoseph Leonardo Samodra, Rama Krishna Sanjeev, Milena M Santric-Milicevic, Aswini Saravanan, Benn Sartorius, Anudeep Sathyanarayan, Jennifer Saulam,

Sonia Saxena, Ganesh Kumar Saya, Benedikt Michael Schaarschmidt, Austin E Schumacher, Mansour Sedighi, Ashenafi Kibret Sendekie, Subramanian Senthilkumaran, Yashendra Sethi, SeyedAhmad SeyedAlinaghi, Mahan Shafie, Samiah Shahid, Masood Ali Shaikh, Sunder Sham, Mohammad Ali Shamshirgaran, Mohd Shanawaz, Mohammed Shannawaz, Amin Sharifan, Javad Sharifi-Rad, Rajesh P Shastry, Aziz Sheikh, Mika Shigematsu, Rahman Shiri, Aminu Shittu, Ivy Shiue, Seyed Afshin Shorofi, Emmanuel Edwar Siddig, Colin R Simpson, Jasvinder A Singh, Paramdeep Singh, Surjit Singh, Robert Sinto, Ranjan Solanki, Sameh S M Soliman, Muhammad Suleman, Rizwan Suliankatchi Abdulkader, Chandan Kumar Swain, Lukasz Szarpak, Seyyed Mohammad Tabatabaei, Mohammad Tabish, Zanan Mohammed-Ameen Taha, Jabeen Taiba, Iman M Talaat, Jacques Lukenze Tamuzi, Birhan Tsegaw Taye, Yibekal Manaye Tefera, Mohamad-Hani Temsah, Dufera Rikitu Terefa, Ramna Thakur, Rekha Thapar, Sathish Thirunavukkarasu, Ales Tichopad, Jansje Henny Vera Ticoalu, Marcos Roberto Tovani-Palone, Nghia Minh Tran, Ngoc Ha Tran, Nguyen Tran Minh Duc, Guesh Mebrahtom Tsegay, Munkhtuya Tumurkhuu, Aniefiok John Udoakang, Era Upadhyay, Seyed Mohammad Vahabi, Asokan Govindaraj Vaithinathan, Rohollah Valizadeh, Tommi Juhani Vasankari, Manish Vinayak, Muhammad Waqas, Haftom Legese Weldetinsaa, Nuwan Darshana Wickramasinghe, Ali Yadollahpour, Sajad Yaghoubi, Saber Yezli, Dehui Yin, Dong Keon Yon, Naohiro Yonemoto, Yong Yu, Fathiah Zakham, Ghazal G Z Zandieh, Iman Zare, Fatemeh Zarimeidani, Michael Zastrozhin, Chunxia Zhai, Haijun Zhang, Zhi-Jiang Zhang, Yang Zhao, Juexiao Zhou, Hafsa Zia, Magdalena Zielińska, Mohammad Zoladl, Samer H Zyoud, Aleksandr Y Aravkin, Nicholas J Kassebaum, Mohsen Naghavi, Theo Vos, Simon I Hay, Christopher J L Murray, and Hmwe Hmwe Kyu.

*co-first author

Affiliations

Institute for Health Metrics and Evaluation (S B Sirota MA, R V Dominguez BS, R G Bender MSc, A Vongpradith BA, S B Albertson BS, A Novotney MPH, K Burkart PhD, A Carter MPH, E Chung MD, X Dai PhD, L S Flor MPH, Prof S S Lim PhD, T Mestrovic PhD, A H Mokdad PhD, V Mougin BA, M Pasovic MEd, D M Pigott PhD, Q Rafferty BA, A E Schumacher PhD, A Y Aravkin PhD, N J Kassebaum MD, Prof M Naghavi PhD, Prof T Vos PhD, Prof S I Hay FMedSci, Prof C J L Murray DPhil, H H Kyu PhD), Department of Health Metrics Sciences, School of Medicine (K Burkart PhD, X Dai PhD, L S Flor MPH, Prof S S Lim PhD, A H Mokdad PhD, D M Pigott PhD, B Sartorius PhD, A Y Aravkin PhD, N J Kassebaum MD, Prof M Naghavi PhD, Prof T Vos PhD, Prof S I Hay FMedSci, Prof C J L Murray DPhil, H H Kyu PhD), Department of Pediatrics (E Chung MD), Department of Global Health (S Kochhar MD), Department of Anesthesiology & Pain Medicine (V Krishnamoorthy MD, N J Kassebaum MD), Department of Epidemiology (H Zia BDS), Department of Applied Mathematics (A Y Aravkin PhD), University of Washington, Seattle, WA, USA; Urban Indian Health Institute (M C Doxey MPH), Seattle Indian Health Board, Seattle, WA, USA; School of Medicine (R G Bender MSc), Department of Dermatology (M Goldust MD), Department of Radiology and Biomedical Imaging (X Liu PhD), Department of Genetics (S Pawar PhD), Yale University, New Haven, CT, USA; Department of Medicine (P Abdi BEng), Memorial University, St. John's, NL, Canada; Department of Medicine (Prof M Abdoun PhD), University of Setif Algeria, Sétif, Algeria; Department of Health, Sétif, Algeria (Prof M Abdoun PhD); Pediatrics Nursing Department (A M Abebe MSc), School of Nursing and Midwifery (B Taye MSc), Debre Berhan University, Debre Berhan, Ethiopia; Department of Biostatistics (K H Abegaz PhD), Department of Pharmacy (M Mohammed MSc), Madda Walabu University, Bale Robe, Ethiopia; Department of Family and Community Health (R G Aboagye MPH), Department of Epidemiology and Biostatistics (L A Adzigbli BSc), Institute of Health Research (R K

Alhassan PhD), Department of Population and Behavioural Sciences (H Amu PhD), Department of Microbiology and Immunology (Prof V N Orish PhD), University of Health and Allied Sciences, Ho, Ghana; Research Center for Immunodeficiencies (H Abolhassani PhD, Prof N Rezaei PhD), Non-communicable Diseases Research Center (M Bastan MD, P Mousavi MD, M Rashidi MD, N Rezaei MD), School of Medicine (A Behnoush BS, A Khalaji MD, M Merati MD), Department of Scientific Research (F Chichagi MD), Iranian Research Center for HIV/AIDS (IRCHA) (O Dadras PhD), Department of Obstetrics and Gynecology (M Dashtkoohi MD), Department of Health in Emergencies and Disasters (M Hadei PhD), Department of Pediatric Cardiology (Prof E Malakan Rad MD), Tehran Heart Center (M Najafi MD), Research Center for Advanced Technologies in Cardiovascular Medicine (M Najafi MD), Sina Trauma and Surgery Research Center (Prof V Rahimi-Movaghar MD), Iranian Research Center for HIV/AIDS (Prof S SeyedAlinaghi PhD), Department of Neurology (M Shafie MD), Sina Hospital (A Sharifan PharmD), Research Center for Rational Use of Drugs (A Sharifan PharmD), Faculty of Medicine (S Vahabi MD), Tehran University of Medical Sciences, Tehran, Iran; Department of Medical Biochemistry and Biophysics (H Abolhassani PhD), Karolinska Institute, Stockholm, Sweden; Department of Pediatric Dentistry of the School of Dentistry (Prof L Abreu PhD), Department of Maternal-Child Nursing and Public Health (E J S Prates BS), Federal University of Minas Gerais, Belo Horizonte, Brazil; Department of Nursing (H Abualruz PhD), Al Zaytoonah University of Jordan, Amman, Jordan; Department of Biopharmaceutics and Clinical Pharmacy (Prof E Abu-Gharbieh PhD), School of Pharmacy (Prof Y Bustanji PhD), Department of Pathology, Microbiology and Forensic Medicine (M Sallam PhD), Department of Clinical Laboratories and Forensic Medicine (M Sallam PhD), The University of Jordan, Amman, Jordan; Clinical Sciences Department (Prof E Abu-Gharbieh PhD, H J Barqawi MPhil, N R Dash MD, Prof R Halwani PhD, Prof M M Ramadan PhD, N Saheb Sharif-Askari PhD, Prof I M Talaat PhD), Department of Pharmacy Practice and Pharmacotherapeutics (Prof K H Alzoubi PhD), Department of Basic Biomedical Sciences (Prof Y Bustanji PhD), College of Medicine (Prof R Halwani PhD, Prof B A Saddik PhD, Prof M A Saleh PhD), Sharjah Institute of Medical Sciences (F Saheb Sharif-Askari PhD), Department of Medicinal Chemistry (S S M Soliman PhD), University of Sharjah, Sharjah, United Arab Emirates; Department of Therapeutics (Prof S Aburuz PhD), Institute of Public Health (R H Al-Rifai PhD), Department of Medical Microbiology & Immunology (Prof G Khan PhD), United Arab Emirates University, Al Ain, United Arab Emirates; College of Pharmacy (Prof S Aburuz PhD), University of Jordan, Amman, Jordan; College of Medicine and Health Sciences (M M Adane PhD), Department of Health Promotion and Behavioural Science (E K Bogale MPH), Bahir Dar University, Bahir Dar, Ethiopia; Centre for Social Research in Health (I Y Addo PhD), School of Population Health, Faculty of Medicine and Health (Prof B A Saddik PhD), University of New South Wales, Sydney, NSW, Australia; Quality and Systems Performance Unit (I Y Addo PhD), Cancer Institute NSW, Sydney, NSW, Australia; Department of Obstetrics and Gynecology (V Adekanmbi PhD), The Department of Family Medicine (W Lee PhD), University of Texas Medical Branch, Galveston, TX, USA; Department of Public Health (Q Adnani PhD), Center of Excellence in Higher Education for Pharmaceutical Care Innovation (Prof M J Postma PhD), Universitas Padjadjaran (Padjadjaran University), Bandung, Indonesia; Department of Life Sciences (M S Afzal PhD), University of Management and Technology, Lahore, Pakistan; Department of Community Medicine (Prof S Afzal PhD), King Edward Memorial Hospital, Lahore, Pakistan; Department of Public Health (Prof S Afzal PhD), Public Health Institute, Lahore, Pakistan; School of Public Health (B O Ahinkorah MPhil), School of Life Sciences (G Liu PhD), School of Biomedical Engineering (N Tran MD), University of Technology Sydney, Sydney, NSW, Australia; Department of Health and Biological Sciences (S Ahmad PhD), Abasyn University, Peshawar, Pakistan; Department of Natural Sciences (S Ahmad PhD), Lebanese American University,

Beirut, Lebanon; Institute of Endemic Diseases (A Ahmed MSc), Unit of Basic Medical Sciences (E E Siddig MD), University of Khartoum, Khartoum, Sudan; Swiss Tropical and Public Health Institute (A Ahmed MSc), University of Basel, Basel, Switzerland; Department of Biosciences (H Ahmed PhD), COMSATS Institute of Information Technology, Islamabad, Pakistan; Brody School of Medicine (S Ahmed PhD), Department of Computer Science (A O Bodunrin MSc), Department of Physiology (M Tumurkhuu PhD), East Carolina University, Greenville, NC, USA; Department of Internal Medicine (K Akinosoglou PhD), University of Patras, Patras, Greece; Department of Internal Medicine and Infectious Diseases (K Akinosoglou PhD), University General Hospital of Patras, Patras, Greece; Department of Cardiology (M Akkaif PhD), Fudan University, Shanghai, China; Department of Communicable Diseases (S Al Awaidy MSc), Ministry of Health, Muscat, Oman; Middle East, Eurasia, and Africa Influenza Stakeholders Network, Muscat, Oman (S Al Awaidy MSc); Department of Clinical Sciences (S O Alalalmeh BPharm, O E Hegazi BPharm), Center for Medical and Bio-Allied Health Sciences Research (S H Zyoud PhD), Ajman University, Ajman, United Arab Emirates; Department of Community and Mental Health (Prof M Albashtawy PhD), Al al-Bayt University, Mafraq, Jordan; Faculty of Medicine (Prof M T AlBataineh PhD), Yarmouk University, Irbid, Jordan; Global Centre for Environmental Remediation (A A S Al-Gheethi PhD), University of Newcastle, Newcastle, NSW, Australia; Cooperative Research Centre for Contamination Assessment and Remediation of the Environment, Newcastle, NSW, Australia (A A S Al-Gheethi PhD); College of Nursing (Prof F N Alhalaiqa PhD), QU Health (M Mohammed PhD), Social and Economic Survey Research Institute (Prof A Perianayagam PhD), Qatar University, Doha, Qatar; Psychological Sciences Association, Amman, Jordan (Prof F N Alhalaiqa PhD); Department of Zoology (A Ali PhD), Abdul Wali Khan University Mardan, Mardan, Pakistan; Department of Biological Sciences (L Ali PhD, A S Qazi PhD), National University of Medical Sciences (NUMS), Rawalpindi, Pakistan; Department of Medical Rehabilitation (Physiotherapy) (M U Ali MSc), Department of Microbiology (M A Isa PhD), University of Maiduguri, Maiduguri, Nigeria; Department of Rehabilitation Sciences (M U Ali MSc, M Khan MPH), Hong Kong Polytechnic University, Hong Kong, China; Center for Biotechnology and Microbiology (S S Ali PhD, M Suleman PhD), University of Swat, Swat, Pakistan; Department of Geography (W Ali PhD), Sultan Qaboos University, Muscat, Oman; Department of Medicine (J U Almazan PhD, Prof D Poddighe PhD), Nazarbayev University, Astana, Kazakhstan; Department of Respiratory Care (J S Alqahtani PhD), Prince Sultan Military College of Health Sciences, Dammam, Saudi Arabia; Department of Allied Medical Sciences (A Alrawashdeh PhD), Department of Clinical Pharmacy (Prof K H Alzoubi PhD), Jordan University of Science and Technology, Irbid, Jordan; Department of Family and Community Medicine (N Z Alshahrani MD), University of Jeddah, Jeddah, Saudi Arabia; Otolaryngology Department (K Altartoor MD), Department of Family and Preventive Medicine (S Thirunavukkarasu PhD), Emory University, Atlanta, GA, USA; Department of Specialty Internal Medicine (Prof J A Al-Tawfiq MD), Johns Hopkins Aramco Healthcare, Dhahran, Saudi Arabia; Department of Medicine (Prof J A Al-Tawfiq MD), Indiana University School of Medicine, Indianapolis, IN, USA; Research Group in Health Economics (Prof N Alvis-Guzman PhD), Universidad de Cartagena (University of Cartagena), Cartagena, Colombia; Research Group in Hospital Management and Health Policies (Prof N Alvis-Guzman PhD), Universidad de la Costa (University of the Coast), Barranquilla, Colombia; Department of Medical Sciences (Prof Y M Al-Worafi PhD), Azal University for Human Development, Sana'a, Yemen; Department of Clinical Sciences (Prof Y M Al-Worafi PhD), University of Science and Technology of Fujairah, Fujairah, United Arab Emirates; Department of Pediatrics (Prof H Aly MD), Department of Internal Medicine (A Goyal MD), Lerner Research Institute (X Liu PhD), Cleveland Clinic, Cleveland, OH, USA; Department of Pediatric Cardiology (S Aly MD), Boston Children's Hospital, Boston, MA, USA; Department of Pediatrics (S Aly MD),

Department of Health Policy and Oral Epidemiology (Z S Natto DrPH), Department of Pulmonary and Critical Care (T O Olanipekun MD), Division of General Internal Medicine (Prof A Sheikh MD), Harvard University, Boston, MA, USA; Department of Biomedical Engineering (W A Al-Zyoud PhD), German Jordanian University, Amman, Jordan; Department of Public Health (A Amhare MSc), Salale University, Fitche, Ethiopia; School of Public Health (A Amhare MSc), Department of Pharmacy Administration and Clinical Pharmacy (K Hayat MS), Xi'an Jiaotong University, Xi'an, China; Department of Medicine (G A Amusa MD), University of Jos, Jos, Nigeria; Department of Internal Medicine (G A Amusa MD), Jos University Teaching Hospital, Jos, Nigeria; Department of Pharmacology (A Anil MD, S Singh MD), Department of Community Medicine and Family Medicine (Prof P Bhardwaj MD), School of Public Health (Prof P Bhardwaj MD), Department of Anatomy (R Gupta MD, H Krishna MD), Department of Physiology (Prof B Mehta MD), Department of Pharmacology and Research (A Saravanan MD), All India Institute of Medical Sciences, Jodhpur, India; All India Institute of Medical Sciences, Bhubaneswar, India (A Anil MD); Regenerative Medicine, Organ Procurement and Transplantation Multi-disciplinary Center (S Anvari MD), Inflammatory Lung Diseases Research Center (N Rahmanian PhD), Guilan University of Medical Sciences, Rasht, Iran; Department of Medical Laboratory Sciences (E E Anyabolo BMLS), University of Nigeria Nsukka, Enugu, Nigeria; Operations Department (E E Anyabolo BMLS), Breast Without Spot, Enugu, Nigeria; Health Management and Economics Research Center (J Arabloo PhD), School of Medicine (M Bastan MD), Department of Medical Laboratory Sciences (F Dorostkar PhD), Department of Ophthalmology (H Hasani MD), Endocrine Research Center (A Karimi Behnagh MD), Department of Echocardiography (A Karimi Behnagh MD), Institute of Immunology and Infectious Diseases (F Khamesipour PhD), Department of Anesthesiology (K Latifinaibin MD), Iran University of Medical Sciences, Tehran, Iran; College of Pharmacy (M Arafat PhD), AAU Health and Biomedical Research Center (Prof F El-Dahiyat PhD), Al Ain University, Abu Dhabi, United Arab Emirates; College of Art and Science (D Areda PhD), Ottawa University, Surprise, AZ, USA; School of Life Sciences (D Areda PhD), Arizona State University, Tempe, AZ, USA; College of Medicine and Health Sciences (B B Aregawi PhD), Department of Midwifery (M W Gebregergis MSc), Department of Medical Laboratory Sciences (H N Meles MSc, H L Weldetinsaa MSc), Adigrat University, Adigrat, Ethiopia; Department of Veterinary Pharmacology and Toxicology (A Aremu PhD), Department of Veterinary Public Health and Preventive Medicine (I A Odetokun PhD), University of Ilorin, Ilorin, Nigeria; Department of Immunology (S Athari PhD), Department of Critical Care and Emergency Nursing (N Hanifi PhD), Zanjan University of Medical Sciences, Zanjan, Iran; Northumbria HealthCare NHS Foundation Trust, Newcastle upon Tyne, UK (A Aujayeb MBBS); Department of Nursing (Z Aynalem MSc), Injibara University, Injibara, Ethiopia; Leeds Institute of Rheumatic and Musculoskeletal Medicine (S Azadnajafabad MD), University of Leeds, Leeds, UK; Montefiore-Einstein Cerebrovascular Research Lab (A Azzam MBBCh), Albert Einstein College of Medicine, Bronx, NY, USA; Faculty of Medicine (A Azzam MBBCh), October 6 University, 6th of October City, Egypt; Gomal Center of Biochemistry and Biotechnology (M Badar PhD), Gomal University, Dera Ismail Khan, Pakistan; Department of Biostatistics (P Bahrami Taghanaki MD), Orthodontics Department (M Ghorbani DDS), Endodontics Department (P Saeedi DDS), Applied Biomedical Research Center (Prof A Sahebkar PhD), Biotechnology Research Center (Prof A Sahebkar PhD), Department of Medical Informatics (S Tabatabaei PhD), Clinial Research Development Unit (S Tabatabaei PhD), Mashhad University of Medical Sciences, Mashhad, Iran; School of Medicine (S Bahramian MD), Heart Failure Research Center (S Najdaghi MD, D Narimani Davani MD), Neuroscience Research Center (S Najdaghi MD), Institute for Primordial Prevention of Non Communicable Disease (PPNCD) (S Roudashti MSc), Vice Chancellery of Health (S Roudashti MSc), Isfahan University of Medical Sciences, Isfahan, Iran;

International Medical School (A A Baig PhD), Management and Science University, Alam, Malaysia; Department of Pharmacology, Clinical Pharmacology and Toxicology (Prof M Bajcetic PhD), Faculty of Medicine (I M Ilic PhD, Prof M M Santric-Milicevic PhD), School of Public Health and Health Management (Prof M M Santric-Milicevic PhD), University of Belgrade, Belgrade, Serbia; Department for Clinical Pharmacology (Prof M Bajcetic PhD), University Children Hospital, Belgrade, Serbia; Division of Biological Sciences (S Balakrishnan PhD), Tamil Nadu State Council for Science and Technology, Chennai, India; Department of Hypertension (Prof M Banach PhD), Medical University of Lodz, Lodz, Poland; Polish Mothers' Memorial Hospital Research Institute, Lodz, Poland (Prof M Banach PhD); Miami Cancer Institute (M Bardhan MD), Baptist Health South Florida, Miami, FL, USA; Department of Medical Education (K Batra PhD), School of Public Health (R Batra MS), University of Nevada Las Vegas, Las Vegas, NV, USA; IT Department (R Batra MS), Coforge, Georgia, GA, USA; Endocrinology and Metabolism Research Institute (A Khalaji MD), Department of Epidemiology (S Nejadghaderi MD, S Rashedi MD), Non-Communicable Diseases Research Center (NCDRC), Tehran, Iran (A Behnoush BS); Division of Pulmonary, Critical Care, and Sleep (M Beiranvand PhD), College of Medicine (M J Diaz BS), University of Florida, Gainesville, FL, USA; Medical Laboratory Science (A G Belete MSc), Department of Medical Laboratory Science (M A Belete MSc), Department of Environmental Health (M Gebrehiwot DSc), Wollo University, Dessie, Ethiopia; Department of Biomedical Sciences (Prof A Beloukas PhD), National AIDS Reference Center of Southern Greece (Prof A Beloukas PhD), University of West Attica, Athens, Greece; School of Medicine (A Beran MD), Indiana University, Indianapolis, IN, USA; Department of Internal Medicine (A Bhargava MD), Wayne State University, Detroit, MI, USA; Department of General Medicine (A N Bhat MD), Department of Community Medicine (J Jose MD, N Joseph MD, R Motappa MD, R Thapar MD), Department of Forensic Medicine and Toxicology (Prof J Padubidri MD), Manipal Academy of Higher Education, Mangalore, India; Department of Pharmacy (Prof M A Bhuiyan PhD), University of Asia Pacific, Dhaka, Bangladesh; Faculty of Health Sciences (V R Bitra PhD), University of Botswana, Gaborone, Botswana; Department of Anesthesia and Critical Care Medicine (S Boppana MD), Department of Radiology (G G Z Zandieh MD), Department of International Health (H Zhang MS), Johns Hopkins University, Baltimore, MD, USA (E Jamshidi PharmD); Internal Medicine Department (H Borhany MD), Obesity Research Center (A Haj-Mirzaian MD), Student Research Committee (M Rahmanian MD), Social Determinants of Health Research Center (M Rashidi MD), Department of Health (M Safari PhD), Shahid Beheshti University of Medical Sciences, Tehran, Iran; Department of Medicine (Prof S Bouaoud DrPH), Faculty of Medicine (Prof A Ouyahia PhD), University Ferhat Abbas of Setif, Setif, Algeria; Department of Epidemiology and Preventive Medicine (Prof S Bouaoud DrPH), University Hospital Saadna Abdenour, Setif, Algeria; HCAI, Fungal, AMR, AMU, & Sepsis Division (C S Brown MD), United Kingdom Health Security Agency, London, UK; Department of Infection (C S Brown MD), Department of Primary Care and Public Health (Prof A Majeed MD, Prof S Rawaf MD), School of Public Health (Prof S Saxena MD), Imperial College London, London, UK; Department of Woman and Child Health and Public Health (D Buonsenso MD), Fondazione Policlinico Universitario A. Gemelli IRCCS (Agostino Gemelli University Polyclinic IRCCS), Rome, Italy; Global Health Research Institute (D Buonsenso MD), Università Cattolica del Sacro Cuore (Catholic University of Sacred Heart), Rome, Italy; Department of Internal Medicine (Prof L A Cámera MD), Hospital Italiano de Buenos Aires (Italian Hospital of Buenos Aires), Buenos Aires, Argentina; Board of Directors (Prof L A Cámera MD), Argentine Society of Medicine, Buenos Aires, Argentina; Colombian National Health Observatory (C A Castañeda-Orjuela PhD), Instituto Nacional de Salud (National Institute of Health), Bogota, Colombia; Epidemiology and Public Health Evaluation Group (C A Castañeda-Orjuela PhD), Department of Public Health (Prof F P De la Hoz PhD),

National University of Colombia, Bogota, Colombia; Department of Medical, Surgical, and Health Sciences (L Cegolon PhD), University of Trieste, Trieste, Italy; Public Health Unit (L Cegolon PhD), University Health Agency Giuliano-Isontina (ASUGI), Trieste, Italy; College of Public Health, Medical, and Veterinary Sciences (M Cenderadewi MPHTM), James Cook University, Townsville, QLD, Australia; Department of Public Health (M Cenderadewi MPHTM), University of Mataram, Mataram, Indonesia; State Disease Investigation Laboratory (S Chakraborty MVSc), Animal Resources Development Department, Agartala, India; Temerty Faculty of Medicine (V Chattu MD), University of Toronto, Toronto, ON, Canada; Department of Community Medicine (V Chattu MD), Datta Meghe Institute of Medical Sciences, Sawangi, India; Department of Paediatrics (E T W Cheng MBChB), The Chinese University of Hong Kong, Hong Kong, China; Division of Infectious Diseases (P R Ching MD), Virginia Commonwealth University, Richmond, VA, USA; Centre for Research Impact & Outcome (H Chopra PhD), Chitkara University, Rajpura, India; Department of Community Medicine (Prof S G Choudhari MD), Jawaharlal Nehru Medical College, Wardha, India; Department of Pulmonary Medicine (Prof D J Christopher MD), Christian Medical College and Hospital (CMC), Vellore, India; Center for Biomedicine and Community Health (D Chu PhD), International School, Vietnam National University Hanoi (VNUIS), Hanoi, Vietnam; Department of Paediatric Surgery (I S Chukwu BMedSc), Federal Medical Centre, Umuahia, Nigeria; Department of Respiratory Medicine and Allergology (Prof A Corlateanu PhD), Nicolae Testemitanu State University of Medicine and Pharmacy, Chisinau, Moldova; Department of Diagnostic and Therapeutic Technologies (Prof N Cruz-Martins PhD), Cooperativa de Ensino Superior Politécnico e Universitário (Polytechnic and University Higher Education Cooperative), Vila Nova de Famalicão, Portugal; Institute for Research and Innovation in Health (i3S) (Prof N Cruz-Martins PhD), University of Porto, Porto, Portugal; Department of Internal Medicine (S Dadana MD), Cheyenne Regional Medical Center, Cheyenne, WY, USA; Department of Global Public Health and Primary Care (O Dadras PhD), University of Bergen, Bergen, Norway; Department of Community Medicine (Prof T Dahiru MA), Ahmadu Bello University, Zaria, Nigeria; Division of Women and Child Health (J K Das MD), Aga Khan University, Karachi, Pakistan; Immunology Research Center (M Dashti MD, A Ghasemzadeh MD), School of Nursing and Midwifery (H Hassankhani PhD), Cardiovascular Research Center (M Rahimi MD), Tabriz University of Medical Sciences, Tabriz, Iran; Department of Gynecology and Obstetrics (M Dashtkoohi MD), Vali-E-Asr Reproductive Health Research Center, Family Health Research Institute, Tehran, Iran; Medical College (S Debopadhaya BS), Albany Medical College, Albany, NY, USA; USAID-JSI Digital Health Activity (B H Demessa MPH), Jimma University, Addis Ababa, Ethiopia; Department of Nursing (A B Demis MSc), Woldia University, Woldia, Ethiopia; School of Nursing (A B Demis MSc), Institute of Health Science (A I Mohamed MSc), Jimma University, Jimma, Ethiopia; Chettinad Hospital & Research Institute (Prof V Devanbu MD), Chettinad Academy of Research and Education, Chennai, India; JSS Medical College Department of Biochemistry (D Devegowda PhD), Jagadguru Sri Shivarathreeswara University, Mysuru, India; Division of Pathology (K Dhama PhD), ICAR-Indian Veterinary Research Institute, Bareilly, India; The Zena and Michael A. Wiener Cardiovascular Institute (V R Dhulipala MD), Department of Cardiology (M Vinayak MD), Icahn School of Medicine at Mount Sinai, New York, NY, USA; Faculty of Science (Prof D Diaz PhD), National Autonomous University of Mexico, Mexico City, Mexico; Department of Medicine (T C Do MD), Pham Ngoc Thach University of Medicine, Ho Chi Minh City, Vietnam; Department of Medicine (T H Do MD), Can Tho University of Medicine and Pharmacy, Can Tho, Vietnam; Department of Biostatistics (M Dodangeh Mcom), Independent Consultant, Tehran, Iran; Department of Medicine (A C Dsouza MBBS, A Sathyanarayan MD), Bangalore Medical College and Research Institute, Bangalore, India; Manipal Academy of Higher Education (H L Dsouza MD), Kasturba Medical College Mangalore (R

Holla MD, M Rao MD), Department of Physiotherapy (Prof V K PhD), Department of Nephrology (I Rao DM), Manipal Academy of Higher Education, Manipal, India; Department of Forensic Medicine and Toxicology (H L Dsouza MD), Kasturba Medical College Mangalore, Mangalore, India; Faculty of Science and Humanities (S Duraisamy PhD), Sri Ramaswamy Memorial Institute of Science and Technology, Kattankulathur, India; Department of Infection and Tropical Medicine (O C Durojaiye MPH), Department of Psychology (A Yadollahpour PhD), University of Sheffield, Sheffield, UK; Department of Conservative Dentistry with Endodontics (A M Dziedzic DSc), Medical University of Silesia, Katowice, Poland; Higher School of Technology (Prof A Ed-Dra PhD), Sultan Moulay Slimane University, Beni Mellal, Morocco; Faculty of Science and Health (M Ekholuenetale PhD), University of Portsmouth, Hampshire, UK; Department of Microbiology (T C Ekundayo PhD), Department of Biosciences and Biotechnology (A J Udoakang PhD), University of Medical Sciences, Ondo, Ondo, Nigeria; Biomedical Informatics and Medical Statistics Department (I El Sayed PhD), Department of Pathology (Prof I M Talaat PhD), Alexandria University, Alexandria, Egypt; Clinical Pharmacy Program (Prof F El-Dahiyat PhD), Al Ain University, Al Ain, United Arab Emirates; Faculty of Medicine (M Elhadi MD), University of Tripoli, Tripoli, Libya; Houston Methodist Hospital, Houston, TX, USA (M Elhadi MD); Department of Clinical Pathology (Prof M Elshaer PhD), Department of Cardiology (Prof M M Ramadan PhD), Faculty of Pharmacy (Prof M A Saleh PhD), Mansoura University, Mansoura, Egypt; Department of Bacteriology and Virology (M Eslami PhD), Semnan University of Medical Sciences, Semnan, Iran; Cancer Research Center (M Eslami PhD), Semnan University of Medical Scinces, Semnan, Iran; Department of Ophthalmology (U A Eze MD), Federal Medical Centre, Asaba, Nigeria; Postgraduate School (U A Eze MD), Centre for Medical Informatics (Prof A Sheikh MD), Usher Institute (Prof C R Simpson PhD), University of Edinburgh, Edinburgh, UK; Department of Epidemiology and Medical Statistics (A F Fagbamigbe PhD), Department of Health Promotion and Education (S Ibitoye PhD), College of Medicine (A P Okekunle PhD), Faculty of Public Health (I I Olufadewa MHS), University of Ibadan, Ibadan, Nigeria; Research Centre for Healthcare and Community (A F Fagbamigbe PhD), Faculty of Health and Life Sciences (O P Kurmi PhD), Coventry University, Coventry, UK; Student Research Committee (A Faramarzi MD), Department of Otolaryngology (A Faramarzi MD), Department of Occupational Health and Safety Engineering (R Pourbabaki PhD), Shiraz University of Medical Sciences, Shiraz, Iran; Department of Veterinary Tropical Diseases (Prof F O Fasina PhD), University of Pretoria, Pretoria, South Africa; Animal Production and Health Division (EMPRES) (Prof F O Fasina PhD), Food and Agriculture Organization of the United Nations, Rome, Italy; Department of Social Sciences (Prof N Ferreira PhD), University of Nicosia, Nicosia, Cyprus; Institute of Public Health (F Fischer PhD), Charité Universitätsmedizin Berlin (Charité Medical University Berlin), Berlin, Germany; Department of Pharmacology (I Fitriana PhD), Gadjah Mada University, Yogyakarta, Indonesia; Institute of Applied Health Sciences (S Gaihre PhD), University of Aberdeen, Aberdeen, UK; Department of Oral Biology and Experimental Dental Research (M Gajdács PhD), University of Szeged, Szeged, Hungary; Faculty of Paramedicine (N Galehdar PhD), Lorestan University of Medical Sciences, Khorramabad, Iran; Department of General Medicine (M Ganiyani MD), Grant Medical College & Sir J.J. Group of Hospitals, Mumbai, India; Department of Medicine (M Ganiyani MD), Miami Cancer Institute, Miami, FL, USA; Department of Reproductive and Family Health (T G Gebremeskel PhD), Axum College of Health Science, Axum, Ethiopia; College of Medicine and Public Health (T G Gebremeskel PhD, G R Naik PhD), Department of Nursing and Health Sciences (S Shorofi PhD), Flinders University, Adelaide, SA, Australia; Department of Public Health (G K Getahun MPH), Menelik II Medical and Health Science College, Addis Ababa, Ethiopia; Department of Medical Laboratory Science (M Getie MSc), Addis Ababa University, Addis Ababa, Ethiopia; Infectious Disease

Research Center (Prof K Ghadiri MD), Pediatric Department (Prof K Ghadiri MD), Universal Scientific Education and Research Network (USERN) (P Goleij MSc), Kermanshah University of Medical Sciences, Kermanshah, Iran; Department of Health Systems and Policy Research (M Golechha PhD), Indian Institute of Public Health, Gandhinagar, India; Department of Genetics (P Goleij MSc), Sana Institute of Higher Education, Sari, Iran; Oncological Network, Prevention and Research Institute (G Gorini MD), Institute for Cancer Research, Prevention and Clinical Network, Florence, Italy; Department of Epidemiology and Biostatistics (S Guan MD, C Zhai MD), Anhui Medical University, Hefei, China; Post Graduate School of Public Health (G Guarducci MD), University of Siena, Siena, Italy; Department of Clinical Pharmacy (M D Gudeta MSc), Haramaya University, Harar, Ethiopia; Department of Toxicology (S Gupta MSc), Shriram Institute for Industrial Research, Delhi, India; School of Medicine (V Gupta PhD), Deakin University, Geelong, VIC, Australia; Faculty of Medicine Health and Human Sciences (Prof V K Gupta PhD), Australian Institute of Health Innovation (P Peprah MSc), Macquarie University, Sydney, NSW, Australia; Department of Clinical Pharmacology and Medicine (Prof N R Hadi PhD), University of Kufa, Najaf, Iraq; Department of Radiology (A Haj-Mirzaian MD, X Liu PhD), Division of Cardiology (D H Nguyen BS), Massachusetts General Hospital, Boston, MA, USA (M Kim MD); School of Health and Environmental Studies (Prof S Hamidi DrPH), Hamdan Bin Mohammed Smart University, Dubai, United Arab Emirates; Department of Medical and Technical Information Technology (A Hammoud PhD), Bauman Moscow State Technical University, Moscow, Russia; Department of Health and Education (F Hanna PhD), Torrens University Australia, Melbourne, VIC, Australia; The Warren Alpert Medical School (Z A Haq BA), Brown University, Providence, RI, USA; Department of Population Sciences (M Haque PhD), University of Dhaka, Dhaka, Bangladesh; Department of Biomedical Engineering and Public Health (S Hasan PhD), World University of Bangladesh, Dhaka, Bangladesh; Department of Pharmacy (Prof M S Hasnain PhD), Palamau Institute of Pharmacy, Daltonganj, India; Independent Consultant, Tabriz, Iran (H Hassankhani PhD); Department of Diagnostic and Interventional Radiology and Neuroradiology (Prof J Haubold MD, Prof B M Schaarschmidt MD), Institute of Artificial Intelligence in Medicine (Prof J Haubold MD), University Hospital Essen, Essen, Germany; Institute of Pharmaceutical Sciences (K Hayat MS), University of Veterinary and Animal Sciences, Lahore, Pakistan; Department of Microbiology (K Hezam PhD), Taiz University, Taiz, Yemen; School of Medicine (K Hezam PhD), Nankai University, Tianjin, China; School of Social Sciences (P Hoogar PhD), The Apollo University, Chittoor, India; Department of Pulmonology (N Horita PhD), Yokohama City University, Yokohama, Japan; National Human Genome Research Institute (NHGRI) (N Horita PhD), National Institutes of Health, Bethesda, MD, USA; Department of Internal Medicine (M Hostiuc PhD), Carol Davila University of Medicine and Pharmacy, Bucharest, Romania; International Master Program for Translational Science (H Huynh BS), International Ph.D. Program in Medicine (L Minh MD), Research Center for Artificial Intelligence in Medicine (L Minh MD), Taipei Medical University, Taipei, Taiwan; West Africa RCC (O S Ilesanmi PhD), Africa Centre for Disease Control and Prevention, Abuja, Nigeria; Department of Community Medicine (O S Ilesanmi PhD), Department of Oral and Maxillofacial Surgery (A A Salami BDS), University College Hospital, Ibadan, Ibadan, Nigeria; Faculty of Medical Sciences (Prof M D Ilic PhD), University of Kragujevac, Kragujevac, Serbia; Department of Clinical Pharmacy (M Imam PhD), Department of Electrical Engineering (I Malik PhD), Prince Sattam bin Abdulaziz University, Al Kharj, Saudi Arabia; Department of Biotechnology (M A Isa PhD), Sharda University, Greater Noida, India; School of Pharmacy (M Islam PhD), BRAC University, Dhaka, Bangladesh; Institute for Physical Activity and Nutrition (S Islam PhD), Deakin University, Burwood, VIC, Australia; Sydney Medical School (S Islam PhD), Save Sight Institute (H Kandel PhD), Department of Public Health (M Khan PhD), School of Chemical & Biomolecular Engineering (E A Noman

PhD), University of Sydney, Sydney, NSW, Australia; Department of Clinical Pharmacy & Pharmacy Practice (Prof N Ismail PhD), Asian Institute of Medicine, Science and Technology, Bedong, Malaysia; Malaysian Academy of Pharmacy, Puchong, Malaysia (Prof N Ismail PhD); Department of Health Services Research (M Iwagami PhD), University of Tsukuba, Tsukuba, Japan; Department of Non-Communicable Disease Epidemiology (M Iwagami PhD), London School of Hygiene & Tropical Medicine, London, UK; Department of Community Medicine and Family Medicine (V J MD), All India Institute of Medical Sciences, Gorakhpur, India; Department of Immunology (Prof A Jafarzadeh PhD), HIV/STI Surveillance Research Center (S Nejadghaderi MD), Kerman University of Medical Sciences, Kerman, Iran; Department of Immunology (Prof A Jafarzadeh PhD), Rafsanjan University of Medical Sciences, Rafsanjan, Iran; Department of Nephrology (K Jaggi MD), San Mateo Medical Center, San Mateo, CA, USA; Department of Nephrology (K Jaggi MD), Mills Peninsula Medical Center, Burlingame, CA, USA; Department of Health and Safety (A A Jairoun PhD), Dubai Municipality, Dubai, United Arab Emirates; The World Academy of Sciences UNESCO, Trieste, Italy (Prof M Jakovljevic PhD); Shaanxi University of Technology, Hanzhong, China (Prof M Jakovljevic PhD); Department of Biochemistry (Prof S Jayaram MD), Government Medical College, Mysuru, India; Department of Internal Medicine (B M Jeswani MBBS), GCS Medical College, Hospital & Research Centre, Ahmedabad, India; Department of Community Medicine (R P Jha MSc), Dr. Baba Saheb Ambedkar Medical College & Hospital, Delhi, India; Department of Community Medicine (R P Jha MSc), Banaras Hindu University, Varanasi, India; Department of Economics (C E Joshua BSc), National Open University, Benin City, Nigeria; Department of Family Medicine and Public Health (J J Jozwiak PhD), University of Opole, Opole, Poland; School of Public Health (Z Kabir PhD), University College Cork, Cork, Ireland; Sydney Eye Hospital (H Kandel PhD), South Eastern Sydney Local Health District, Sydney, NSW, Australia; Faculty of Dentistry (K K Kanmodi MPH, A A Salami BDS), University of Puthisastra, Phnom Penh, Cambodia; Office of the Executive Director (K K Kanmodi MPH), Cephas Health Research Initiative Inc, Ibadan, Nigeria; Department of Respiratory Medicine (Prof S Kant MD), King George's Medical University, Lucknow, India; The Hansjörg Wyss Department of Plastic and Reconstructive Surgery (R S Kantar MD), Nab'a Al-Hayat Foundation for Medical Sciences and Health Care, New York, NY, USA; Cleft Lip and Palate Surgery Division (R S Kantar MD), Global Smile Foundation, Norwood, MA, USA; School of Health Professions and Human Services (I M Karaye MD), Hofstra University, Hempstead, NY, USA; Department of Anesthesiology (I M Karaye MD), Montefiore Medical Center, Bronx, NY, USA; Department of ENT (N Kaur MS), Dr. B. R. Ambedkar State Institute of Medical Sciences (AIMS), Mohali, India; Amity Institute of Forensic Sciences (H Khajuria PhD, B P Nayak PhD), Amity Institute of Public Health (M Shannawaz PhD), Amity University, Noida, India; Halal Research Centre of IRI, Iran Food and Drug Administration (F Khamesipour PhD), Ministry of Health and Medical Education, Tehran, Iran; Population Science Department (M Khan PhD), Jatiya Kabi Kazi Nazrul Islam University, Mymensingh, Bangladesh; Department of Epidemiology (M Khan MD), Department of Health Education and Promotion (M Shanawaz MD), Jazan University, Jazan, Saudi Arabia; Broad Institute of MIT and Harvard, Cambridge, MA, USA (M Kim MD); Millennium Prevention, Inc., Westwood, MA, USA (R W Kimokoti MD); Global Healthcare Consulting, New Delhi, India (S Kochhar MD); Department of Epidemiology and Evidence-Based Medicine (V A Korshunov PhD, R V Polibin PhD), I.M. Sechenov First Moscow State Medical University, Moscow, Russia; Independent Consultant, Jakarta, Indonesia (S Kosen MD); Department of Anthropology (Prof K Krishan PhD), Panjab University, Chandigarh, India; Department of Anesthesiology (V Krishnamoorthy MD), Duke University, Durham, NC, USA; Department of Demography (Prof B Kuate Defo PhD), Department of Social and Preventive Medicine (Prof B Kuate Defo PhD), University of Montreal, Montreal, QC, Canada; Department of Mathematics (M Kuddus PhD),

Department of Population Science and Human Resource Development (Prof M Rahman DrPH), University of Rajshahi, Rajshahi, Bangladesh; Department of Biochemistry (Prof M Kuddus PhD), Department of Pharmaceutics (S Rizvi PhD), University of Hail, Hail, Saudi Arabia; Department of Pediatrics (I Kuitunen PhD), Kuopio University Hospital, Kuopio, Finland; Institute of Clinical Medicine (I Kuitunen PhD), University of Eastern Finland, Kuopio, Finland; Atchabarov Scientific-Research Institute of Fundamental and Applied Medicine (M Kulimbet MSc), Kazakh National Medical University, Almaty, Kazakhstan; Center of Medicine and Public Health (M Kulimbet MSc), Asfendiyarov Kazakh National Medical University, Almaty, Kazakhstan; Community Medicine (D Kumar MD), Rajendra Institute of Medical Sciences, Ranchi, India; Department of Medicine (O P Kurmi PhD), McMaster University, Hamilton, ON, Canada; Department of General Surgery (L Kutikuppala BMedSc), Dr NTR University of Health Sciences, Vijayawada, India; Integrated Department of Epidemiology, Health Policy, Preventive Medicine & Pediatrics (Prof C Lahariya MD), Foundation for People-centric Health Systems, New Delhi, India; SD Gupta School of Public Health (Prof C Lahariya MD), Indian Institute of Health Management Research University, Jaipur, India; Indian Council of Medical Research, New Delhi, India (D K Lal MD); Department of Otorhinolaryngology (S Lasrado MS), Father Muller Medical College, Mangalore, India; Faculty of Medicine (H Le MD, N Le MD), Department of General Medicine (V T Nguyen MD), University of Medicine and Pharmacy at Ho Chi Minh City, Ho Chi Minh City, Vietnam (T T Le MD, T D T Le MD); Department of Cardiovascular Research (H Le MD, N Le MD), Methodist Hospital, Merrillville, IN, USA; Independent Consultant, Ho Chi Minh City, Vietnam (T D T Le MD); Department of Precision Medicine (Prof S Lee MD), Sungkyunkwan University, Suwon-si, South Korea; Department of Health Promotion and Health Education (M Li PhD), National Taiwan Normal University, Taipei, Taiwan; Department of Thoracic Surgery (P Li PhD), The First Affiliated Hospital of Xi'an Jiaotong University, Xi'an, China; Center for Evidence-Based Medicine and Clinical Research (R Liu PhD), School of Public Health and Management (Y Yu MS), Hubei University of Medicine, Shiyan, China; Institute for Health and Environment (W Liu PhD), Chongqing University of Science and Technology, Chongqing, China; Department of Quantitative Health Science (X Liu PhD), Case Western Reserve University, Cleveland, OH, USA; Department of Health Economics (L Lorenzovici MSc), Syreon Research Romania, Targu Mures, Romania; Department of Doctoral Studies (L Lorenzovici MSc), George Emil Palade University of Medicine, Pharmacy, Science, and Technology of Targu Mures, Targu Mures, Romania; Center for Evidence-Based and Translational Medicine (L Luo MPH), School of Public Health (Prof Z Zhang PhD), Wuhan University, Wuhan, China; Rama Medical College Hospital and Research Centre, Uttar Pradesh, India (K Malhotra MBBS); Institute of Applied Health Research (K Malhotra MBBS), University of Birmingham, Birmingham, UK; School of Public Health (K Malhotra MBBS), University of Adelaide, Adelaide, SA, Australia; Department of Medical Laboratory Sciences (A Manilal PhD), Department of Public Health (T Mekene Meto MPH), Arba Minch University, Arba Minch, Ethiopia; Department of Nursing (M M Mekonnen MSc), Salale University, Fiche, Ethiopia; College of Medicine (Prof Z A Memish MD), Alfaisal University, Riyadh, Saudi Arabia; Research & Innovation Center (Prof Z A Memish MD), Ministry of Health, Riyadh, Saudi Arabia; Department of Medical Oncology and Hematology (M A Mendez-Lopez PhD), Kantonsspital St. Gallen, St. Gallen, Switzerland; Department of Physiology (Prof S A Meo PhD), Pediatric Intensive Care Unit (Prof M Temsah MD), King Saud University, Riyadh, Saudi Arabia; University Centre Varazdin (T Mestrovic PhD), University North, Varazdin, Croatia; Department of Paediatrics (Prof S Mettananda DPhil), University of Kelaniya, Ragama, Sri Lanka; University Paediatrics Unit (Prof S Mettananda DPhil), Colombo North Teaching Hospital, Ragama, Sri Lanka; Internal Medicine Programme (Prof E M Mirrakhimov PhD), Kyrgyz State Medical Academy, Bishkek, Kyrgyzstan; Department of Atherosclerosis and Coronary Heart Disease

(Prof E M Mirrakhimov PhD), National Center of Cardiology and Internal Disease, Bishkek, Kyrgyzstan; Department of Pharmacology (A K Misra MD), All India Institute of Medical Sciences, Mangalagiri, India; College of Health Science (A I Mohamed MSc), University of Hargeisa, Hargeisa, Somalia; Molecular Biology Unit (N S Mohamed MSc), Bio-Statistical and Molecular Biology Department (N S Mohamed MSc), Sirius Training and Research Centre, Khartoum, Sudan; Clinical Epidemiology and Public Health Research Unit (L Monasta DSc, L Ronfani PhD), Burlo Garofolo Institute for Maternal and Child Health, Trieste, Italy; AI & Cyber Futures Institute (M Moni PhD), Charles Sturt University, Bathurst, NSW, Australia; Faculty of Medicine (B Sartorius PhD), The University of Queensland, Brisbane, QLD, Australia (M Moni PhD); Faculty of Medicine (A Moodi Ghalibaf MD), Birjand University of Medical Sciences, Birjand, Iran; Centre for Neonatal and Paediatric Infection (C E Moore PhD), St. George's University of London, London, UK; International Laboratory for Air Quality and Health (Prof L Morawska PhD), Queensland University of Technology, Brisbane, QLD, Australia; Department of Pediatrics (Prof G Mustafa MD), Department of Pharmacology (A R Pathan PhD, M Tabish MPharm), Shaqra University, Shaqra, Saudi Arabia; Department of Pediatrics & Pediatric Pulmonology (Prof G Mustafa MD), Institute of Mother & Child Care, Multan, Pakistan; Department of Computer Science (P Naghavi MS), University of Illinois Urbana-Champaign, Urbana, IL, USA; Department of Engineering (G R Naik PhD), Western Sydney University, Sydney, NSW, Australia; Faculty of Pharmacy (F Nainu PhD), Hasanuddin University, Makassar, Indonesia; Department of Medical Laboratory Analysis (H H Najmuldeen PhD), Cihan University Sulaymaniya, Sulaymaniyah, Iraq; University Institute of Public Health (S Nargus PhD), Institute of Molecular Biology and Biotechnology (S Shahid PhD), Research Centre for Health Sciences (RCHS) (S Shahid PhD), The University of Lahore, Lahore, Pakistan; Emergency Department (M Naser MD), Zayed Military Hospital, Abu Dhabi, United Arab Emirates; Department of Dental Public Health (Z S Natto DrPH), King Abdulaziz University, Jeddah, Saudi Arabia; Department of Medical Engineering (D H Nguyen BS), University of South Florida, Tampa, FL, USA; Faculty of Medicine (H T H Nguyen MD), Institue for Research and Training in Medicine, Biology and Pharmacy (H T H Nguyen MD), Duy Tan University, Da Nang, Vietnam; Department of General Surgery (T K Nikolouzakis PhD), University Hospital of Heraklion, Heraklion, Greece; Laboratory of Toxicology (T K Nikolouzakis PhD), University of Crete, Heraklion, Greece; Faculty of Applied Sciences and Technology (E A Noman PhD), Universiti Tun Hussein Onn Malaysia, Johor, Malaysia; Department of Paediatrics (C A Nri-Ezedi PhD), Nnamdi Azikiwe University, Awka, Nigeria; Unit of Microbiology and Public Health (V Nuñez-Samudio PhD), Instituto de Ciencias Medicas, Las Tablas, Panama; Department of Public Health (V Nuñez-Samudio PhD), Ministry of Health, Herrera, Panama; Department of Pediatrics (V E Nwatah MD), National Hospital Abuja, Abuja, Nigeria; Department of International Public Health (V E Nwatah MD), University of Liverpool, Liverpool, UK; Department of Food and Nutrition (A P Okekunle PhD), Seoul National University, Seoul, South Korea; School of Pharmacy (O C Okonji MSc), University of the Western Cape, Cape Town, South Africa; Department of Medical Physiology (P G Okwute MSc), Babcock University, Ilisan-Remo, Nigeria; Department of Medical Physiology (P G Okwute MSc), University of Lagos, Lagos, Nigeria; Slum and Rural Health Initiative Research Academy (I I Olufadewa MHS), Slum and Rural Health Initiative, Ibadan, Nigeria; Centre for Healthy Start Initiative, Lagos, Nigeria (B O Olusanya PhD); Surgery Department (G L Omer MD), Sulaimani University, Sulaimani, Iraq; ENT Department (G L Omer MD), Tor Vergata University of Rome, Rome, Italy; Department of Biomedical Sciences (K I Onyedibe PhD), Mercer University School of Medicine, Macon, GA, USA; Department of Pharmacotherapy and Pharmaceutical Care (M Ordak PhD), Department of Biochemistry and Pharmacogenomics (M Zielińska MPharm), Medical University of Warsaw, Warsaw, Poland; Sickle Cell Unit (Prof V N Orish PhD), Ho Teaching

Hospital, Ho, Ghana; One Health Global Research Group (Prof E Ortiz-Prado PhD), Universidad de las Americas (University of the Americas), Quito, Ecuador; Laboratory of Public Health Indicators Analysis and Health Digitalization (N Otstavnov BA), Moscow Institute of Physics and Technology, Dolgoprudny, Russia; Division of Infectious Diseases (Prof A Ouyahia PhD), University Hospital of Setif, Setif, Algeria; Department of Respiratory Medicine (Prof M P P A DNB), Jagadguru Sri Shivarathreeswara University, Mysore, India; Research Department (A Pandey MPH), Nepal Health Research Council, Kathmandu, Nepal; Research Department (A Pandey MPH), Public Health Research Society Nepal, Kathmandu, Nepal; Department of Emergency Medicine (Prof I Pantazopoulos PhD), University of Thessaly, Larissa, Greece; Department of Emergency Medicine (Prof I Pantazopoulos PhD), University of Bern, Bern, Switzerland; Vision and Eye Research Institute (Prof S Pardhan PhD), Anglia Ruskin University, Cambridge, UK; Department of Community Medicine (P P Parija MD), All India Institute of Medical Sciences, Jammu, India; Department of Epidemiology and Community Health (R R Parikh MD), University of Minnesota, Minneapolis, MN, USA; Department of Biomedical Data Science (S Park MD), Department of Radiology (S Ramasamy MD), Stanford University, Stanford, CA, USA; Center for Pharmacoepidemiology and Treatment Science (A Parthasarathi MD), Rutgers University, New Brunswick, NJ, USA; Research Center (A Parthasarathi MD), Allergy Asthma and Chest Center, Mysore, India; Research Consultancy (A R Pathan PhD), Author Gate Publications, Malegaon, India; College of Dental Medicine (Prof S Patil PhD), Roseman University of Health Sciences, South Jordan, UT, USA; Centre of Molecular Medicine and Diagnostics (COMManD) (Prof S Patil PhD), Saveetha Dental College and Hospitals (M Tovani-Palone PhD), Saveetha University, Chennai, India; Commission of Academic Accreditation (Prof D Perumal PhD), Ministry of Education, Abu Dhabi, United Arab Emirates; Department of Statistics and Econometrics (I Petcu PhD), Bucharest University of Economic Studies, Bucharest, Romania; Department of Internal Medicine (H Pham MD), University of Arizona, Tucson, AZ, USA; Department of Cardiovascular Medicine (H Pham MD), Mayo Clinic, Rochester, MN, USA; Department of Internal Medicine (H Pham MD), Weiss Memorial Hospital, Chicago, IL, USA; School of Pharmacy (A K Philip PhD), University of Nizwa, Nizwa, Oman; International Center of Medical Sciences Research, Islamabad, Pakistan (Z Z Piracha PhD); Clinical Academic Department of Pediatrics (Prof D Poddighe PhD), University Medical Center (UMC), Astana, Kazakhstan; University Medical Center Groningen (Prof M J Postma PhD), University of Groningen, Groningen, Netherlands; Department of Biostatistics, Epidemiology, and Informatics (J Puvvula PhD), Department of Otorhinolaryngology (P S Ramesh PhD), University of Pennsylvania, Philadelphia, PA, USA; Department of Cardiology (G Qian MS), Guiqian International General Hospital, Guiyang, China; Department of Medical Laboratory Technologies (Prof F Rahim PhD), Al-Noor Center of Research and Innovation (Prof F Rahim PhD), Alnoor University, Mousl, Iraq; National Institute of Infectious Diseases (M Rahman PhD), Center for Surveillance, Immunization, and Epidemiologic Research, Tokyo, Japan; Center for Evidence-Based Medicine and Clinical Research, Dhaka, Bangladesh (M Rahman PhD); Institute of Health and Wellbeing (Prof M Rahman PhD), Federation University Australia, Berwick, VIC, Australia; School of Nursing and Midwifery (Prof M Rahman PhD), La Trobe University, Melbourne, VIC, Australia; Department of Public Health (V Rahmanian PhD), Torbat Jam Faculty of Medical Sciences, Torbat Jam, Iran; CEReSS-Health Service Research and Quality of Life Center (Prof M Rahmati PhD), Aix-Marseille University, Marseille, France; Centre for Chronic Disease Control, New Delhi, India (P Rajput PhD); Department of Oral Pathology, Microbiology and Forensic Odontology (S Rao MDS), Sharavathi Dental College and Hospital, Shimogga, India; Thrombosis Research Group (S Rashedi MD), Brigham and Women's Hospital, Harvard Medical School, Boston, MA, USA; Department of Family Medicine (Prof D Rathish MPH), Department of Community Medicine (N D Wickramasinghe MD), Rajarata University of Sri

Lanka, Anuradhapura, Sri Lanka; Section of Pulmonary and Critical Care Medicine (N Ravikumar MD), University of Chicago, Chicago, IL, USA; Academic Public Health England (Prof S Rawaf MD), Public Health England, London, UK; Department of Biological Sciences (Prof E M M Redwan PhD), King Abdulaziz University, Jeddah, Egypt; Department of Protein Research (Prof E M M Redwan PhD), Research and Academic Institution, Alexandria, Egypt; Unisabana Center for Translational Science (L F Reyes PhD), Universidad de La Sabana (Savannah University), Chia, Colombia; Critical Care Department (L F Reyes PhD), Clinica Universidad De La Sabana (Savannah University Clinic), Chia, Colombia; Network of Immunity in Infection, Malignancy and Autoimmunity (NIIMA) (Prof N Rezaei PhD), Universal Scientific Education and Research Network (USERN), Tehran, Iran; Department of Infectious Diseases (O Rezahosseini MD), University of Copenhagen, Copenhagen, Denmark; Department of Pharmacology and Toxicology (Prof J A B Rodriguez PhD), University of Antioquia, Medellin, Colombia; Warwick Medical School (Prof J A B Rodriguez PhD), University of Warwick, Coventry, UK; Department of Labour (P Roy PhD), Directorate of Factories, Government of West Bengal, Kolkata, India; Advanced Campus Governador Valadares (Prof G d Ruela MSc), Juiz de For a Federal University, Governador Valadares, Brazil; Nursing Department (Prof G d Ruela MSc), Universidade Presidente Antônio Carlos (President Antônio Carlos University), Governador Valadares, Brazil; Department of Pharmaceutical Chemistry (Prof M Saeb PhD), International Medical University, Gdańsk, Poland; Clinical and Biomedical Research Center (Prof U Saeed PhD), Foundation University Islamabad, Islamabad, Pakistan; International Center of Medical Sciences Research (ICMSR), Islamabad, Pakistan (Prof U Saeed PhD); Department of Preventive & Social Medicine (M Sahu MD), All India Institute of Hygiene & Public Health, Kolkata, India; LMU-Munich, Munich, Germany (J W Sakshaug PhD); Institute for Employment Research, Nuremberg, Germany (J W Sakshaug PhD); Department of Biosciences (N Salam PhD), Jamia Millia Islamia, New Delhi, India; Institute of Epidemiology and Preventive Medicine (Y L Samodra PhD), National Taiwan University, Taipei, Taiwan; Benang Merah Research Center, Minahasa Utara, Indonesia (Y L Samodra PhD); Department of Pediatrics (Prof R K Sanjeev MD), Sree Balaji Medical College and Hospital, Chennai, India; Indira Gandhi Medical College and Research Institute, Puducherry, India (A Saravanan MD); Nuffield Department of Medicine (B Sartorius PhD), University of Oxford, Oxford, UK; Department of Medical Informatics (J Saulam MSc), Kagawa University, Miki-cho, Japan; Food Processing and Nutrition (J Saulam MSc), Karnataka State Akkamahadevi Women's University, Vijayapura, India; Department of Preventive and Social Medicine (G Saya MD), Jawaharlal Institute of Postgraduate Medical Education and Research, Puducherry, India; Department of Microbiology (M Sedighi PhD), Kurdistan University of Medical Sciences, Sanandaj, Iran; Department of Clinical Pharmacy (A K Sendekie MSc), University of Gondar, Gondar, Ethiopia; Emergency Department (S Senthilkumaran PhD), Manian Medical Centre, Erode, India; Department of Medicine and Surgery (Y Sethi MBBS), Government Doon Medical College, Dehradun, India; Independent Consultant, Karachi, Pakistan (M A Shaikh MD); Department of Pathology and Laboratory Medicine (S Sham MD), Northwell Health, New York, NY, USA; Department of Pathobiology (M Shamshirgaran PhD), Shahid Bahonar University of Kerman, Kerman, Iran; Department of Biomedical Sciences (J Sharifi-Rad PhD), Korea University, Seoul, South Korea; Yenepoya Research Center (R P Shastry PhD), Yenepoya University, Mangalore, India; National Institute of Infectious Diseases, Tokyo, Japan (M Shigematsu PhD); Finnish Institute of Occupational Health, Helsinki, Finland (R Shiri PhD); Department of Veterinary Public Health and Preventive Medicine (A Shittu MSc), Usmanu Danfodiyo University, Sokoto, Sokoto, Nigeria; Oulu Business School (I Shiue PhD), Martti Ahtisaari Institute (I Shiue PhD), University of Oulu, Oulu, Finland; Department of Medical-Surgical Nursing (S Shorofi PhD), Mazandaran University of Medical Sciences, Sari, Iran; Department of Medical

Microbiology and Infectious Diseases (E E Siddig MD), Erasmus University, Rotterdam, Netherlands; School of Health (Prof C R Simpson PhD), Victoria University of Wellington, Wellington, New Zealand; School of Medicine (Prof J A Singh MD), Henry JN Taub Department of Emergency Medicine (Prof L Szarpak PhD), Baylor College of Medicine, Houston, TX, USA; Medicine Service (Prof J A Singh MD), US Department of Veterans Affairs (VA), Houston, TX, USA; Department of Radiodiagnosis (P Singh MD), All India Institute of Medical Sciences, Bathinda, India; Department of Internal Medicine (R Sinto MD), University of Indonesia, Jakarta Pusat, Indonesia; Department of Internal Medicine (R Sinto MD), Dr. Cipto Mangunkusumo National Hospital, Jakarta Pusat, Indonesia; Department of Systemic Pathology (R Solanki MD), Touro College of Osteopathic Medicine, Middletown, NY, USA; Department of Pathology (R Solanki MD), American University of the Caribbean School of Medicine, Cupecoy, Saint Martin; School of Life Sciences (M Suleman PhD), Xiamen University, Xiamen, China; National Institute of Epidemiology (R Suliankatchi Abdulkader MD), Indian Council of Medical Research, Chennai, India; Department of Analytical and Applied Economics (C Swain MPhil), Utkal University, Bhubaneswar, India; Department of Clinical Research and Development (Prof L Szarpak PhD), LUXMED Group, Warsaw, Poland; Duhok Research Centre (Z M Taha BMedSc), University of Duhok, Duhok, Iraq; Department of Environmental, Agricultural and Occupational Health (J Taiba MPH), University of Nebraska Medical Center, Omaha, NE, USA; Sri Ramachandra Medical College and Research Institute, Chennai, India (J Taiba MPH); Department of Epidemiology (J L Tamuzi MSc), Stellenbosch University, Cape Town, South Africa; Department of Medicine (J L Tamuzi MSc), Northlands Medical Group, Omuthiya, Namibia; Department of Public Health (Y M Tefera MPH), Dire Dawa University, Dire Dawa, Ethiopia; Outpatient Department (D R Terefa MSc), Wollega University, Bedele town, Ethiopia; Department of Public Health (D R Terefa MSc), Wollega University, Nekemte, Ethiopia; School of Humanities and Social Sciences (R Thakur PhD), Indian Institute of Technology Mandi, Mandi, India; Faculty of Biomedical Engineering (A Tichopad PhD), Czech Technical University, Prague, Czech Republic; Faculty of Public Health (J H V Ticoalu MPH), Universitas Sam Ratulangi (Sam Ratulangi University), Manado, Indonesia; SRM College of Pharmacy (M Tovani-Palone PhD), SRM Institute of Science and Technology (SRMIST), Chennai, India; Department of Health (N M Tran MD), Children's Hospital 1, Ho Chi Minh City, Vietnam; Molecular Neuroscience Research Center (N Tran Minh Duc MD), Shiga University of Medical Science, Shiga, Japan; Department of Nursing (G M Tsegay MSc), Aksum University, Aksum, Ethiopia; Amity Institute of Biotechnology (E Upadhyay PhD), Amity University Rajasthan, Jaipur, India; College of Health and Sport Sciences (A G Vaithinathan MSc), University of Bahrain, Zallaq, Bahrain; Urmia University of Medical Sciences, Urmia, Iran (R Valizadeh PhD); UKK Institute, Tampere, Finland (Prof T J Vasankari PhD); Faculty of Medicine and Health Technology (Prof T J Vasankari PhD), Tampere University, Tampere, Finland; Key Laboratory of Computer-Aided Drug Design (M Waqas PhD), Guangdong Medical University, Dongguan, China; Department of Biotechnology and Genetic Engineering (M Waqas PhD), Hazara University Mansehra, Mansehra, Pakistan; Department of Basic Medical Sciences (S Yaghoubi PhD), Neyshabur University of Medical Sciences, Neyshabur, Iran; Biostatics, Epidemiology, and Science Computing Department (S Yezli PhD), King Faisal Specialist Hospital & Research Center, Riyadh, Saudi Arabia; Department of Epidemiology (D Yin DrPH), Xuzhou Medical University, Xuzhou, China; Department of Pediatrics (Prof D Yon MD), Kyung Hee University, Seoul, South Korea; Department of Biostatistics (Prof N Yonemoto PhD), University of Toyama, Toyama, Japan; Department of Public Health (Prof N Yonemoto PhD), Juntendo University, Tokyo, Japan; Faculty of Medicine and Health Sciences (F Zakham PhD), Hodeidah University, Hodeidah, Yemen; Department of Virology (F Zakham PhD), University of Helsinki, Helsinki, Finland; Research and Development Department (I Zare BSc), Sina Medical Biochemistry Technologies, Shiraz,

Iran; Student Research Committee (F Zarimeidani MD), Shahrekord University of Medical Sciences, Shahrekord, Iran; Department of Bioengineering and Therapeutical Sciences (Prof M Zastrozhin PhD), University of California San Francisco, San Francisco, CA, USA; Department of Administration (Prof M Zastrozhin PhD), PGxAI, San Francisco, CA, USA; School of Public Health (H Zhang MS), Peking University, Beijing, China; Department of Neurology (Y Zhao MMed), Army Medical University, Daping Hospital, Chongqing, China; Computational Bioscience Research Center (J Zhou PhD), King Abdullah University of Science and Technology, Jeddah, Saudi Arabia; Institute of Public Health and Social Sciences (H Zia BDS), Khyber Medical University, Peshawar, Pakistan; Department of Nursing (M Zoladl PhD), Yasuj University of Medical Sciences, Yasuj, Iran

Authors' Contributions

Managing the overall research enterprise

Aleksandr Y Aravkin, Simon I Hay, Nicholas J Kassebaum, Hmwe Hmwe Kyu, Mohsen Naghavi, Christopher J L Murray, Amanda Novotney, and Theo Vos.

Writing the first draft of the manuscript

Matthew C Doxey, Hmwe Hmwe Kyu, and Sarah Brooke Sirota.

Primary responsibility for applying analytical methods to produce estimates

Rose Grace Bender, and Matthew C Doxey, and Sarah Brooke Sirota.

Primary responsibility for seeking, cataloguing, extracting, or cleaning data; designing or coding figures and tables

Samuel B Albertson, Regina-Mae Villanueva Dominguez, Sarah Brooke Sirota, and Avina Vongpradith.

Providing data or critical feedback on data sources

Parsa Abdi, Richard Gyan Aboagye, Hassan Abolhassani, Lucas Guimarães Abreu, Eman Abu-Gharbieh, Salahdein Aburuz, Mesafint Molla Adane, Victor Adekanmbi, Qorinah Estiningtyas Sakilah Adnani, Leticia Akua Adzigbli, Muhammad Sohail Afzal, Saira Afzal, Bright Opoku Ahinkorah, Sajjad Ahmad, Ayman Ahmed, Haroon Ahmed, Salah Al Awaidy, Mohammed Albashtawy, Mohammad T AlBataineh, Abid Ali, Liaqat Ali, Syed Shujait Ali, Joseph Uy Almazan, Jaber S Alqahtani, Najim Z Alshahrani, Khaled Altartoor, Nelson Alvis-Guzman, Hany Aly, Safwat Aly, Hubert Amu, Saeid Anvari, Ekenedilichukwu Emmanuel Anyabolo, Jalal Arabloo, Seyyed Shamsadin Athari, Ahmed Y Azzam, Atif Amin Baig, Milica Bajcetic, Senthilkumar Balakrishnan, Maciej Banach, Mainak Bardhan, Hiba Jawdat Barqawi, Mohammad-Mahdi Bastan, Melaku Ashagrie Belete, Apostolos Beloukas, Ajay Nagesh Bhat, Mohiuddin Ahmed Bhuiyan, Aadam Olalekan Bodunrin, Hamed Borhany, Souad Bouaoud, Colin Stewart Brown, Danilo Buonsenso, Katrin Burkart, Luis Alberto Cámera, Carlos A Castañeda-Orjuela, Vijay Kumar Chattu, Hitesh Chopra, Dinh-Toi Chu, Natalia Cruz-Martins, Xiaochen Dai, Fernando Pio De la Hoz, Berecha Hundessa Demessa, Asmamaw Bizuneh Demis, Vinoth Gnana Chellaiyan Devanbu, Devananda Devegowda, Vishal R Dhulipala, Michael J Diaz, Thanh Chi Do, Thao Huynh Phuong Do, Masoud Dodangeh, Regina-Mae Villanueva Dominguez, Fariba Dorostkar, Haneil Larson Dsouza, Senbagam Duraisamy, Michael Ekholuenetale, Temitope Cyrus Ekundayo, Iman El Sayed, Faris El-Dahiyat, Mohammed Elshaer, Ugochukwu Anthony Eze, Adeniyi Francis Fagbamigbe, Folorunso Oludayo Fasina, Luisa S Flor, Santosh Gaihre, Teferi Gebru Gebremeskel, Molla Getie, Mahaveer Golechha, Pouya Goleij, Shi-Yang Guan, Mesay Dechasa Gudeta, Renu Gupta, Sapna Gupta, Najah R Hadi, Arvin Haj-Mirzaian, Rabih Halwani, Nasrin Hanifi, Zaim Anan Haq, Hadi Hassankhani, Johannes Haubold, Khezar Hayat, Praveen Hoogar,

Nobuyuki Horita, Hong-Han Huynh, Segun Emmanuel Ibitoye, Olayinka Stephen Ilesanmi, Nahlah Elkudssiah Ismail, Vinothini J, Khushleen Jaggi, Ammar Abdulrahman Jairoun, Mihajlo Jakovljevic, Shubha Jayaram, Bijay Mukesh Jeswani, Charity Ehimwenma Joshua, Jacek Jerzy Jozwiak, Zubair Kabir, Himal Kandel, Surya Kant, Rami S Kantar, Nicholas J Kassebaum, Himanshu Khajuria, Faham Khamesipour, M Nuruzzaman Khan, Maseer Khan, Min Seo Kim, Soewarta Kosen, Kewal Krishan, Hare Krishna, Vijay Krishnamoorthy, Barthelemy Kuate Defo, Ilari Kuitunen, Dewesh Kumar, Om P Kurmi, L V Simhachalam Kutikuppala, Hmwe Hmwe Kyu, Chandrakant Lahariya, Dharmesh Kumar Lal, Savita Lasrado, Huu-Hoai Le, Nhi Huu Hanh Le, Thao Thi Thu Le, Trang Diep Thanh Le, Seung Won Lee, Stephen S Lim, Gang Liu, Runben Liu, Wei Liu, Xuefeng Liu, László Lorenzovici, Lisha Luo, Kashish Malhotra, Aseer Manilal, Bharati Mehta, Tesfahun Mekene Meto, Sachith Mettananda, Le Huu Nhat Minh, Erkin M Mirrakhimov, Arup Kumar Misra, Mesud Mohammed, Mustapha Mohammed, Ali H Mokdad, Lorenzo Monasta, Mohammad Ali Moni, Catrin E Moore, Rohith Motappa, Vincent Mougin, Mohsen Naghavi, Ganesh R Naik, Mohammad Sadeq Najafi, Shumaila Nargus, Mohammad Naser, Zuhair S Natto, Biswa Prakash Nayak, Dang H Nguyen, Hau Thi Hien Nguyen, Van Thanh Nguyen, Taxiarchis Konstantinos Nikolouzakis, Ismail A Odetokun, Akinkunmi Paul Okekunle, Osaretin Christabel Okonji, Bolajoko Olubukunola Olusanya, Amel Ouyahia, Mahesh Padukudru P A, Jagadish Rao Padubidri, Ashok Pandey, Shahina Pardhan, Romil R Parikh, Ashwaghosha Parthasarathi, Maja Pasovic, Aslam Ramjan Pathan, Shankargouda Patil, Shrikant Pawar, Prince Peprah, Arokiasamy Perianayagam, Hoang Nhat Pham, Hoang Tran Pham, David M Pigott, Zahra Zahid Piracha, Maarten J Postma, Elton Junio Sady Prates, Jagadeesh Puvvula, Asma Saleem Qazi, Fakher Rahim, Vafa Rahimi-Movaghar, Mohammad Rahmanian, Masoud Rahmati, Shakthi Kumaran Ramasamy, Pushkal Sinduvadi Ramesh, Mithun Rao, Sowmya J Rao, Sina Rashedi, Nakul Ravikumar, Salman Rawaf, Luis Felipe Felipe Reyes, Nima Rezaei, Syed Mohd Danish Rizvi, Jefferson Antonio Buendia Rodriguez, Luca Ronfani, Shekoufeh Roudashti, Priyanka Roy, Basema Ahmad Saddik, Umar Saeed, Mehdi Safari, Afeez Abolarinwa Salami, Milena M Santric-Milicevic, Anudeep Sathyanarayan, Sonia Saxena, Subramanian Senthilkumaran, Yashendra Sethi, Samiah Shahid, Masood Ali Shaikh, Sunder Sham, Mohammed Shannawaz, Amin Sharifan, Javad Sharifi-Rad, Rajesh P Shastry, Aminu Shittu, Jasvinder A Singh, Paramdeep Singh, Muhammad Suleman, Chandan Kumar Swain, Lukasz Szarpak, Mohammad Tabish, Zanan Mohammed-Ameen Taha, Jabeen Taiba, Yibekal Manaye Tefera, Marcos Roberto Tovani-Palone, Ngoc Ha Tran, Nguyen Tran Minh Duc, Guesh Mebrahtom Tsegay, Era Upadhyay, Tommi Juhani Vasankari, Avina Vongpradith, Theo Vos, Ali Yadollahpour, Dong Keon Yon, Naohiro Yonemoto, Yong Yu, Ghazal G Z Zandieh, Iman Zare, Michael Zastrozhin, Yang Zhao, Juexiao Zhou, Magdalena Zielińska, and Mohammad Zoladl.

Developing methods or computational machinery

Qorinah Estiningtyas Sakilah Adnani, Saira Afzal, Najim Z Alshahrani, Walid Adnan Al-Zyoud, Hubert Amu, Aleksandr Y Aravkin, Ahmed Y Azzam, Mohammad-Mahdi Bastan, Aadam Olalekan Bodunrin, Hamed Borhany, Souad Bouaoud, Xiaochen Dai, Devananda Devegowda, Thanh Chi Do, Regina-Mae Villanueva Dominguez, Michael Ekholuenetale, Iman El Sayed, Adeniyi Francis Fagbamigbe, Shi-Yang Guan, Hong-Han Huynh, Charity Ehimwenma Joshua, Nicholas J Kassebaum, M Nuruzzaman Khan, Chandrakant Lahariya, Huu-Hoai Le, Nhi Huu Hanh Le, Thao Thi Thu Le, Le Huu Nhat Minh, Ali H Mokdad, Mohammad Ali Moni, Vincent Mougin, Christopher J L Murray, Mohsen Naghavi, Shumaila Nargus, Van Thanh Nguyen, Michal Ordak, Ashwaghosha Parthasarathi, Hoang Tran Pham, Zahra Zahid Piracha, Umar Saeed, Mehdi Safari, Austin E Schumacher, Yashendra Sethi, Mohammed Shannawaz, Muhammad Suleman, Chandan Kumar Swain, Zanan Mohammed-Ameen Taha, Yibekal Manaye Tefera, Ngoc Ha Tran, Theo Vos, Ghazal G Z Zandieh, Michael Zastrozhin, and Yang Zhao.

Providing critical feedback on methods or results

Parsa Abdi, Meriem Abdoun, Ayele Mamo Abebe, Kedir Hussein Abegaz, Richard Gyan Aboagye, Hassan Abolhassani, Lucas Guimarães Abreu, Hasan Abualruz, Eman Abu-Gharbieh, Salahdein Aburuz, Mesafint Molla Adane, Isaac Yeboah Addo, Victor Adekanmbi, Qorinah Estiningtyas Sakilah Adnani, Leticia Akua Adzigbli, Muhammad Sohail Afzal, Saira Afzal, Bright Opoku Ahinkorah, Sajjad Ahmad, Ayman Ahmed, Haroon Ahmed, Syed Anees Ahmed, Karolina Akinosoglou, Salah Al Awaidy, Samer O Alalalmeh, Mohammed Albashtawy, Mohammad T AlBataineh, Adel Ali Saeed Al-Gheethi, Robert Kaba Alhassan, Abid Ali, Liaqat Ali, Mohammed Usman Ali, Syed Shujait Ali, Waad Ali, Joseph Uy Almazan, Jaber S Alqahtani, Rami H Al-Rifai, Najim Z Alshahrani, Khaled Altartoor, Jaffar A Al-Tawfiq, Nelson Alvis-Guzman, Yaser Mohammed Al-Worafi, Hany Aly, Safwat Aly, Karem H Alzoubi, Walid Adnan Al-Zyoud, Abebe Feyissa Amhare, Hubert Amu, Ganiyu Adeniyi Amusa, Saeid Anvari, Ekenedilichukwu Emmanuel Anyabolo, Jalal Arabloo, Mosab Arafat, Demelash Areda, Seyyed Shamsadin Athari, Avinash Aujayeb, Zewdu Bishaw Aynalem, Sina Azadnajafabad, Ahmed Y Azzam, Muhammad Badar, Pegah Bahrami Taghanaki, Saeed Bahramian, Atif Amin Baig, Milica Bajcetic, Senthilkumar Balakrishnan, Maciej Banach, Mainak Bardhan, Hiba Jawdat Barqawi, Mohammad-Mahdi Bastan, Kavita Batra, Ravi Batra, Amir Hossein Behnoush, Melaku Ashagrie Belete, Apostolos Beloukas, Rose Grace Bender, Azizullah Beran, Pankaj Bhardwaj, Ashish Bhargava, Ajay Nagesh Bhat, Mohiuddin Ahmed Bhuiyan, Veera R Bitra, Aadam Olalekan Bodunrin, Eyob Ketema Bogale, Sri Harsha Boppana, Hamed Borhany, Souad Bouaoud, Colin Stewart Brown, Danilo Buonsenso, Katrin Burkart, Yasser Bustanji, Luca Cegolon, Muthia Cenderadewi, Vijay Kumar Chattu, Esther T W Cheng, Fatemeh Chichagi, Hitesh Chopra, Sonali Gajanan Choudhari, Dinh-Toi Chu, Isaac Sunday Chukwu, Erin Chung, Alexandru Corlateanu, Natalia Cruz-Martins, Omid Dadras, Tukur Dahiru, Xiaochen Dai, Jai K Das, Nihar Ranjan Dash, Mohsen Dashti, Mohadese Dashtkoohi, Fernando Pio De la Hoz, Shayom Debopadhaya, Berecha Hundessa Demessa, Asmamaw Bizuneh Demis, Vinoth Gnana Chellaiyan Devanbu, Devananda Devegowda, Kuldeep Dhama, Vishal R Dhulipala, Daniel Diaz, Michael J Diaz, Thanh Chi Do, Thao Huynh Phuong Do, Masoud Dodangeh, Regina-Mae Villanueva Dominguez, Fariba Dorostkar, Haneil Larson Dsouza, Senbagam Duraisamy, Oyewole Christopher Durojaiye, Arkadiusz Marian Dziedzic, Abdelaziz Ed-Dra, Michael Ekholuenetale, Temitope Cyrus Ekundayo, Iman El Sayed, Faris El-Dahiyat, Muhammed Elhadi, Mohammed Elshaer, Majid Eslami, Ugochukwu Anthony Eze, Adeniyi Francis Fagbamigbe, Ali Faramarzi, Florian Fischer, Luisa S Flor, Santosh Gaihre, Márió Gajdács, Nasrin Galehdar, Mohammad Arfat Ganiyani, Miglas Welay Gebregergis, Mesfin Gebrehiwot, Teferi Gebru Gebremeskel, Genanew K Getahun, Molla Getie, Keyghobad Ghadiri, Afsaneh Ghasemzadeh, Mahaveer Golechha, Anmol Goyal, Shi-Yang Guan, Mesay Dechasa Gudeta, Sapna Gupta, Veer Bala Gupta, Vivek Kumar Gupta, Mostafa Hadei, Najah R Hadi, Arvin Haj-Mirzaian, Rabih Halwani, Samer Hamidi, Ahmad Hammoud, Nasrin Hanifi, Zaim Anan Haq, Md Rabiul Haque, S M Mahmudul Hasan, Hamidreza Hasani, Md Saquib Hasnain, Hadi Hassankhani, Johannes Haubold, Simon I Hay, Khezar Hayat, Kamal Hezam, Ramesh Holla, Praveen Hoogar, Nobuyuki Horita, Mihaela Hostiuc, Hong-Han Huynh, Segun Emmanuel Ibitoye, Olayinka Stephen Ilesanmi, Irena M Ilic, Milena D Ilic, Mohammad Tarique Imam, Mustafa Alhaji Isa, Md Rabiul Islam, Sheikh Mohammed Shariful Islam, Nahlah Elkudssiah Ismail, Masao Iwagami, Vinothini J, Khushleen Jaggi, Ammar Abdulrahman Jairoun, Mihajlo Jakovljevic, Elham Jamshidi, Shubha Jayaram, Bijay Mukesh Jeswani, Ravi Prakash Jha, Jobinse Jose, Nitin Joseph, Charity Ehimwenma Joshua, Jacek Jerzy Jozwiak, Vaishali K, Zubair Kabir, Himal Kandel, Kehinde Kazeem Kanmodi, Rami S Kantar, Ibraheem M Karaye, Arman Karimi Behnagh, Nicholas J Kassebaum, Navjot Kaur, Himanshu Khajuria, Amirmohammad Khalaji, Faham Khamesipour, M Nuruzzaman Khan, Maseer Khan, Mohammad Jobair Khan, Min Seo Kim, Ruth W

Kimokoti, Sonali Kochhar, Vladimir Andreevich Korshunov, Kewal Krishan, Hare Krishna, Vijay Krishnamoorthy, Barthelemy Kuate Defo, Md Abdul Kuddus, Mohammed Kuddus, Ilari Kuitunen, Mukhtar Kulimbet, Dewesh Kumar, Om P Kurmi, L V Simhachalam Kutikuppala, Hmwe Hmwe Kyu, Chandrakant Lahariya, Dharmesh Kumar Lal, Savita Lasrado, Kaveh Latifinaibin, Huu-Hoai Le, Nhi Huu Hanh Le, Thao Thi Thu Le, Trang Diep Thanh Le, Seung Won Lee, Wei-Chen Lee, Ming-Chieh Li, Peng Li, Stephen S Lim, Gang Liu, Wei Liu, Xiaofeng Liu, Xuefeng Liu, László Lorenzovici, Lisha Luo, Azeem Majeed, Elaheh Malakan Rad, Kashish Malhotra, Iram Malik, Aseer Manilal, Bharati Mehta, Tesfahun Mekene Meto, Mathewos M Mekonnen, Hadush Negash Meles, Ziad Ahmed Memish, Max Alberto Mendez-Lopez, Sultan Ayoub Meo, Mohsen Merati, Tomislav Mestrovic, Sachith Mettananda, Le Huu Nhat Minh, Erkin M Mirrakhimov, Arup Kumar Misra, Ahmed Ismail Mohamed, Nouh Saad Mohamed, Mesud Mohammed, Mustapha Mohammed, Ali H Mokdad, Mohammad Ali Moni, AmirAli Moodi Ghalibaf, Catrin E Moore, Lidia Morawska, Rohith Motappa, Ghulam Mustafa, Mohsen Naghavi, Pirouz Naghavi, Ganesh R Naik, Firzan Nainu, Mohammad Sadeq Najafi, Soroush Najdaghi, Hastyar Hama Rashid Najmuldeen, Shumaila Nargus, Delaram Narimani Davani, Mohammad Naser, Zuhair S Natto, Biswa Prakash Nayak, Seyed Aria Nejadghaderi, Dang H Nguyen, Van Thanh Nguyen, Taxiarchis Konstantinos Nikolouzakis, Efaq Ali Noman, Chisom Adaobi Nri-Ezedi, Vincent Ebuka Nwatah, Ismail A Odetokun, Akinkunmi Paul Okekunle, Osaretin Christabel Okonji, Patrick Godwin Okwute, Titilope O Olanipekun, Isaac Iyinoluwa Olufadewa, Bolajoko Olubukunola Olusanya, Goran Latif Omer, Kenneth Ikenna Onyedibe, Michal Ordak, Nikita Otstavnov, Amel Ouyahia, Mahesh Padukudru P A, Jagadish Rao Padubidri, Ashok Pandey, Ioannis Pantazopoulos, Shahina Pardhan, Pragyan Paramita Parija, Romil R Parikh, Seoyeon Park, Ashwaghosha Parthasarathi, Maja Pasovic, Aslam Ramjan Pathan, Shankargouda Patil, Shrikant Pawar, Prince Peprah, Arokiasamy Perianayagam, Dhayaneethie Perumal, Ionela-Roxana Petcu, Hoang Nhat Pham, Hoang Tran Pham, Anil K Philip, Zahra Zahid Piracha, Roman V Polibin, Maarten J Postma, Reza Pourbabaki, Elton Junio Sady Prates, Jagadeesh Puvvula, Asma Saleem Qazi, Gangzhen Qian, Quinn Rafferty, Fakher Rahim, Mehran Rahimi, Vafa Rahimi-Movaghar, Md Obaidur Rahman, Mosiur Rahman, Muhammad Aziz Rahman, Mohammad Rahmanian, Nazanin Rahmanian, Vahid Rahmanian, Masoud Rahmati, Prashant Rajput, Mahmoud Mohammed Ramadan, Shakthi Kumaran Ramasamy, Pushkal Sinduvadi Ramesh, Indu Ramachandra Rao, Mithun Rao, Sowmya J Rao, Sina Rashedi, Mohammad-Mahdi Rashidi, Devarajan Rathish, Nakul Ravikumar, Salman Rawaf, Elrashdy Moustafa Mohamed Redwan, Luis Felipe Felipe Reyes, Nazila Rezaei, Nima Rezaei, Omid Rezahosseini, Syed Mohd Danish Rizvi, Jefferson Antonio Buendia Rodriguez, Shekoufeh Roudashti, Priyanka Roy, Basema Ahmad Saddik, Mohammad Reza Saeb, Umar Saeed, Mehdi Safari, Fatemeh Saheb Sharif-Askari, Narjes Saheb Sharif-Askari, Joseph W Sakshaug, Afeez Abolarinwa Salami, Mohamed A Saleh, Malik Sallam, Yoseph Leonardo Samodra, Rama Krishna Sanjeev, Milena M Santric-Milicevic, Benn Sartorius, Anudeep Sathyanarayan, Jennifer Saulam, Sonia Saxena, Ganesh Kumar Saya, Mansour Sedighi, Ashenafi Kibret Sendekie, Subramanian Senthilkumaran, Yashendra Sethi, SeyedAhmad SeyedAlinaghi, Mahan Shafie, Samiah Shahid, Masood Ali Shaikh, Mohammad Ali Shamshirgaran, Mohd Shanawaz, Mohammed Shannawaz, Amin Sharifan, Javad Sharifi-Rad, Rajesh P Shastry, Aziz Sheikh, Mika Shigematsu, Rahman Shiri, Aminu Shittu, Ivy Shiue, Seyed Afshin Shorofi, Emmanuel Edwar Siddig, Jasvinder A Singh, Paramdeep Singh, Robert Sinto, Sameh S M Soliman, Muhammad Suleman, Rizwan Suliankatchi Abdulkader, Chandan Kumar Swain, Lukasz Szarpak, Seyyed Mohammad Tabatabaei, Mohammad Tabish, Zanan Mohammed-Ameen Taha, Jabeen Taiba, Iman M Talaat, Jacques Lukenze Tamuzi, Birhan Tsegaw Taye, Yibekal Manaye Tefera, Mohamad-Hani Temsah, Dufera Rikitu Terefa, Ramna Thakur, Rekha Thapar, Sathish Thirunavukkarasu, Ales Tichopad, Jansje Henny Vera Ticoalu,

Marcos Roberto Tovani-Palone, Nghia Minh Tran, Ngoc Ha Tran, Nguyen Tran Minh Duc, Guesh Mebrahtom Tsegay, Munkhtuya Tumurkhuu, Aniefiok John Udoakang, Era Upadhyay, Seyed Mohammad Vahabi, Rohollah Valizadeh, Manish Vinayak, Theo Vos, Muhammad Waqas, Haftom Legese Weldetinsaa, Nuwan Darshana Wickramasinghe, Ali Yadollahpour, Saber Yezli, Dehui Yin, Naohiro Yonemoto, Yong Yu, Fathiah Zakham, Ghazal G Z Zandieh, Fatemeh Zarimeidani, Michael Zastrozhin, Chunxia Zhai, Haijun Zhang, Zhi-Jiang Zhang, Yang Zhao, Juexiao Zhou, Magdalena Zielińska, Mohammad Zoladl, and Samer H Zyoud.

Drafting the work or revising it critically for important intellectual content

Parsa Abdi, Ayele Mamo Abebe, Kedir Hussein Abegaz, Hassan Abolhassani, Lucas Guimarães Abreu, Salahdein Aburuz, Isaac Yeboah Addo, Victor Adekanmbi, Qorinah Estiningtyas Sakilah Adnani, Muhammad Sohail Afzal, Saira Afzal, Bright Opoku Ahinkorah, Ayman Ahmed, Haroon Ahmed, Syed Anees Ahmed, Mohammed Ahmed Akkaif, Salah Al Awaidy, Samer O Alalalmeh, Mohammed Albashtawy, Mohammad T AlBataineh, Fadwa Naji Alhalaiqa, Robert Kaba Alhassan, Abid Ali, Liaqat Ali, Mohammed Usman Ali, Syed Shujait Ali, Waad Ali, Jaber S Alqahtani, Ahmad Alrawashdeh, Rami H Al-Rifai, Najim Z Alshahrani, Khaled Altartoor, Jaffar A Al-Tawfiq, Nelson Alvis-Guzman, Yaser Mohammed Al-Worafi, Hany Aly, Karem H Alzoubi, Walid Adnan Al-Zyoud, Abebe Feyissa Amhare, Hubert Amu, Ganiyu Adeniyi Amusa, Abhishek Anil, Saeid Anvari, Ekenedilichukwu Emmanuel Anyabolo, Jalal Arabloo, Mosab Arafat, Brhane Berhe Aregawi, Abdulfatai Aremu, Seyyed Shamsadin Athari, Avinash Aujayeb, Zewdu Bishaw Aynalem, Sina Azadnajafabad, Ahmed Y Azzam, Muhammad Badar, Atif Amin Baig, Senthilkumar Balakrishnan, Maciej Banach, Mainak Bardhan, Hiba Jawdat Barqawi, Mohammad-Mahdi Bastan, Amir Hossein Behnoush, Maryam Beiranvand, Alemu Gedefie Belete, Apostolos Beloukas, Ashish Bhargava, Ajay Nagesh Bhat, Veera R Bitra, Hamed Borhany, Colin Stewart Brown, Danilo Buonsenso, Yasser Bustanji, Austin Carter, Carlos A Castañeda-Orjuela, Luca Cegolon, Muthia Cenderadewi, Sandip Chakraborty, Vijay Kumar Chattu, Fatemeh Chichagi, Patrick R Ching, Devasahayam J Christopher, Dinh-Toi Chu, Alexandru Corlateanu, Natalia Cruz-Martins, Sriharsha Dadana, Nihar Ranjan Dash, Mohsen Dashti, Mohadese Dashtkoohi, Fernando Pio De la Hoz, Shayom Debopadhaya, Berecha Hundessa Demessa, Asmamaw Bizuneh Demis, Vishal R Dhulipala, Daniel Diaz, Michael J Diaz, Thanh Chi Do, Masoud Dodangeh, Regina-Mae Villanueva Dominguez, Ashel Chelsea Dsouza, Haneil Larson Dsouza, Senbagam Duraisamy, Oyewole Christopher Durojaiye, Arkadiusz Marian Dziedzic, Michael Ekholuenetale, Iman El Sayed, Faris El-Dahiyat, Muhammed Elhadi, Ugochukwu Anthony Eze, Adeniyi Francis Fagbamigbe, Ali Faramarzi, Folorunso Oludayo Fasina, Nuno Ferreira, Florian Fischer, Ida Fitriana, Santosh Gaihre, Márió Gajdács, Nasrin Galehdar, Mohammad Arfat Ganiyani, Miglas Welay Gebregergis, Molla Getie, Afsaneh Ghasemzadeh, Mahsa Ghorbani, Mohamad Goldust, Giuseppe Gorini, Anmol Goyal, Shi-Yang Guan, Giovanni Guarducci, Mesay Dechasa Gudeta, Sapna Gupta, Veer Bala Gupta, Vivek Kumar Gupta, Mostafa Hadei, Najah R Hadi, Arvin Haj-Mirzaian, Rabih Halwani, Ahmad Hammoud, Nasrin Hanifi, Fahad Hanna, Zaim Anan Haq, Md Rabiul Haque, S M Mahmudul Hasan, Hamidreza Hasani, Md Saquib Hasnain, Johannes Haubold, Simon I Hay, Omar E Hegazi, Kamal Hezam, Ramesh Holla, Nobuyuki Horita, Hong-Han Huynh, Segun Emmanuel Ibitoye, Olayinka Stephen Ilesanmi, Irena M Ilic, Milena D Ilic, Mustafa Alhaji Isa, Md Rabiul Islam, Sheikh Mohammed Shariful Islam, Nahlah Elkudssiah Ismail, Abdollah Jafarzadeh, Khushleen Jaggi, Mihajlo Jakovljevic, Shubha Jayaram, Bijay Mukesh Jeswani, Ravi Prakash Jha, Jobinse Jose, Nitin Joseph, Charity Ehimwenma Joshua, Jacek Jerzy Jozwiak, Himal Kandel, Kehinde Kazeem Kanmodi, Surya Kant, Rami S Kantar, Nicholas J Kassebaum, Navjot Kaur, Himanshu Khajuria, Amirmohammad Khalaji, Gulfaraz Khan, M Nuruzzaman Khan, Maseer Khan, Mohammad Jobair Khan, Min Seo Kim, Sonali Kochhar, Kewal Krishan, Barthelemy Kuate Defo, Md

Abdul Kuddus, Mohammed Kuddus, Ilari Kuitunen, Mukhtar Kulimbet, Dewesh Kumar, Om P Kurmi, L V Simhachalam Kutikuppala, Hmwe Hmwe Kyu, Chandrakant Lahariya, Savita Lasrado, Kaveh Latifinaibin, Huu-Hoai Le, Nhi Huu Hanh Le, Thao Thi Thu Le, Runben Liu, Wei Liu, László Lorenzovici, Azeem Majeed, Elaheh Malakan Rad, Kashish Malhotra, Aseer Manilal, Bharati Mehta, Tesfahun Mekene Meto, Mathewos M Mekonnen, Hadush Negash Meles, Ziad Ahmed Memish, Max Alberto Mendez-Lopez, Sultan Ayoub Meo, Mohsen Merati, Tomislav Mestrovic, Sachith Mettananda, Le Huu Nhat Minh, Nouh Saad Mohamed, Mesud Mohammed, Mustapha Mohammed, Ali H Mokdad, Lorenzo Monasta, Mohammad Ali Moni, AmirAli Moodi Ghalibaf, Catrin E Moore, Rohith Motappa, Parsa Mousavi, Ghulam Mustafa, Mohsen Naghavi, Soroush Najdaghi, Shumaila Nargus, Delaram Narimani Davani, Mohammad Naser, Zuhair S Natto, Biswa Prakash Nayak, Seyed Aria Nejadghaderi, Dang H Nguyen, Hau Thi Hien Nguyen, Van Thanh Nguyen, Taxiarchis Konstantinos Nikolouzakis, Chisom Adaobi Nri-Ezedi, Virginia Nuñez-Samudio, Vincent Ebuka Nwatah, Ismail A Odetokun, Akinkunmi Paul Okekunle, Osaretin Christabel Okonji, Patrick Godwin Okwute, Titilope O Olanipekun, Bolajoko Olubukunola Olusanya, Kenneth Ikenna Onyedibe, Michal Ordak, Verner N Orish, Esteban Ortiz-Prado, Nikita Otstavnov, Amel Ouyahia, Mahesh Padukudru P A, Jagadish Rao Padubidri, Ashok Pandey, Ioannis Pantazopoulos, Shahina Pardhan, Romil R Parikh, Shankargouda Patil, Shrikant Pawar, Arokiasamy Perianayagam, Dhayaneethie Perumal, Ionela-Roxana Petcu, Hoang Nhat Pham, Hoang Tran Pham, Anil K Philip, Zahra Zahid Piracha, Dimitri Poddighe, Maarten J Postma, Reza Pourbabaki, Elton Junio Sady Prates, Asma Saleem Qazi, Fakher Rahim, Mehran Rahimi, Vafa Rahimi-Movaghar, Mohammad Rahmanian, Masoud Rahmati, Prashant Rajput, Mahmoud Mohammed Ramadan, Shakthi Kumaran Ramasamy, Pushkal Sinduvadi Ramesh, Mithun Rao, Sowmya J Rao, Devarajan Rathish, Nakul Ravikumar, Salman Rawaf, Elrashdy Moustafa Mohamed Redwan, Luis Felipe Felipe Reyes, Nazila Rezaei, Nima Rezaei, Jefferson Antonio Buendia Rodriguez, Luca Ronfani, Guilherme de Andrade Ruela, Basema Ahmad Saddik, Umar Saeed, Pooya Saeedi, Mehdi Safari, Fatemeh Saheb Sharif-Askari, Narjes Saheb Sharif-Askari, Amirhossein Sahebkar, Monalisha Sahu, Nasir Salam, Afeez Abolarinwa Salami, Malik Sallam, Milena M Santric-Milicevic, Aswini Saravanan, Anudeep Sathyanarayan, Jennifer Saulam, Sonia Saxena, Ganesh Kumar Saya, Benedikt Michael Schaarschmidt, Yashendra Sethi, Mahan Shafie, Samiah Shahid, Mohd Shanawaz, Mohammed Shannawaz, Amin Sharifan, Javad Sharifi-Rad, Rajesh P Shastry, Mika Shigematsu, Aminu Shittu, Seyed Afshin Shorofi, Emmanuel Edwar Siddig, Colin R Simpson, Jasvinder A Singh, Paramdeep Singh, Surjit Singh, Robert Sinto, Ranjan Solanki, Sameh S M Soliman, Muhammad Suleman, Chandan Kumar Swain, Zanan Mohammed-Ameen Taha, Iman M Talaat, Jacques Lukenze Tamuzi, Birhan Tsegaw Taye, Yibekal Manaye Tefera, Mohamad-Hani Temsah, Dufera Rikitu Terefa, Ramna Thakur, Sathish Thirunavukkarasu, Ales Tichopad, Marcos Roberto Tovani-Palone, Nghia Minh Tran, Guesh Mebrahtom Tsegay, Aniefiok John Udoakang, Era Upadhyay, Asokan Govindaraj Vaithinathan, Tommi Juhani Vasankari, Manish Vinayak, Nuwan Darshana Wickramasinghe, Ali Yadollahpour, Sajad Yaghoubi, Saber Yezli, Dong Keon Yon, Naohiro Yonemoto, Ghazal G Z Zandieh, Iman Zare, Fatemeh Zarimeidani, Michael Zastrozhin, Chunxia Zhai, Haijun Zhang, Hafsa Zia, Magdalena Zielińska, Mohammad Zoladl, and Samer H Zyoud.

Managing the estimation or publications process

Simon I Hay, Nicholas J Kassebaum, Hmwe Hmwe Kyu, Ali H Mokdad, Christopher J L Murray, Mohsen Naghavi, and Maja Pasovic.
